# The Anthraquinone Derivatives from the Fungus *Alternaria* sp. XZSBG-1 from the Saline Lake in Bange, Tibet, China

**DOI:** 10.3390/molecules191016529

**Published:** 2014-10-14

**Authors:** Bin Chen, Qiong Shen, Xun Zhu, Yongcheng Lin

**Affiliations:** 1 School of Chemistry and Chemical Engineering, Sun Yat-sen University, Guangzhou 510275, China; E-Mail: chenyishan@126.com; 2 Tibet Plateau Institute of Biology, Lhasa 850001, China; 3 Zhongshan School of Medicine, Sun Yat-sen University, Guangzhou 510275, China; E-Mail: zhuxun8@mail.sysu.edu.cn

**Keywords:** *Alternaria*, secondary metabolites, anthraquinones, alterporriols, altersolanols, cytotoxicity, enzyme inhibitory activity

## Abstract

Four new anthraquinone derivatives **1**–**4** were obtained along with seven known compounds **5**–**11** from the extracts of the fungal strain *Alternaria* sp. XZSBG-1 which was isolated from the sediments of the carbonate saline lake in Bange, Tibet, China. Their structures were determined by spectroscopic methods, mainly by 2D NMR spectra. Compound **1** is a novel tetrahydroanthraquinone with an epoxy ether bond between C-4a and C-9a. In the primary bioassays, compound **3** (alterporriol T) exhibited inhibition of a-glucosidase with a IC_50_ value 7.2 μM, and compound **9** showed good inhibitory activity against the HCT-116 and HeLa cell lines, with IC_50_ values of 3.03 and 8.09 μM, respectively.

## 1. Introduction

In 2002, we reported research on the metabolites of a fungal strain from a saline lake locates in the Bahamas [1]. Since that time, it seems that that little research on fungal metabolites from saline lakes has been published. Recently, we have been interested in the microorganisms from Tibetan saline lakes, which live under the special plateau habitat conditions, including low temperatures and high salt levels that are similar to those of the ocean, and we have thus obtained some unique and significant compounds.

**Figure 1:**
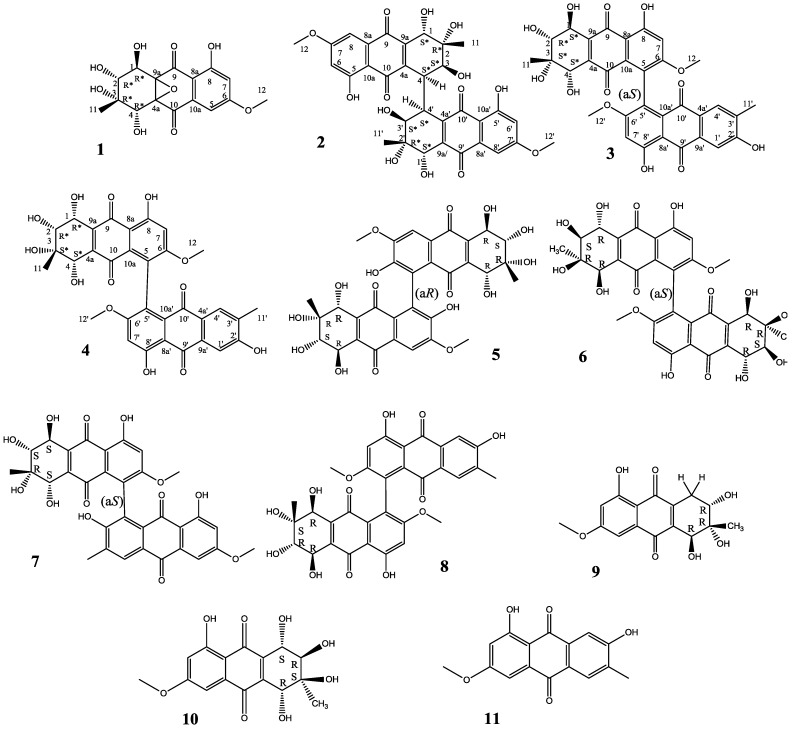
Structures of compounds **1**–**11** isolated from *Alternaria* sp. XZSBG-1.

The fungus *Alternaria* sp. XZSBG-1 collected from the sediment of the salt lake in Bange, Tibet, China was studied. We found that this fungus contained abundant anthraquinone compounds. Four new anthraquinone and tetrahydroanthraquinone derivatives **1**–**4**, were isolated from this fungus along with seven known compounds (**5**–**11**) (Figure 1). Compound **1**, a novel tetrahydroanthraquinone with an epoxy ether bond between C-4a and C-9a, and compound **2**, a tetrahydroanthraquinone dimer with a C-4-C-4' linkage, are rare.

Anthraquinones and tetrahydroanthraquinones are widely distributed as secondary metabolites in natural biosources, and show important biological activities [2,3,4,5]. So far, 18 compounds of alterporriol family [6,7,8,9,10,11,12] and 14 compounds of altersolanol family [9,13,14,15,16] have been reported. Herein, we report that the isolation, elucidation and biological activities of the anthraquinone and tetrahydroanthraquinone derivatives from *Alternaria* sp. XZSBG-1.

## 2. Results and Discussion

**Figure 2:**
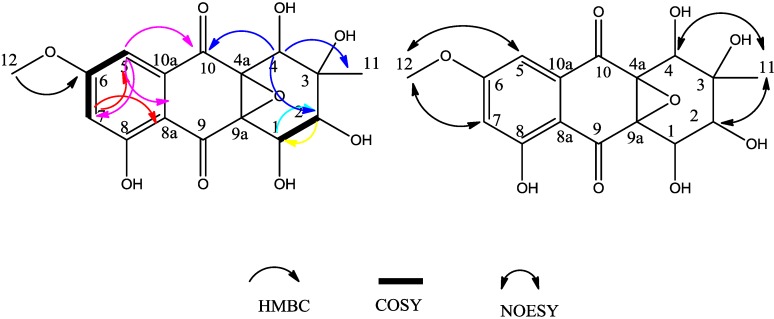
HMBC correlations and selected NOESY correlations of compound **1**.

Compound **1** was yellow, amorphous powder. A HR-ESI-TOF-MS peak at *m/z* = 375.0688 (calcd. for C_16_H_15_NaO_9_, 375.0687) indicated the molecular formula C_16_H_16_O_9_. The ^1^H-NMR spectrum (Table 1) showed two aromatic protons (δ_H_ = 6.91 ppm and δ_H_ = 6.81 ppm), one methoxyl (δ_H_ = 3.86 ppm), one methyl group singlet (δ_H_ = 1.12 ppm), three oxygenated methine groups (δ_H_ = 4.43, 4.42 and 3.27 ppm) and a chelated hydroxyl (δ_H_ = 11.16 ppm) resonances. The ^13^C-NMR spectrum (Table 1) displayed two carbonyl signals (δ_C_ = 193.61, 191.12 ppm), six aromatic carbon signals, including three quaternary carbons (δ_C_ = 74.14, 68.37 and 67.16 ppm) and three methines (δ_C_ = 71.84, 67.64 and 67.28 ppm), one methoxyl group (δC = 56.43 ppm) and one methyl group (δ_C_ = 21.78 ppm). These data implied that compound **1** possessed a tetrahydroanthraquinone skeletone (Figure 2). In compound **1**, the two aromatic protons (δ_C_ = 106.19 ppm; δ_H_ = 6.91 ppm, d, *J* = 2.48 Hz) and (δ_C_ = 106.98 ppm; δ_H_ = 6.81 ppm, d, *J* = 2.48 Hz) were at *meta* positions from each other on the aromatic ring, based on their 2.48 Hz coupling constant; The HMBC correlations from H-5 to C-6, C-7, C-8a and C-10, combined with from H-7 to C-5, C-6, C-8 and C-8a, and from H-12 (methoxy) to C-6, in addition, the NOE correlations of H-12(OCH_3_) to H-5 and H-6 (that supported the deduction that OCH_3_-12 is attached to C-6), established the substitution pattern of the aromatic ring (Figure 2). The protons in the cyclohexane ring, including three oxygenated methines H-1 (δ_H_ = 4.42 ppm, d, *J* = 8.1 Hz), H-2 (δ_H_ = 3.27 ppm, d, *J* = 8.1 Hz) and H-4 (δ_H_ = 4.43 ppm, s) were also observed. The coupled signals from H-1 to H-2 in the 1H-1H COSY spectrum combined with the HMBC correlations from H-1 to C-2, and from H-4 to C-2, C-3, C-4a, C-10 and C-11, established the substructure of the cyclohexane ring (Figure 2).

The HMBC correlations from H-11 to C-2, C-3 and C-4 indicated the methyl group was linked to C-3. One methoxy group, two carbonyl groups and five hydroxyl groups all together occupied eight oxygen atoms, so the remaining two carbons C-4a (δ_C_ = 68.37 ppm) and C-9a (δ_C_ = 67.16 ppm) must combine with the remaining oxygen to form an epoxy ether bond.

The relative configuration of the chiral centers of C-1, C-2, C-3 and C-4 were deduced by 2D ^1^H-^1^H NOESY experiments (Figure 2). A NOESY correlation among CH_3_-11 (δ_H_ = 1.12, singlet), H-2 and H-4 suggested that they are on the same side of the cyclohexane ring, and this was supported by the correlation between H-4 and H-2 (Figure 2). The coupling constant 8.1 Hz between H-1 and H-2 implied that H-1 and H-2 were positioned in a pseudoaxial orientation from each other. We could not deduce the relative configuration of the chiral centers of C-4a and C-9a by NOESY experiments or other spectroscopic methods other than X-ray single crystal diffraction. Therefore, compound **1** was determined as (1R*,2R*,3R*,4R*)-1,2,3,4,8-pentahydroxy-6-methoxy-3-methyl-1,2,3,4-tetrahydro-4a,9a-epoxyanthracene-9,10-dione. We propose for this compound the trivial name altersolanol O.

**Table 1:** NMR data of compounds **1** and **2** (DMSO-*d*_6_), measured at 400 MHz (^1^H) and 100 MHz (^13^C).

Position	Compound 1			Position	Compound 2		
	δ_C_, (ppm)	δ_H_ (ppm) (mult., J in Hz)	HMBC		δ_C_, (ppm)	δ_H_ (ppm) (mult., J in Hz)	HMBC
1	67.28	4.42 d (8.1)	C-2	1,1'	67.78	4.38 d (4.32)	C-2, 3, 4a, 9, 9a, 11
2	71.84	3.27 d (8.1)	C-1	2,2'	71.69		
3	74.14			3,3'	69.59	3.77 br. d (7.8,4.8 overlap)	C-4
4	67.64	4.43 s	C-2, 3, 10, 11	4,4'	42.7	3.82 dd (1.3, 4.8)	C-3, 4', 4a, 9a
4a	68.37			4a,4a'	149.43		
5	106.19	6.91 d (2.48)	C-6, 7, 8a, 10	5,5'	163.04		
6	165.49			6,6'	105.49	6.80 d (2.5)	C-5, 7, 8, 10a
7	106.98	6.81 d (2.48)	C-5, 6, 8, 8a	7,7'	164.89		
8	162.11			8,8'	105.63	7.05 d (2.5)	C-6, 7, 9, 10a
8a	110.0			8a,8a'	133.69		
9	193.61			9,9'	183.08		
9a	67.16			9a,9a'	140.62		
10	191.12			10,10'	188.59		
10a	134.46			10a,10a'	109.91		
11	21.78	1.12 s	C-2, 3, 4	11,11'	22.22	1.13 s	C-1, 2, 3
12	56.43	3.86 s	C-6	12,12'	56.08	3.91 s	C-7
1-OH				1,1'-OH		5.12 d (4.32)	C-1, 2, 9a
2-OH				2,2'-OH		4.14 s	C-1
3-OH				3,3'-OH		4.09 d (7.8)	C-3, 4
4-OH				5,5'-OH		12.26 s	C-5, 6, 10a
8-OH		11.16 s					

**Figure 3:**
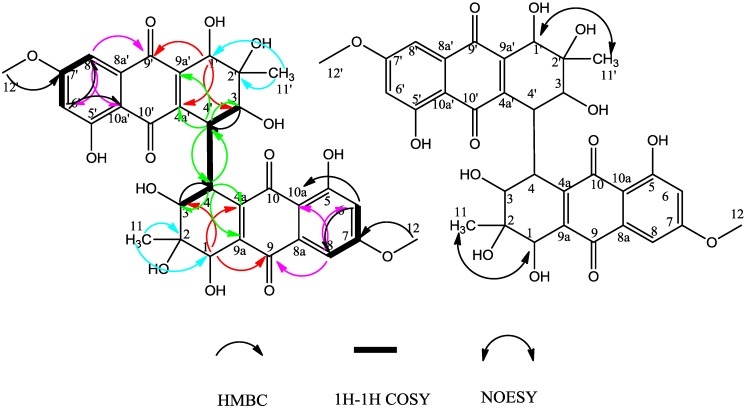
HMBC correlations and selected NOESY correlations of compound **2**.

Compound **2** was obtained as a red, amorphous powder. The HR-ESI-TOF-MS peak at *m/z* = 637.1559 [M−H]^−^ (calcd. For C_32_H_29_O_14_, 637.1563) indicated the molecular formula C_32_H_30_O_14_. According to the ^1^H and ^13^C-NMR spectra (Table 1), we concluded that compound **2** was a symmetrical tetrahydroanthraquinone dimer. Compared to the reported compound alterporriol O [12], their ^1^H and ^13^C-NMR spectra were almost identical, but there were notable differences between the corresponding basic UV and optical rotation spectra, whereby the specific rotation value of compound **2** was large (−1200 in acetone) while that of the alterporriol O was −39.0 (in acetone). The NOESY correlation (Figure 3) between CH_3_-11 (δ_H_ = 1.13 ppm, s) and H-1 implied that they were also on the same side of the cyclohexene ring, unlike in alterporriol O. Therefore, compound **2** was identified as (1S*,1'S*,2R*,2'R*,3S*,3'S*,4S*,4'S*)-1,1',2,2',3,3',5,5'-octahydroxy-7,7'-dimethoxy-2,2'-dimethyl-1,1',2,2',3,3',4,4'-octahydro-[4,4'-bianthracene]-9,9',10,10'-tetraone. We propose for this new compound the trivial name alterporriol S.

**Figure 4:**
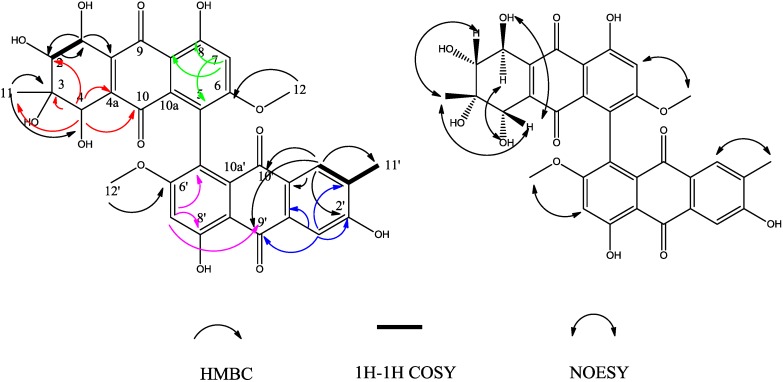
HMBC correlations and selected NOESY correlations of compound **3**.

Compound **3** was obtained as a red, amorphous powder. The HR-ESI-TOF-MS peak at *m/z* = 617.1279 [M−H]^−^ (calcd. for C_32_H_25_O_13_, 617.1301) indicated the molecular formula C_32_H_26_O_13_. The ^1^H and ^13^C-NMR spectra (Table 2) were reminiscent of those obtained for the known monomeric compounds **10** and **11**, which suggested that compound **3** is a modified bisanthraquinone dimer. We could readily deduce that compound **3** included one tetrahydroanthraquinone unit and one anthraquinone unit. On the anthraquinone unit, two aromatic proton singlet signals for H-1' (δ_H_ = 7.70 ppm, s) and H-4' (δ_H_ = 7.56 ppm, s) implied that H-1' and H-4' were in the *para* position from each other; a chelated hydroxyl signal (δ_H_ = 13.60 ppm, s) was observed and HMBC correlations from OH-8' (δ_H_ = 13.60 ppm) to C-8a', C-8', C-7' and from OCH_3_-12' (δ_H_ = 3.68 ppm) to C-6' all these indicated the anthraquinone unit to be a macrosporin unit. Furthermore, the HMBC correlations and NOESY correlations (Figure 4) of this unit might indicated that the unit was macrosporin.

In the tetrahydroanthraquinone unit, the HMBC correlations from H-1 to C-2 and C-9a, and from H-4 to C-2, C-3, C-4a, C-10 and C-11, revealed the cyclohexene ring substructure (Figure 4). The chelated hydroxyl signal (δ_H_ = 13.04 ppm, s) was observed with its HMBC correlations to C-8a, C-8, C-7, correlations from H-7 to C-5, C-8, C-8a and from OCH_3_-12 (δ_H_ = 3.70 ppm, s) to C-6, and combining with NOESY data, these established the substructure of the aromatic ring (Figure 4).

**Table 2:** NMR data of compound **3** and **4** (DMSO-*d*_6_), measured at 400 MHz (^1^H) and 100 MHz (^13^C).

Position	Compound 3				Compound 4			
	δ_C_, (ppm)	δ_H_ (ppm) (mult., J in Hz)	HMBC	NOE	δ_C_, (ppm)	δ_H _(ppm) (mult., J in Hz)	HMBC	NOE
1	68.39	4.47 dd (5.78, 7.06)	C-2, 9a	H-4-OH	68.45	4.48 dd (5.60, 6.87)	C-2, 4a, 9a, 9	H-4
2	73.78	3.57 dd (7.06, 7.06)	C-1	H-11	73.74	3.55 dd (6.87, 6.87)	C-1	H-11
3	72.83				72.87			
4	68.22	4.03 d (6.93)	C-2, 3, 4a, 10, 11	H-11,1-OH	68.27	4.05 d (6.77)	C-2, 9a, 10, 4a, 11	H-1, 11
4a	143.31				143.42			
5	122.86				122.59			
6	164.28				164.33			
7	103.77	6.93 s	C-5, 8, 8a		103.77	6.92 s	C-6, 8, 8a, 9	H-12'
8	163.63				163.73			
8a	109.27				109.3			
9	188.77				188.79			
9a	142.78				142.61			
10	184.09				183.88			
10a	128.86				129.02			
11	22.18	1.13 s	C-3, 4	H-2,4,1-OH	22.22	1.13 s	C-3, 4	H-2, 4
12	56.74	3.70 s	C-6	H-7	56.71	3.69 s	C-6	H-7
1-OH		5.64 d (7.06)	C-1, 3, 9a	H-4		4.98 d (5.60)	C-1, 2, 9a	H-4-OH
2-OH		4.36 s	C-2, 4			4.81 d (6.87)	C-1, 2, 3	H-4-OH
3-OH		4.85 d (6.93)	C-1			4.38 s	C-2, 4, 11	H-1-OH
4-OH		5.04 d (5.78)	C-2, 4, 4a	H-1		5.44 d (6.77)	C-3, 4, 4a	H- 2-OH
8-OH		13.04 s	C-7, 8, 8a			13.04 s	C-7, 8, 8a	
1'	110.44	7.56 s	C- 2', 3', 9', 9a'		110.42	7.55 s	C-2', 3', 9', 9a', 10'	H-2'-OH
2'	161.19				161.24			
3'	125.25				125.27			
4'	130.18	7.70 d (0.56)	C-3', 4a', 10', 11'	H-11'	130.33	7.70 d (0.69)	C-2', 4a', 9', 10', 11'	H-11'
4a'	132.39				132.38			
5'	121.81				123.12			
6'	164.18				165.25			
7'	104.01	6.94 s	C- 5', 8', 8a', 9'		103.54	6.94 s	C- 6, 8', 8a', 9'	H-8'-OH
8'	164.85				165.23			
8a'	109.98				109.92			
9'	186.7				186.61			
9a'	132.2				132.2			
10'	181.1				180.97			
10a'	131.48				130.43			
11'	16.03	2.20 s	C-2',4'	H-4'	16.09	2.19 s	C-2', 3', 4'	H-4'
12'	56.65	3.68 s	C-6'	H-7'	56.83	3.72 s	C-6’	H-7'
2'-OH		11.07 br. s				11.03 br. s		H-1'
8'-OH		13.60 s	C-7', 8', 8a'			13.66 s	C-7', 8', 8a'	H-7'

The HMBC correlation between H-12(OCH_3_) and C-6 indicated that the OCH_3_ attached at C-6. Further, the HMBC correlations from H-7 to C-5, C-8, C-8a, especially the NOE correlation between OCH_3_-12 and H-7, established that the junction between the two moieties of **3** was between C-5 and C-5' Besides, based on the HMBC correlations from H-7 to C-12, from H-4' to C-11', from H-7' to C-12' located the positions of three methyl groups.

The relative configuration of the chiral centers of C-1, C-2, C-3 and C-4 were deduced by 2D ^1^H-^1^H NOESY experiments (Figure 4). The NOESY correlations from CH_3_-11 (δ_H_ = 1.13 ppm, s) to H-2 and H-4, from H-1 to OH-4, from H-4 to OH-1, suggested that H-2, H-4, OH-1 and CH_3_-11 were on the same side of the cyclohexene ring, the rest (H-1, OH-2, OH-3 and OH-4) were on the other side of cyclohexene ring (H-1, OH-2, OH-3 and OH-4); finally compound **3** was determined as (1S*,2R*,3S*,4S*)-1,2,2',3,4,8,8'-heptahydroxy-6,6'-dimethoxy-3,3'-dimethyl-1,2,3,4-tetrahydro-[5,5'-bianthracene]-9,9',10,10'-tetraone. We propose for this compound the trivial name alterporriol T.

**Figure 5:**
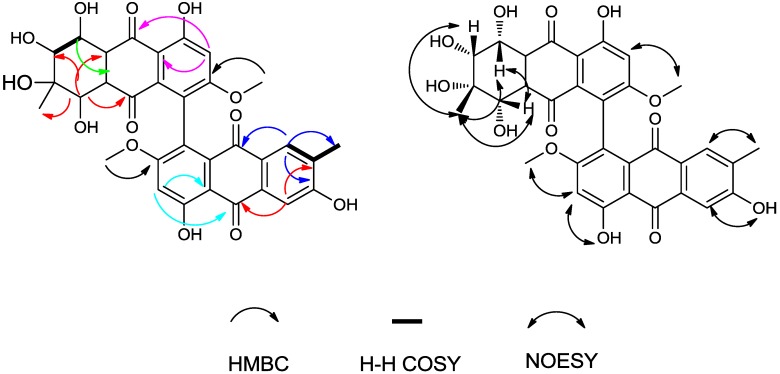
HMBC correlations and selected NOESY correlations of compound **4**.

Compound **4** was also obtained as a red, amorphous powder. The HR-ESI-TOF-MS peak at *m/z* = 617.1282 (calcd. for C_32_H_25_O_13_, 617.1301) indicated the molecular formula C_32_H_26_O_13_. The ^1^H- and ^13^C-NMR spectra of compounds **3** and **4** were very similar. In the ^13^C-NMR spectrum, only three carbons’ chemical shifts were slightly different, they were C-5', C-6', C-8' (Table 2). The HMBC correlations indicated that compounds **3** and **4** had a same planar configuration. However, the specific rotation value of the two compounds were very different (compound **3**, −39.95; compound **4**, −337; in ethanol), and their CD spectra were also very different (Figure 5). All these suggested that they were isomers and the relative configuration of compound **4** was different from that of compound **3**. In the NOESY correlation spectra, the correlations from CH_3_-11 (δ_H_ = 1.13, s) to H-1, H-2 and H-4 suggested that H-1, H-2, H-4 and CH_3_-11 were on the same side of the cyclohexene ring.

NOESY correlations of the anthraquinone unit (from H-1' to OH-2', from H-4' to CH_3_-11', from H-7' to OCH_3_-6', and from H-7' to OH-8') were also observed. Finally, we could elucidate the structure of compound **4** as (1R*,2R*,3S*,4S*)-1,2,2',3,4,8,8'-heptahydroxy-6,6'-dimethoxy-3,3' dimethyl-1,2,3,4-tetrahydro-5,5'-bianthracene-9,9',10,10'-tetraone. We propose for this compound the trivial name alterporriol V.

**Figure 6:**
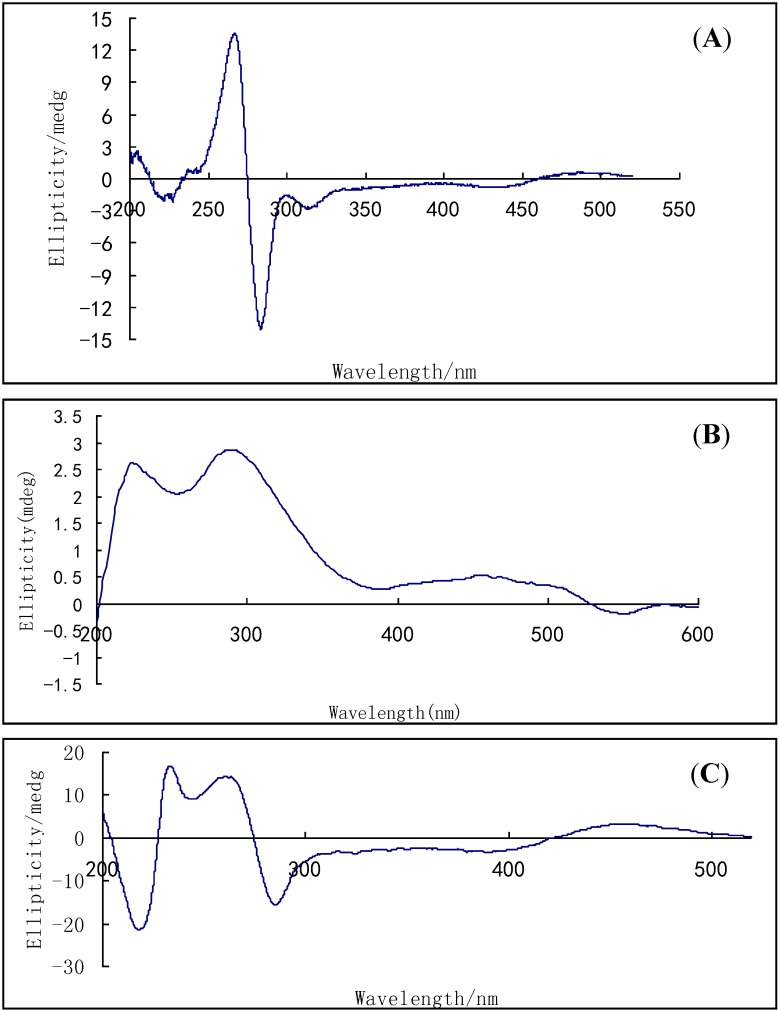
CD spectra of compounds **3** (**A**), **4** (**B**) and **7** (**C**, alterporriol N) in acetonitrile solution.

Compounds **3** and **4** also contain a chiral axis. Comparing the CD spectra (Figure 6) with that of the the known compound alterporriol N, because the CD spectra were less sensitive to the configuration of the four chiral centres [10,16], according to the trends of CD between compound **3** and alternporriol N, we suggest that the absolute configuration of the chirality axis for compound **3** can be assigned as a*S*.

The compounds **5**–**11** were identified by comparing their spectroscopic data with those of the corresponding known compounds.

Compounds **1**–**11** were evaluated for cytotoxic activity against several human cancer cell lines by MTT assay [17,18]. The results showed that compound **9** showed good inhibitory activity against HCT-116 and HeLa cell lines, the IC_50_ values are 3.03 and 8.09 μM, respectively (Table 3). The other compounds show no notable inhibitory activity against any tested cancer cell lines.

**Table 3:** Cytotoxicity test with anti-tumor, Epirubicin as a positive control.

Compound	IC_50_ (μM)		
	MCF-7/ADR	HCT-116	HeLa
1	18.48	23.24 ± 2.85	43.74 ± 4.02
2	>100	>50	>50
3	ND	32.38 ± 1.57	>50
4	>50	>50	>100
5	>100	>100	>100
6	>100	>100	>100
7	>100	>100	>100
8	44.99	>50	>50
9	>100	3.03 ± 0.05	8.09 ± 0.89
10	>100	>100	>100
11	>100	>100	>100
EPI	2.36	0.96 ± 0.02	0.48 ± 0.03

Note: ND—No detected.

Compounds **1**–**11** were also tested for their ability to inhibit α-glucosidase by a described method [19]. Compound **3** (Alterporriol T) shows good inhibitory activity on α-glucosidase, with an IC_50_ value 7.2 μM (Table 4). The other compounds show no inhibitory activity against α-glucosidase.

**Table 4:** The inhibition activities on α-glucosidase for selected compounds.

Comp.	1	2	3	4	5	6	7	8	9	10	11	Genistein *
IC_50_ (μM)	>100	72.0	7.2	>100	>400	>100	>100	>100	>100	>100	83.5	13.6

Note: * Genistein—positive control.

## 3. Experimental Section

### 3.1. General Procedures

Column chromatography (CC) was performed using silica gel (200–300 mesh, Qingdao Marine Chemical, Qingdao, China). The HPLC system consisted of a Waters 2010 series (Waters, Milford, MA, USA). A mini ODS column (250 × 10 mm, 10 μm particle size) was used. Melting points were determined on an X-4 micro-melting point apparatus and were uncorrected. Circular dichroism was measured on a Schmidt Haensch Polartronic HH W5 polarimeter (Schmidt, Germany) and was uncorrected. UV spectra were measured on a Shimadzu UV-3501 PC spectrophotometer (Shimadzu, Japan). ^1^H (400 MHz) and ^13^C-NMR (100 MHz) data were recorded on a Bruker AVANCE 400 spectrometer (Bruker, Switzerland) with TMS as internal standard. LC/MS data were acquired using an Applied Biosystems/MDS Sciex (Applied Biosystems, Grand Island, NY, USA) and ana ESI source. HR-ESIMS were measured on a Shimadzu LCMS-IT-TOF.

### 3.2. Fungal Material

The fungal strain *Alternaria* sp. XZSBG-1 was isolated from a piece of sediment involving rotten branches and leaves, which was collected from the saline carbonate lake of Bamucuo, in Bange county, Tibetan Autonomous Region, China in July 2007. The strain was deposited in the Guangdong Province Key Laboratory of Functional Molecules in Oceanic Microorganism, School of Chemistry and Chemical Engineering, Sun Yat-sen University of China, Guangzhou, China.

### 3.3. Identification of Fungal Cultures

The fungal strain was identified as an *Alternaria* sp. according to morphologic traits and a molecular biological protocol by DNA amplification and sequencing as described [12,20]. The sequence data have been submitted to GenBank, accession number HM622756, and the strain was identified as *Alternaria* sp. Its 591 base pair ITS sequence had 99% sequence identity to that of *Alternaria* sp. IA249 (AY154699).

### 3.4. Fermentation, Extraction, and Isolation of Alternaria sp. XZSBG-1

The fungal strain was cultivated in potato glucose liquid medium (15 g of glucose and 3 g of crude sea salt in 1 L of potato infusion) in 1 L Erlenmeyer flasks each containing 400 mL of culture broth at 25 °C without shaking for 4 weeks. The fermentation broth (80 L) was filtered. The culture broth was extracted three times with an equal volume of EtOAc. The combined EtOAc layers were evaporated to dryness under reduced pressure to give an EtOAc extract (25.2 g). The mycelia was extracted with MeOH three times. The MeOH layer was evaporated under vacuum; then the combined residue was suspended in H_2_O and partitioned with EtOAc to provide the EtOAc extract (28.0 g), all the crude extract combined together (53.2 g) was subjected to silica gel column chromatography (CC, petroleum ether, EtOAc v/v, gradient) to generate eight fractions (Fraction 1−8). Fraction 4 was isolated by CC on silica gel eluted with petroleum ether−EtOAc (*v*/*v*, 10:1, 8:1, 6:1, 4:1, 2:1) and then subjected to Sephadex LH-20 CC eluting with mixtures of petroleum ether−CHCl_3_−MeOH (2:1:1) to obtain macrosporin (**11**, 3.0 g). Fraction 5 was subjected to repeated silica gel CC (CHCl_3_−MeOH, *v*/*v*, 50:1, 40:1, 30:1, 20:1, 15:1, 10:1, 9:1, 8:1, 7:1, 6:1, 5:1) to obtain altersolanol A (**10**, 8.0 mg), altersolanol **C** (**9**, 3.0 mg), altersolanol O (**1**, 2.4 mg), alterporriol A (**8**, 23.0 mg), alterporriol N (**7**, 20.6 mg), alterporriol **S** (**2**, 2.0 mg), fraction 9, and fraction 10, respectively. Fr. 8 was subjected to repeated Sephadex LH-20 CC (MeOH) and further purified on HPLC (40% MeOH−H_2_O) to afford alterporriol **E** (**5**, 18.0 mg) and alterporriol D (**6**, 11.0 mg). Fr.9 was subjected to repeated silica gel CC (CHCl_3_−MeOH, v/v, 100:1, 80:1, 60:1, 40:1, 20:1, 15:1, 10:1) to obtain alterporriol U (**3**, 22 mg); fraction 10 was purified on HPLC (55% MeOH−H_2_O) to afford alterporriol V (**4**, 16 mg).

*Altersolanol O* (**1**): yellow, amorphous powder; ${\left[\alpha \right]}_{D}^{18}$ = −29.46 (c = 0.34, MeOH); UV (MeOH) λmax (log ε) = 289 (1.03), 375 (0.40) nm; ^1^H-NMR (DMSO-*d*_6_), see Table 3; ^13^C-NMR (DMSO-*d*_6_), see Table 3; ESIMS *m/z* 351.0 [M−H]^−^; HRESIMS *m/z* 375.0688 (calcd. for C_16_H_15_NaO_9_, 375.0687).

*Alterporriol S* (**2**): red, amorphous powder; ${\left[\alpha \right]}_{D}^{27}$ = −1200 (c = 0.2, MeOH); UV (ethanol) λmax (log ε) = 258 (3.99), 396 (0.597) nm; ^1^H-NMR (DMSO-*d*_6_), see Table 3; ^13^C-NMR (DMSO-*d*_6_), see Table 3; ESIMS *m/z* 637.0 [M−H]^−^; HRESIMS *m/z* 637.1559 (calcd. for C_32_H_29_O_14_, 637.1563).

*Alterporriol T* (**3**): red, amorphous powder; ${\left[\alpha \right]}_{D}^{18}$ = −39.95 (c = 2.5, MeOH); UV (ethanol) λmax (log ε) = 248 (4.0), 437 (0.542); ^1^H-NMR (DMSO-*d*_6_), see Table 2; ^13^C-NMR (DMSO-*d*_6_), see Table 2; ESIMS *m/z* 617.0 [M−H]^−^; HRESIMS *m/z* 617.1279 (calcd. for C_32_H_25_O_13_, 617.1301).

*Alterporriol U* (**4**): orange, amorphous powder; ${\left[\alpha \right]}_{D}^{18}$ = −337 (c = 1.99, MeOH); UV (ethanol) λmax (log ε) = 254 (3.90), 436 (0.722) nm; ^1^H-NMR (DMSO-*d*_6_), see Table 3; ^13^C-NMR (DMSO-*d*_6_), see Table 3; ESIMS *m/z* 617.0 [M−H]^−^; HRESIMS *m/z* 617.1282 (calcd. for C_32_H_25_O_13_, 617.1301).

### 3.5. Biological Assays

#### 3.5.1. Antitumor Activity *in Vitro*

##### Cell Culture

MCF-7/ADR, HeLa, HCT-116 cell lines were cultured in Dulbeccos’ modification Eagle’s medium (DMEM, Invitrogen, Carlsbad, CA, USA) supplemented with 10% fetal bovine serum (FBS, Hyclone, Logan, UT, USA), 2 mM L-glutamine, 100 μg/mL streptomycin and 100 U/mL penicillin (Invitrogen). The cells were incubated at 37 °C in a humidified atmosphere with 5% CO_2_.

##### Assessment of Antitumor Activity by MTT Assay

Cells were harvested during logarithmic growth phase and seeded in 96-well plates at a density of 1 × 10^4^ cells/mL, and cultured at 37 °C in a humidified incubator (5% CO_2_) for 24 h, followed by exposure to various concentrations of compounds tested for 48 h. Subsequently 20 μL of MTT (Genview, Houston, TX, USA) solution (5 mg/mL) was added to each well and mixed, the cells were then incubated for an additional 4 h. Culture supernatant was moved, 150 μL of DMSO (Sangon Biotech, Shanghai, China) was added to each well to fully dissolve the MTT-formazan crystals. Cell growth inhibition was determined by measuring the absorbance (Abs) at λ = 570 nm using a microplate reader and calculated according to the following equation:

Growth inhibition = (1 − OD of treated cells/OD of control cells) × 100%

The half maximal inhibitory concentrations (IC_50_) were obtained from liner regression analysis of the concentration-response curves plotted for each tested compound [17,18].

#### 3.5.2. Enzyme Assays

Alpha-glucosidase activity was assayed using 50 mM phosphate buffer at pH 7.0, and the appropriate PNP glycoside (at 1 mM) were used as substrates. The concentration of the enzyme was specified in each experiment. Curcuminoids at the designated concentration was added to the enzyme solution and incubated at 37 °C for 30 min, and the substrate was then added to initiate the enzyme reaction. The enzyme reaction was carried out at 37 °C for 30 min. Product (PNP) was monitored spectrophotometrically by measuring the absorbance (λ = 400 nm) [19].

## 4. Conclusions

*Alternaria* sp. XZSBG-1 is a prolific producer of anthraquinones. Eleven more compounds have been isolated from this strain, including one new altersoanol and three new alterporriols. Compound **1** is a novel tetrahydroanthraquinone with an epoxy ether bond between C-4a and C-9a. In the primary bioassays, compound **9** showed good inhibitory activity against HCT-116 and HeLa cells and compound **3** showed good inhibitory activity on α-glucosidase. Although these new compounds showed no real activity in our primary bioassay, except **3**, in view of the structural features of these compounds, it is valuable further to study their other biological activities, especially for compound **1**.

## Supplementary Materials

Supplementary materials can be accessed at: http://www.mdpi.com/1420-3049/19/10/16529/s1.

## Supplementary Files

Supplementary File 1

## References

[1] Lin Y.C., Wu X.Y., Deng Z.J., Wang J., Zhou S.N., Vrijmoed L.L.P., Jones E.B.G. (2002). The metabolites of the mangrove fungus Verruculina enalia No. 2606 from a salt lake in the Bahamas. Phytochemistry.

[2] Chen W.Y., Wyk V.B.-E., Vermaak I., Viljoen M.A. (2012). Cape aloes—A review of the phytochemistry, pharmacology and commercialsation of Aloe ferox. Phytochem. Lett..

[3] Shukla V., Joshi G.P., Rawat M.S.M. (2010). Lichens as a potential natural source of bioactive compounds: A review. Pytochem. Rev..

[4] Mishra B.B., Singh D.D., Kishore N., Tiwari V.K., Tripathi V. (2010). Antifungal constituents isolated from the seeds of Aegle marmelos. Phytochemistry.

[5] Marcello L. (2011). Anthraquinones: Analytical techniques as a novel tool to investigate on the triggering of biological targets. Curr. Drug Targets.

[6] Suemitsu R., Horiuchi K., Kubota M., Okamatsu T. (1990). Production of alterporriols, altersolanols and macrosporin by Alternaria porri and A. Solani. Phytochemistry.

[7] Ohnishi K., Suemitsu R., Kubota M., Matano H., Yamada Y. (1991). Biosyntheses of alterporriol D and E by Alternaria porri. Phytochemistry.

[8] Phuwapraisirisa P., Rangsan J., Siripong P., Tip-pyang S. (2009). New antitumour fungal metabolites from Alternaria porri. Nat. Prod. Res..

[9] Debbab A., Aly A.H., Edrada-Ebel R.A., Wray V., Müller W.E.G., Totzke F., Zirrgiebel U., Schächtele C., Kubbutat M.H.G., Lin W.H. (2009). Bioactive metabolites from the endophytic fungus Stemphylium globuliferum isolated from Mentha pulegium. J. Nat. Prod..

[10] Debbab A., Aly A.H., Edrada-Ebel R.A., Wray V., Pretsch A., Pescitelli G., Kurtan T., Proksch P. (2012). New anthracene derivatives- structure elucidation and antimicrobial activity. Eur. J. Org. Chem..

[11] Huang C.H., Pan J.H., Chen B., Yu M., Huang H.B., Zhu X., Lu Y.J., She Z.G., Lin Y.C. (2011). Three bianthraquinone derivatives from the mangrove endophytic fungus *Alternaria* sp. ZJ9–6B from the South China Sea. Mar. Drugs.

[12] Zheng C.J., Shao C.L., Guo Z.Y., Chen J.F., Deng D.S., Yang K.L., Chen Y.Y., Fu X.M., She Z.G., Lin Y.C. (2012). Bioactive hydroanthraquinones and anthraquinones dimmers from a soft coral-derived *Alternaria* sp. fungus. J. Nat. Prod..

[13] Okamura N., Haraguchi H., Hashimoto K., Yagi A. (1993). Altersolanol-related antimicrobial compounds from a strain of *Alternaria solani*. Phytochemistry.

[14] Okamura N., Yagi A., Haraguchi H., Hashimoto K. (1993). Simultaneous high-performance liquid chromatographic determination of altersolanol A, B, C, D, E and F. J. Chromatogr. A.

[15] Höller U., Gloer J.B., Wicklow D.T. (2002). Biologically active polyketide metabolites from an undetermined fungicolous Hyphomycete resembling *Clasdosporium*. J. Nat. Prod..

[16] Kanamaru S., Honma M., Murakami T., Tsushima T., Kudo S., Tanaka K., Nihei K., Nehira T., Hashimoto M. (2012). Absolute stereochemistry of altersolanol A and alterporriols. Chirality.

[17] Huang C.H., Jin H., Song B., Zhu X., Zhao H.X., Cai J.J., Lu Y.J., Chen B., Lin Y.C. (2012). The cytotoxicity and anticancer mechanisms of alterporriol L, a marine bianthraquinone, agaist MCF-7 human breast cancer cells. Appl. Microbiol. Biotechnol..

[18] Chen H., Zhong L.L., Long Y.H., Li J., Wu J.H., Liu L., Chen S.P., Lin Y.C., Li M.F., Zhu X. (2012). Studies on the synthesis of derivatives of marine-derived bostrycin and their structure-activity relationship against tumor cells. Mar. Drugs.

[19] Du Z.Y., Liu R.R., Shao W.Y., Mao X.P., Ma L., Gu L.Q., Huang Z.S., Chan A.S.C. (2006). Alpha-glucosidase inhibition of natural curcuminoids and curcumin analogs. Eur. J. Med. Chem..

[20] Stewart C.N., Via L.E. (1993). A rapid CTAB DNA isolation technique useful for RAPD fingerprinting and other PCR applications. Biotechniques.

