# Supplementary material for: The Anthraquinone Derivatives from the Fungus Alternaria sp. XZSBG-1 from the Saline Lake in Bange, Tibet, China
Source: Molecules. 2014 Oct 14;19(10):16529–42. doi: 10.3390/molecules191016529 (PMC6271467; doi:10.3390/molecules191016529)

Supporting Information

Series of Anthraquinone Derivatives from a Fungus Alternaria sp. XZSBG-1

Bin Chen [1,2], Qiong Shen [1,*], Xun Zhu [3] and Yongcheng Lin [1,*]

[1] School of Chemistry and Chemical Engineering, Sun Yat-sen University, Guangzhou 510275, China; E-Mail: chenyishan@126.com

[2] Tibet Plateau Institute of Biology, Lhasa 850001, China

[3] Zhongshan School of Medicine, Sun Yat-sen University, Guangzhou 510275, China; E-Mail: zhuxun8@mail.sysu.edu.cn

* Author to whom correspondence should be addressed;
E-Mails: cesshq@163.com (Q.S.); ceslyc@mail.sysu.edu.cn (Y.L.);
Tel./Fax: +86-20-8403-9623 (Y.L.).

List of Supporting Information

Table S1. NMR Data of Compound 1 and 2 (DMSO- *d*6), Measured at 400 MHz (^{1}H) and 100 MHz (^{13}C).
Table S2. NMR Data of Compound 3 and 4 (DMSO- *d*6), Measured at 400 MHz (^{1}H) and 100 MHz (^{13}C).
Table S3. NMR Data of Compound 5 (DMSO- *d*6), Measured at 400 MHz (^{1}H) and 100 MHz (^{13}C).
Table S4. NMR Data of Compound 6 (DMSO- *d*6), Measured at 400 MHz (^{1}H) and 100 MHz (^{13}C).
Table S5. NMR Data of Compound 7 (DMSO- *d*6), Measured at 400 MHz (^{1}H) and 100 MHz (^{13}C).
Table S6. NMR Data of Compound 8 (DMSO- *d*6), Measured at 400 MHz (^{1}H) and 100 MHz (^{13}C).
Table S7. NMR Data of Compound 9 (DMSO- *d*6), Measured at 400 MHz (^{1}H) and 100 MHz (^{13}C).
Table S8. NMR Data of Compound 10 (DMSO- *d*6), Measured at 400 MHz (^{1}H) and 100 MHz (^{13}C).
Figure S1. ^{1}H-NMR Spectra of compound 2 (altersolanol O), Measured at 400 MHz (DMSO- *d*6).
Figure S2. ^{13}C-NMR Spectra of compound 2 (altersolanol O), Measured at 100 MHz (DMSO- *d*6).
Figure S3. ^{1}H-^{1}H COSY of compound 2 (altersolanol O), Measured at 400 MHz (DMSO- *d*6).
Figure S4. HSQC of compound 2 (altersolanol O), Measured at 400 MHz (^{1}H) and 100 MHz (^{13}C) (DMSO- *d*6).
Figure S5. HMBC of compound 2 (altersolanol O), Measured at 400 MHz (^{1}H) and 100 MHz (^{13}C) (DMSO- *d*6).
Figure S6. NOESY of compound 2 (altersolanol O), Measured at 400 MHz (^{1}H) and 100 Hz (^{13}C) (DMSO- *d*6).
Figure S7. HR-ESI-TOF-MS spectra of compound 2 (altersolanol O).
Figure S8. CD spectra of compound 2 (altersolanol O)in acetonitrile solution.
Figure S9. ^{1}H-NMR Spectra of compound 2 (alterporriol S), Measured at 400 MHz (DMSO- *d*6).
Figure S10. ^{13}C-NMR Spectra of compound 2 (alterporriol S), Measured at 100 MHz (DMSO- *d*6).
Figure S11. ^{1}H-^{1}H COSY of compound 2 (alterporriol S), Measured at 400 MHz (DMSO- *d*6).
Figure S12. HSQC of compound 2 (alterporriol S), Measured at 400 MHz (^{1}H) and 100 MHz (^{13}C) (DMSO- *d*6).
Figure S13. HMBC of compound 2 (alterporriol S), Measured at 400 MHz (^{1}H) and 100 MHz (^{13}C) (DMSO- *d*6).
Figure S14. NOESY of compound 2 (alterporriol S), Measured at 400 MHz (^{1}H) and 100 MHz (^{13}C) (DMSO- *d*6).
Figure S15. HR-ESI-TOF-MS spectra of compound 2 (alterporriol S).
Figure S16. CD spectra of compound 2 (alterporriol S)in acetonitrile solution.
Figure S17. ^{1}H-NMR Spectra of compound 3 (alterporriol T), Measured at 400 MHz (DMSO- *d*6).
Figure S18. ^{13}C-NMR Spectra of compound 3 (alterporriol T), Measured at 100 MHz (DMSO- *d*6).
Figure S19. ^{1}H-^{1}H COSY of compound 3 (alterporriol T), Measured at 400 MHz (DMSO- *d*6).
Figure S20. HSQC of compound 3 (alterporriol T), Measured at 400 MHz (^{1}H) and 100 MHz (^{13}C) (DMSO- *d*6).
Figure S21. HMBC of compound 3 (alterporriol T), Measured at 400 MHz (^{1}H) and 100 MHz (^{13}C) (DMSO- *d*6).

Figure S22. **NOESY of compound 3 (alterporriol T), Measured at 400 MHz (^{1}H) and 100 MHz (^{13}C) (DMSO- *d*6).**
Figure S23. **HR-ESI-TOF-MS spectra of compound 3 (alterporriol T).**
Figure S24. **CD spectra of Compound 3 (alterporriol T) in acetonitrile solution.**
Figure S25. **^{1}H-NMR Spectra of compound 3 (alterporriol T), Measured at 400 MHz (DMSO- *d*6).**
Figure S26. **^{13}C-NMR Spectra of compound 4 (alterporriol U), Measured at 100 MHz (DMSO- *d*6).**
Figure S27. **^{1}H-^{1}H COSY of compound 4 (alterporriol U), Measured at 400 MHz (DMSO- *d*6).**
Figure S28. **HSQC of compound 4 (alterporriol U), Measured at 400 MHz (^{1}H) and 100 MHz (^{13}C) (DMSO- *d*6).**
Figure S29. **HMBC of compound 4 (alterporriol U), Measured at 400 MHz (^{1}H) and 100 MHz (^{13}C) (DMSO- *d*6).**
Figure S30. **NOESY of compound 4 (alterporriol U), Measured at 400 MHz (^{1}H) and 100 MHz (^{13}C) (DMSO- *d*6).**
Figure S31. **HR-ESI-TOF-MS spectra of compound 4 (alterporriol U).**
Figure S32. **CD spectra of Compound 4 (alterporriol U) in acetonitrile solution.**
Figure S33. **^{1}H-NMR Data of compound 5 (alterporriol E),Measured at 400 MHz (DMSO- *d*6).**
Figure S34. **^{13}C-NMR Data of compound 5 (alterporriol E),Measured at 100 MHz (DMSO- *d*6).**
Figure S35. **DEPT-90 ^{13}C-NMR Data of compound 5 (alterporriol E),Measured at 100 MHz (DMSO- *d*6).**
Figure S36. **DEPT-135 ^{13}C-NMR Data of compound 5 (alterporriol E),Measured at 100 MHz (DMSO- *d*6).**
Figure S37. **^{1}H-^{1}H COSY of compound 5 (alterporriol E),Measured at 400 MHz (DMSO- *d*6).**
Figure S38. **HSQC of compound 5 (alterporriol E), Measured at 400 MHz (^{1}H) and 100 MHz (^{13}C) (DMSO- *d*6).**
Figure S39. **HMBC of compound 5 (alterporriol E), Measured at 400 MHz (^{1}H) and 100 MHz (^{13}C) (DMSO- *d*6).**
Figure S40. **NOESY of compound 5 (alterporriol E), Measured at 400 MHz (^{1}H) and 100 MHz (^{13}C) (DMSO- *d*6).**
Figure S41. **HR-ESI-TOF-MS spectra of compound 5 (alterporriol E).**
Figure S42. **CD spectra of compound 5 (alterporriol E)in acetonitrile solution.**
Figure S43. **^{1}H-NMR Data of compound 6, Measured at 400 MHz (DMSO- *d*6).**
Figure S44. **^{13}C-NMR Data of compound 6, Measured at 100 MHz (DMSO- *d*6).**
Figure S45. **HSQC of compound 6, Measured at 400 MHz (^{1}H) and 100 MHz (^{13}C) (DMSO- *d*6).**
Figure S46. **HMBC of compound 6, Measured at 400 MHz (^{1}H) and 100 MHz (^{13}C) (DMSO- *d*6).**
Figure S47. **NOESY of compound 6, Measured at 400 MHz (^{1}H) and 100 MHz (^{13}C) (DMSO- *d*6).**
Figure S48. **CD spectra of compound 6 in acetonitrile solution.**
Figure S49. **HR-ESI-TOF-MS spectra of compound 6.**
Figure S50. **^{1}H-NMR Spectra of compound 7, Measured at 400 MHz (DMSO- *d*6).**
Figure S51. **^{13}C-NMR Spectra of compound 7, Measured at 100 MHz (DMSO- *d*6).**
Figure S52. **^{1}H-^{1}H COSY of compound 7, Measured at 400 MHz (DMSO- *d*6).**
Figure S53. **HSQC of compound 7, Measured at 400 MHz (^{1}H) and 100 MHz (^{13}C) (DMSO- *d*6).**
Figure S54. **HMBC of compound 7, Measured at 400 MHz (^{1}H) and 100 MHz (^{13}C) (DMSO- *d*6).**
Figure S55. **NOESY of compound 7, Measured at 400 MHz (^{1}H) and 100 MHz (^{13}C) (DMSO- *d*6).**
Figure S56. **HR-ESI-TOF-MS spectra of compound 7.**
Figure S57. **CD spectra of compound 7 in acetonitrile solution.**
Figure S58. **^{1}H-NMR Spectra of compound 8, Measured at 400 MHz (DMSO- *d*6).**
Figure S59. **^{13}C-NMR Spectra of compound 8, Measured at 100 MHz (DMSO- *d*6).**
Figure S60. **^{1}H-^{1}H COSY of compound 8, Measured at 400 MHz (DMSO- *d*6).**
Figure S61. **HSQC of compound 8, Measured at 400 MHz (^{1}H) and 100 MHz (^{13}C) (DMSO- *d*6).**
Figure S62. **HMBC of compound 8, Measured at 400 MHz (^{1}H) and 100 MHz (^{13}C) (DMSO- *d*6).**
Figure S63. **NOESY of compound 8, Measured at 400 MHz (^{1}H) and 100 MHz (^{13}C) (DMSO- *d*6).**
Figure S64. **HR-ESI-TOF-MS spectra of compound 8.**
Figure S65. **^{1}H-NMR Spectra of compound 9, Measured at 400 MHz (DMSO- *d*6).**
Figure S66. **^{13}C-NMR Spectra of compound 9, Measured at 100 MHz (DMSO- *d*6).**
Figure S67. **DEPT-90 ^{13}C-NMR Data of compound 9, Measured at 100 MHz (DMSO- *d*6).**
Figure S68. **DEPT-135 ^{13}C-NMR Data of compound 9, Measured at 100 MHz (DMSO- *d*6).**

Figure S69. ^{1}H-^{1}H COSY of compound 9, Measured at 400 MHz (DMSO- *d*6).
Figure S70. HSQC of compound 9, Measured at 400 MHz (^{1}H) and 100 MHz (^{13}C) (DMSO- *d*6).
Figure S71. HMBC of compound 9, Measured at 400 MHz (^{1}H) and 100 MHz (^{13}C) (DMSO- *d*6).
Figure S72. ESI -MS spectra of compound 9.
Figure S73. ^{1}H-NMR Spectra of compound 10, Measured at 400 MHz (DMSO- *d*6).
Figure S74. ^{13}C-NMR Spectra of compound 10, Measured at 100 MHz (DMSO- *d*6).
Figure S75. DEPT-135 ^{13}C-NMR Data of compound 10, Measured at 100 MHz (DMSO- *d*6).
Figure S76. ^{1}H-^{1}H COSY of compound 10, Measured at 400 MHz (DMSO- *d*6).
Figure S77. HSQC of compound 10, Measured at 400 MHz (^{1}H) and 100 MHz (^{13}C) (DMSO- *d*6).
Figure S78. HMBC of compound 10, Measured at 400 MHz (^{1}H) and 100 MHz (^{13}C) (DMSO- *d*6).
Figure S79. ESI -MS spectra of compound 10.
Figure S80. ^{1}H-^{1}H COSY of compound 11, Measured at 400 MHz (DMSO- *d*6).
Figure S81. EI -MS spectra of compound 11.

Table S1. NMR data of compound 1 and 2 (DMSO- *d*6), measured at 400 MHz (^{1}H) and 100 MHz (^{13}C).

Actom	2			3		
	δ_C, (ppm)	δ_H (ppm) (mult., J in Hz)	HMBC	δ_C, (ppm)	δ_H (ppm) (mult., J in Hz)	HMBC
1	67.28	4.42 (d,8.1)	C-2	67.78	4.38 (d,4.32)	C-2,3,4a, 9,9a, 11
2	71.84	3.27 (d,8.1)	C-1	71.69		
3	74.14			69.59	3.77 (dd,15.5,7.8)	C-4
4	67.64	4.43 (s)	C-2, 3, 10,11	42.7	3.82 (dd,1.3,4.8)	C-3,4’ ,4a,9a
4a	68.37			149.43		
5	106.19	6.91 (d,2.48)	C-6, 7,8a, 10	163.04		
6	165.49			105.49	6.80 (d,2.5)	C-5,7, 8,10a
7	106.98	6.81 (d,2.48)	C-5,6,8,8a	164.89		
8	162.11			105.63	7.05 (d,2.5)	C-6,7, 9,10a
8a	110.0			133.69		
9	193.61			183.08		
9a	67.16			140.62		
10	191.12			188.59		
10a	134.46			109.91		
11	21.78	1.12 (s)	C-2,3, 4	22.22	1.13 (s)	C-1,2,3
12	56.43	3.86 (s)	C-6	56.08	3.91 (s)	C-7
1-OH					5.12 (d,4.32)	C-1,2, 9a
2-OH					4.14 (s)	C-1
3-OH					4.09 (d,7.8)	C-3,4
5-OH					12.26 (s)	C-5, 6, 10a
8-OH		11.16 (s)				

Table S2. NMR data of compound 3 and 4 (DMSO- *d*6), measured at 400 MHz (^{1}H) and 100 MHz (^{13}C).

Actom	4				5			
	δ_C, (ppm)	δ_H (ppm) (mult., J in Hz)	HMBC	NOE	δ_C, (ppm)	δ_H (ppm) (mult., J in Hz)	HMBC	NOE
1	68.39	4.47 (dd,5.78,7.06)	C-2,9a	H- 4-OH	68.45	4.48 (dd,5.60,6.87)	C-2,4a,9a,9	H-4
2	73.78	3.57 (dd,7.06,7.06)	C-1	H-11	73.74	3.55 (dd,6.87,6.87)	C-1	H-11
3	72.83				72.87			
4	68.22	4.03 (d,6.93)	C-2,3,4a, 10, 11	H-11,1-OH	68.27	4.05 (d,6.77)	C-2,9a, 0,4a,11	H-1,11
4a	143.31				143.42			
5	122.86				122.59			
6	164.28				164.33			
7	103.77	6.93 (s)	C-5,8, 8a		103.77	6.92 (s)	C-6,8, 8a ,9	H-12’
8	163.63				163.73			
8a	109.27				109.3			
9	188.77				188.79			
9a	142.78				142.61			
10	184.09				183.88			
10a	128.86				129.02			
11	22.18	1.13 (s)	C-3,4	H-2,4,1-OH	22.22	1.13 (s)	C-3,4	H-2,4
12	56.74	3.70 (s)	C-6	H-7	56.71	3.69 (s)	C-6	H-7
1-OH		5.64 (d,7.06)	C-1, 3,9a	H-4		4.98 (d,5.6)	C-1,2, 9a	H-4-OH
2-OH		4.36 (s)	C-2,4			4.81 (d,6.87)	C-1,2,3	H-4-OH
3-OH		4.85 (d,6.93)	C-1			4.38 (s)	C-2,4, 11	H-1-OH
4-OH		5.04 (d,5.78)	C-2,4,4a	H-1		5.44 (d,6.77)	C-3,4, 4a	H- 2-OH
8-OH		13.04 (s)	C-7,8,8a			13.04 (s)	C-7,8, 8a	
1’	110.44	7.56 (s)	C- 2’, 3’, 9’, 9a’		110.42	7.55 (s)	C-2’,3’, 9’,9a’,10’	H-2’-OH
2’	161.19				161.24			
3’	125.25				125.27			
4’	130.18	7.70 (d,0.56)	C-3’,4a’,10’, 11’	H-11’	130.33	7.70 (d,0.69)	C-2’,4a’, 9’,10’,11’	H-11’
4a’	132.39				132.38			

Table S2. *Cont.*

Actom	4				5			
	δ$_C$, (ppm)	δ$_H$ (ppm) (mult., J in Hz)	HMBC	NOE	δ$_C$, (ppm)	δ$_H$ (ppm) (mult., J in Hz)	HMBC	NOE
5’	121.81				123.12			
6’	164.18				165.25			
7’	104.01	6.94 (s)	C- 5’,8’, 8a’,9’		103.54	6.94 (s)	C- 6,8’, 8a’ ,9’	H-12,8’-OH
8’	164.85				165.23			
8a’	109.98				109.92			
9’	186.7				186.61			
9a’	132.2				132.2			
10’	181.1				180.97			
10a’	131.48				130.43			
11’	16.03	2.20 (s)	C-2’,4’	H-4’	16.09	2.19 (s)	C-2’,3’,4’	H-4’
12’	56.65	3.68 (s)	C-6’	H-7’	56.83	3.72 (s)	C-6’	H-7’
2’-OH		11.07 (brs)				11.03 (brs)		H-1’
8’-OH		13.60 (s)	C-7’ ,8’,8a’			13.66 (s)	C-7’,8’,8a’	H-7’

Table S3. NMR data of compound 5 (DMSO- *d*6), measured at 400 MHz (^{1}H) and 100 MHz (^{13}C).

position	1			
	**δ_C, mult**	**δ_H (J in HZ)**	**HMBC**	**NOE**
1	68.4	4.48 (dd,5.63,6.96)	C-2,4a ,9a	
2	73.8	3.59 (dd,6.85,6.96)	C-1	H-11
3	72.9			
4	68.2	4.10 (d,7.05)	C-3, 4a,10,9a, 11	H-11
4a	142.8			
5	129.8			
6	163.5			
7	163.6			
8	104.2	6.91 (s)	C-10a,7,8a,9	H-12
8a	121.4			
9	188.8			
9a	143.4			
10	184.1			
10a	109.3			
11	22.2	1.16 (s)	C-3,4	H-2,4
12	56.7	3.68 (s)	C-8	
1-OH		4.98 (d,5.63)	C-1, 2 ,9a	
2-OH		4.80 (d,6.85)	C-1,3	
3-OH		4.37 (s)	C-3,4,11	1-OH
4-OH		5.63 (d,7.05)	C-3,4,4a	
6-OH		6.91 (s)		
8-OH				

Table S4. NMR data of compound 6 (DMSO- *d*6), measured at 400 MHz (^{1}H) and 100 MHz (^{13}C).

no	δ_C, mult	δ_H (J in Hz)	H-H-COSY	HMBC	NOESY
1	68.49	4.48 (dd,4.6,6.26,1H)	H-2,4	C-2, 4a ,10,10a	H-4
2	73.83	3.57 (m,1H)	H-1	C-1	
3	72.94				
4	68.22	4.08 (d,6.84,1H)	H-1	C-3, 4a, 5,10a, 11	H-1
4a	142.89				
5	183.82				
5a	128.9				
6	109.28				
7	164.73				
8	103.79	6.93 (s,1H)		C-6,9,9a, 10	H-12
9	164				
9a	122.45				
10	188.76				
10a	143.36				
11	22.25	1.15 (s,3H)		C-3,4	H-1,2, 4,4-OH
12	56.85	3.70 (s,3H)		C-7,8	H-8

Table S4. *Cont.*

no	δC, mult	δH (J in Hz)	H-H-COSY	HMBC	NOESY
	1-OH	5.04 (d,4.6,1H)		C-1,2, 10a	H-1,2-OH
	2-OH	4.84 (s,1H)			H-1
	3-OH	4.41 (s,1H)		C-3,4	
	4-OH	5.63 (d,6.84,1H)		C-3,4,4a	H-1,4, 1-OH ,2-OH
	9-OH	13.07 (br,1H)			H-1,1-OH,2-OH, 4OH

Table S5. NMR data of compound 7 (DMSO- *d*6), measured at 400 MHz (^{1}H) and 100 MHz (^{13}C).

No.	δC, mult	δH (J in HZ)	H-H-COSY	HMBC	NOESY
1	68.39 (CH)	4.49 (dd,5.40,7.11,1H)	H-2, 1-OH	C-2,4a,9a,9	
2	73.72 (CH)	3.58 (dd,6.66,6.66,1H)	H-1,2-OH	C-1	H-11
3	72.92 (C)				
4	68.2 (CH)	4.06 (s,1H)		C- 3,4a,9a, 10,11	H-11
4a	143.4 (C)				
5	122.89 (C)				
6	164.18 (C)				
7	103.95 (CH)	6.94 (s,1H)		**C-5, 8, 8a,9**	H-12
8	163.84 (C)				
8a	109.65 (C)				
9	189.04 (C)				
9a	142.69 (C)				
10	184.26 (C)				
10a	134.54 (C)				
11	22.21 (CH3)	1.13 (s,3H)		C-3,4	H-2,4
12	56.71 (CH3)	3.70 (s,3H)		**C-6**	H-7
1-OH		4.98 (d,5.40)	H-1	C-1,2,4a	
2-OH		4.79 (d,6.78)	H-2	C-1	H-4-OH
3-OH		4.33 (s,1H)		C-2,3,4	
4-OH		5.69 (br)			H-1-OH,2-OH, 3-OH
8-OH		13.06 (s)		C- 6,7	
1’	125.81 (C)				
2’	159.19				
3’	131.56 (C)				
4’	129.47 (CH)	8.03 (d,0.66,1H)	H-11’	C-2’,4a’,9’,9a’,10’,11’	
4a’	125.43 (C)				
5’	106.44 (CH)	7.17 (d,2.57)	H-7’	C-6’,7,8a’,10	H-12’
6’	165.58 (C)				
7’	105.99 (CH)	6.75 (d,2.57)	H-5’	C-5’,8’,8a’	H-12’
8’	164.41 (C)				
8a’	110.27 (C)				
9’	187.59 (C)				
9a’	130.92 (C)				
10’	180.71 (C)				
11’	17.15 (CH3)	2.31 (s)		C-2’,3’,4’,4a’	H-4’
12’	56.2 (CH3)	3.90 (s)		C-6’	H-5’,7’
8’-OH		12.54 (s)		C-7’,8’8a’	

Table S6. NMR data of compound 8 (DMSO- *d*6), measured at 400 MHz (^{1}H) and 100 MHz (^{13}C).

No.	δ_C, mult	H-H-COSY	δ_H (J in HZ)	HMBC	NOESY
1	68.4 (CH)	H-2	4.49 (dd,6.92,5.00,1H)	C-2,4a	H-4
2	73.81 (CH)	H-1	3.59 (dd,6.40,6.40,1H)	C-1	H-4
3	72.88 (C)				
4	68.26 (CH)		4.05 (d,6.00,1H)	C-2,4a, 10,11	H-1
4a	143.39 (C)				
5	122.92 (C)				
6	164.21 (C)				
7	104.06 (CH)		6.92 (s,1H)	C-5,8, 8a,9	H-12
8	163.67 (C)				
8a	109.3 (C)				
9	188.79 (C)				
9a	142.8 (C)				
10	184.15 (C)				
10a	128.87 (C)				
11	22.23 (CH3)		1.13 (s,1H)	C- 3,4	H-1,2,4
12	56.8 (OCH3)		3.68 (s,1H)	C-7	H-7
1-OH			4.98 (d,5.00,1H)	C-1 (w),2 (w)	H-3-OH,4-OH
2-OH			4.33 (s,1H)	C-1 (w)	H-4-OH
3-OH			4.80 (d,6.40,1H)		
4-OH			5.60 (d,6.92,1H)	C-4a (w)	H- 1-OH ,2-OH
8-OH			13.02 (s,1H)		
1’	110.48 (CH)		7.56 (s,1H)	C-2’,3’9’,9a’,10’ (w),11’ (w)	
2’	161.3 (C)				
3’	125.24 (C)				
4’	130.24 (CH)	H-11’	7.69 (d,0.72,1H)	C-2’,4a’,9’ (w),10’, 11’	H-11’
4a’	132.43 (C)				
5’	121.86 (C)				
6	164.31 (C)				
7’	103.83 (CH)		6.93 (s,1H)	C-5’, 8’,8a’, 9’	H-12’
8’	164.9 (C)				
8a’	110.01 (C)				
9’	186.75 (C)				
9a’	132.27 (C)				
10’	181.15 (C)				
10a’	131.5 (C)				
11’	16.11 (CH3)	H- 4’	2.19 (s,1H)	C-2’,4’	
12’	56.7 (OCH3)		3.70 (s,1H)	C-3’,7’ (w)	H-7’
8’-OH			13.58 (s,1H)	C-7’,7’,8a’	

Table S7. NMR data of compound 9 (DMSO- *d*6), measured at 400 MHz (^{1}H) and 100 MHz (^{13}C).

No.	δ_C, mult	δ_H (J in HZ)	H-H-COSY	HMBC
1	188.21 (C)			
2	183 (C)			
3	165.72 (C)			
4	163.45 (C)			
5	144.01 (C)			
6	143.04 (C)			
7	133.58 (C)			
8	109.23 (C)			
9	107.05 (CH)	7.01 (d,2.5,1H)	H-10,14	C-2,3,7 (w),8, 10
10	105.7 (CH)	6.76 (d,2.5,1H)	H-9,14	C-3,4,8,9
11	72.1 (C)			
12	69.38 (CH)	4.33 (s,1H)	H-15,16	C2,5,11,13,16-
13	66.91 (CH)	3.75 (dd,5.90,9.60,1H)	H-15	C-15
14	56.37 (OCH3)	3.88 (s,1H)	H-9,10	C-3
15	29.06 (CH2)	2.34 (dd,9.60,19.45,1H)	H-12,13,15	C-2 (w),5, 12
		2.79 (dd,5.90,19.45,1H)	H-13,15	C-1,6,11,12
16	21.93 (CH3)	1.28 (s,1H)	H-12,16	C-11,12,13
	4-OH	12.15 (s,1H)		C-4,8,10

Table S8. NMR data of compound 10 (DMSO- *d*6), measured at 400 MHz (^{1}H) and 100 MHz (^{13}C).

No.	δ_C, mult	δ_H (J in HZ)	H-H-COSY	HMBC	NOESY
1	68.8	4.49 (dd 6.89,6.89)	H-2	C-2,4a,9,9a	H-11
2	74.1	3.64 (dd 6.06,6.06)	H-1	C-1	H-1-OH,11
3	73.2				
4	68.7	4.32 (d 5.10)	H-5.72-OH	C-4a	H-11
4a	142.3				
5	106.9	7.03 (d 2.5)	H-7	C-7,8a,10	H-12
6	165.8				
7	106.2	6.84 (d 2.5)	H-5	C-5,6 ,8,8a	H-12
8	163.4				
8a	109.8				
9	183.9				
9a	144.7				
10	188.7				
10a	133.6				
11	22.5	1.24 (s)	H-2	C-3,4	H-1,2,2-OH,4,4-OH
12	56.5	3.91 (s)		C-6	H-5,7
1-OH		5.04 (d 5.18)			H-4-OH
2-OH		4.87 (d 6.41)			H-1-OH
3-OH		4.45 (d 5.10)			H-4-OH
4-OH		5.67 (d 6.08)	H-4		H-1,2
8-OH		12.16 (br)			

Figure S1. ^{1}H-NMR spectra of compound 1 (altersolanol O), measured at 400 MHz (DMSO- *d*6).

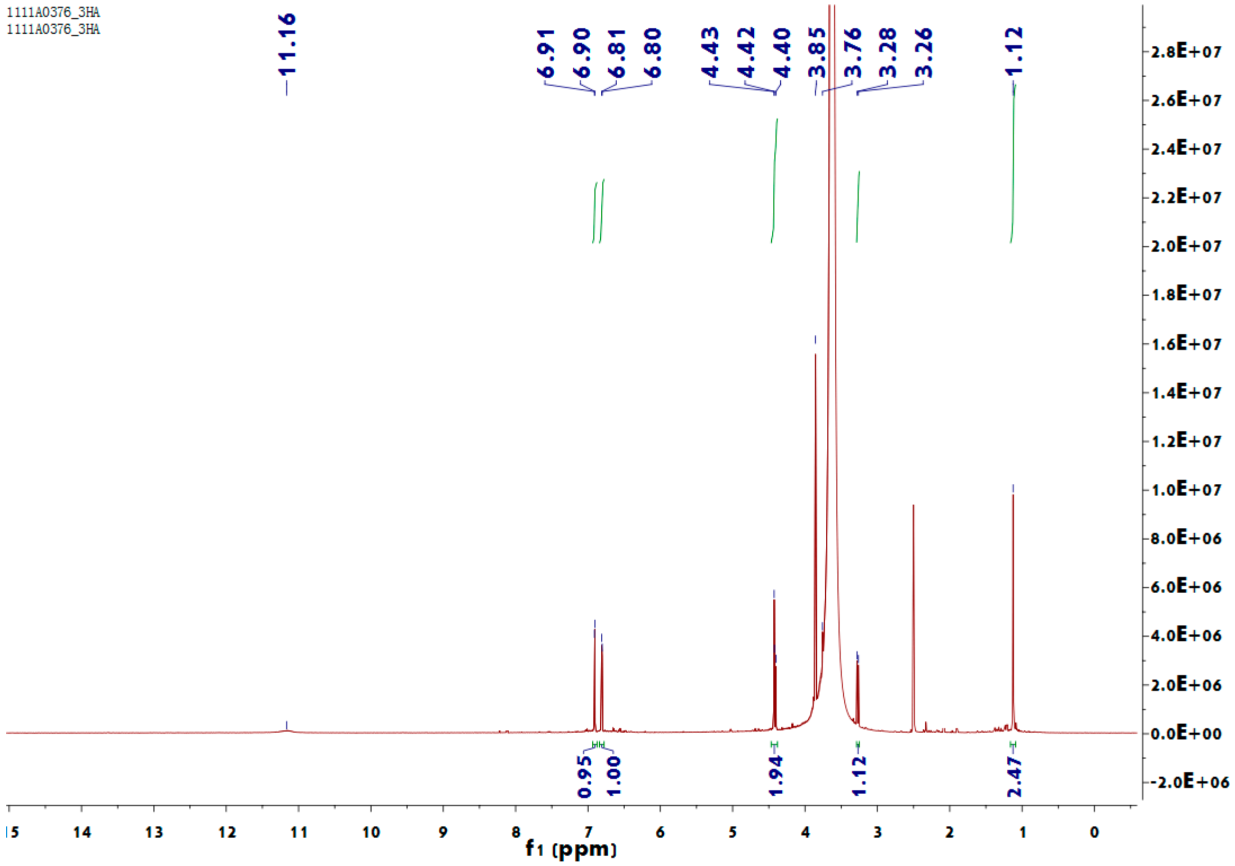

Figure S2. ^{13}C-NMR spectra of compound 1 (altersolanol O), measured at 100 MHz (DMSO- *d*6).

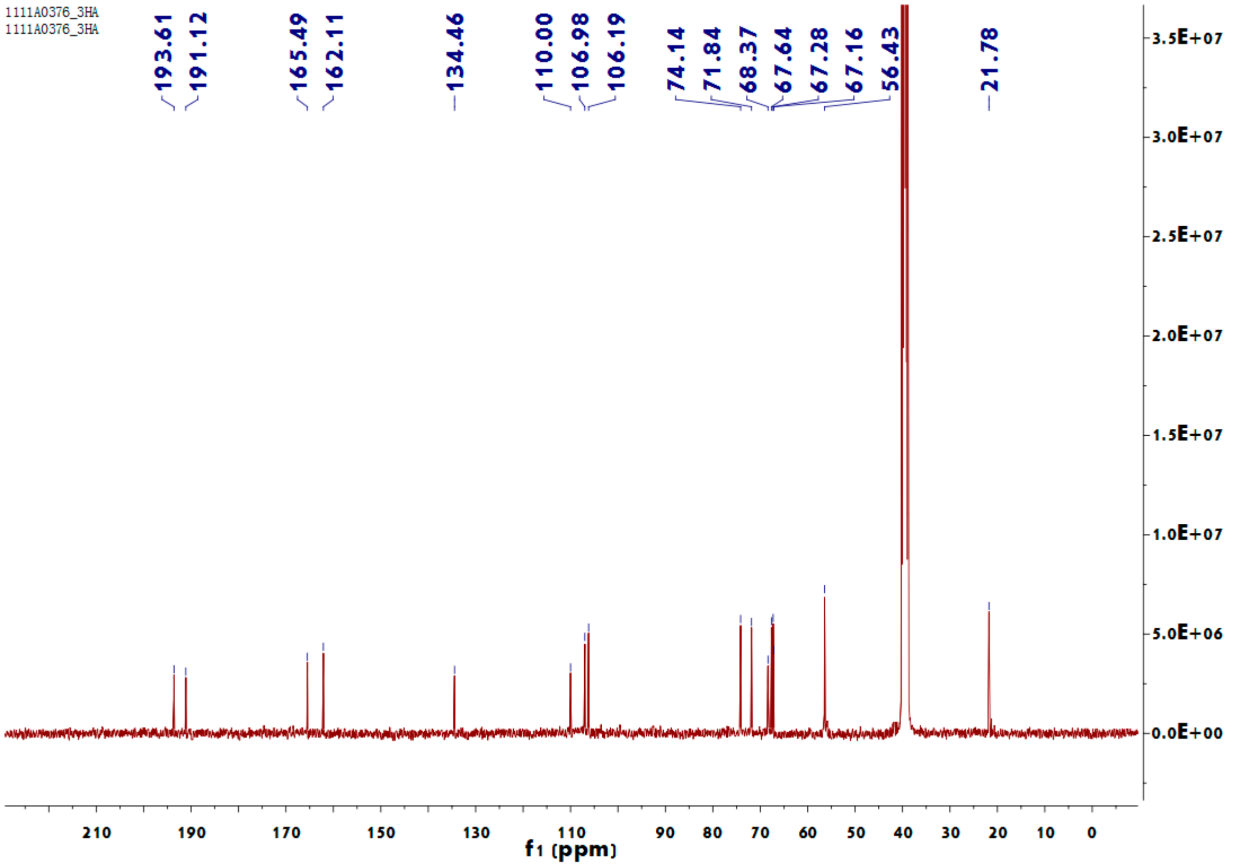

Figure S3. ${}^{1}H$-${}^{1}H$ COSY of compound 1 (altersolanol O), measured at 400 MHz (DMSO- *d*6).

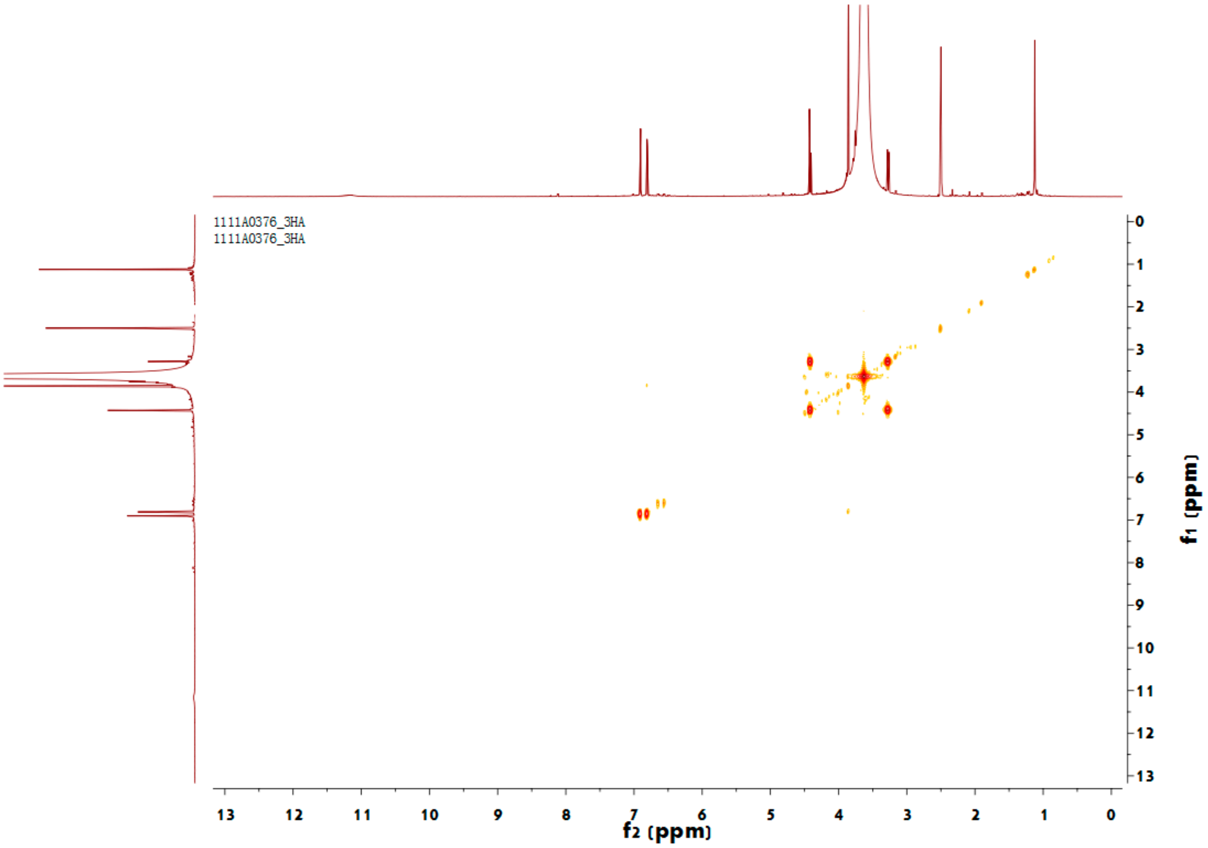

Figure S4. HSQC of compound 1 (altersolanol O), measured at 400 MHz (${}^{1}H$) and 100 MHz (${}^{13}C$) (DMSO- *d*6).

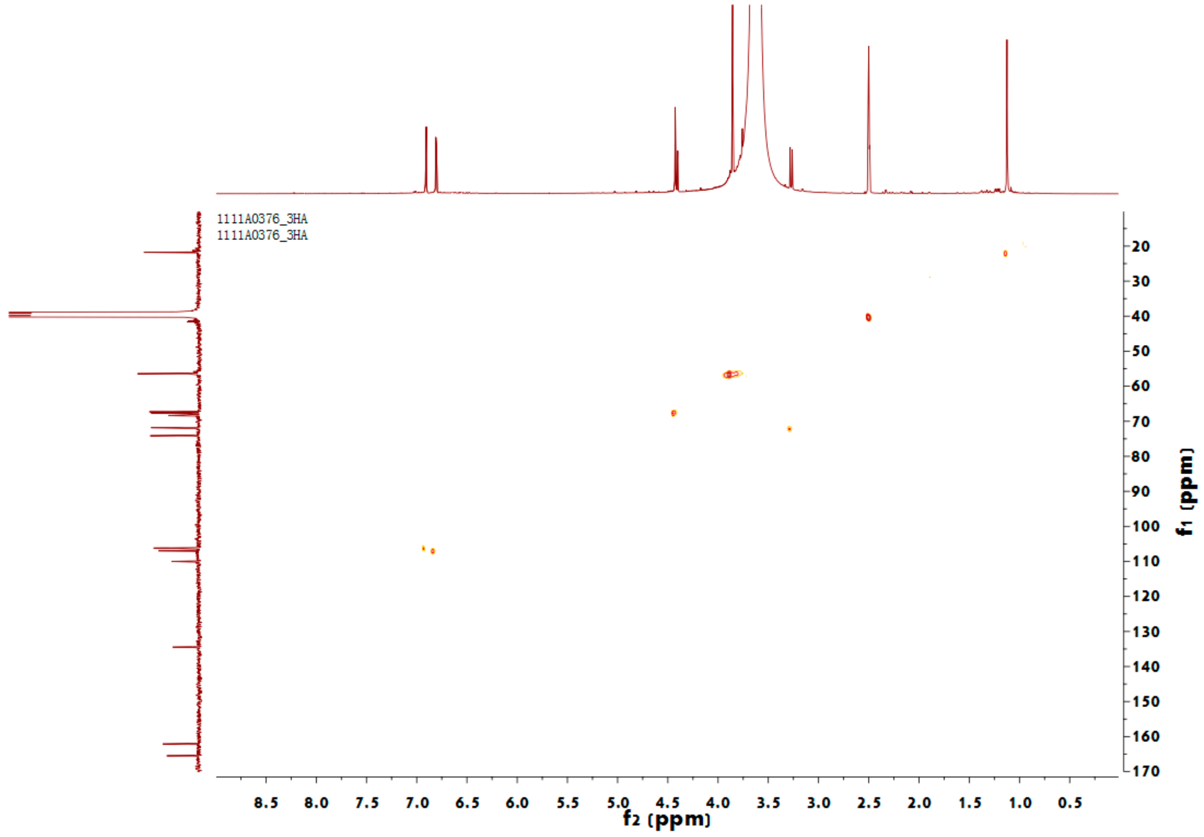

Figure S5. HMBC of compound 1 (altersolanol O), measured at 400 MHz (^{1}H) and 100 MHz (^{13}C) (DMSO- *d*6).

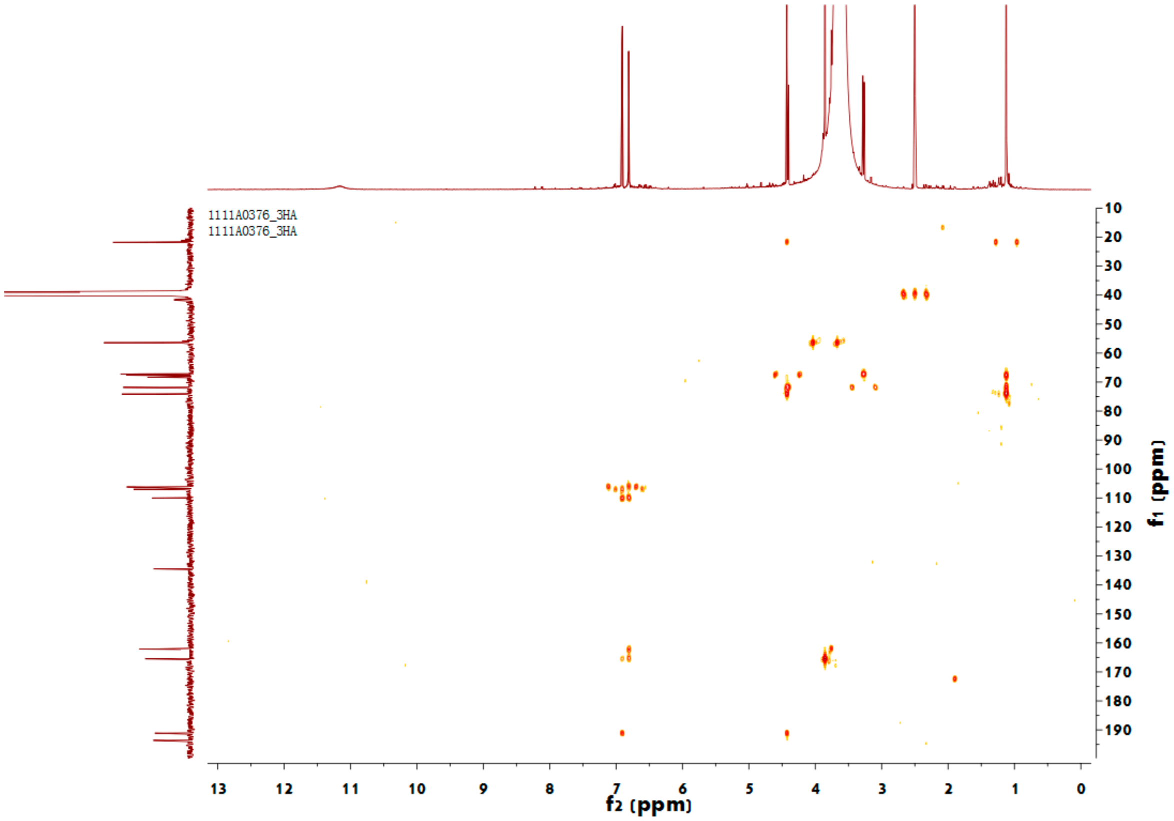

Figure S6. NOESY of compound 1 (altersolanol O), measured at 400 MHz (^{1}H) and 100 MHz (^{13}C) (DMSO- *d*6).

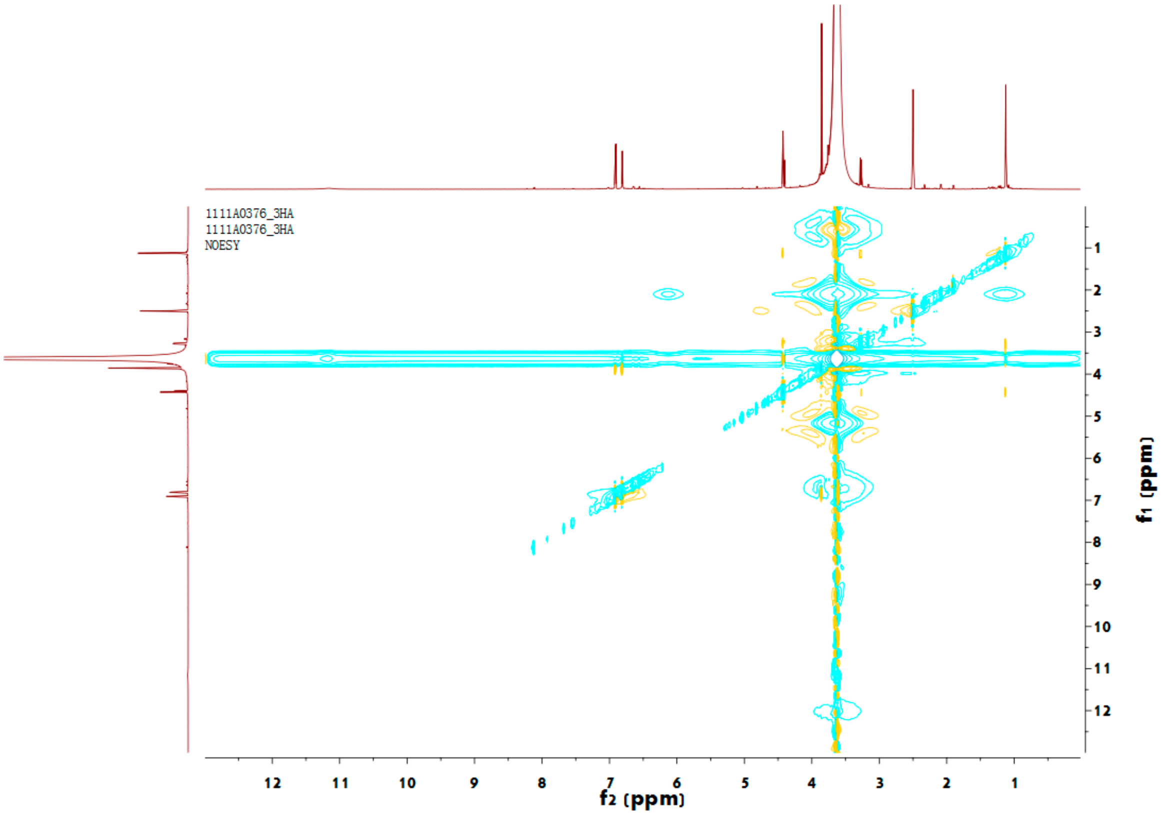

Figure S7. HR-ESI-TOF-MS spectra of compound 1 (altersolanol O).

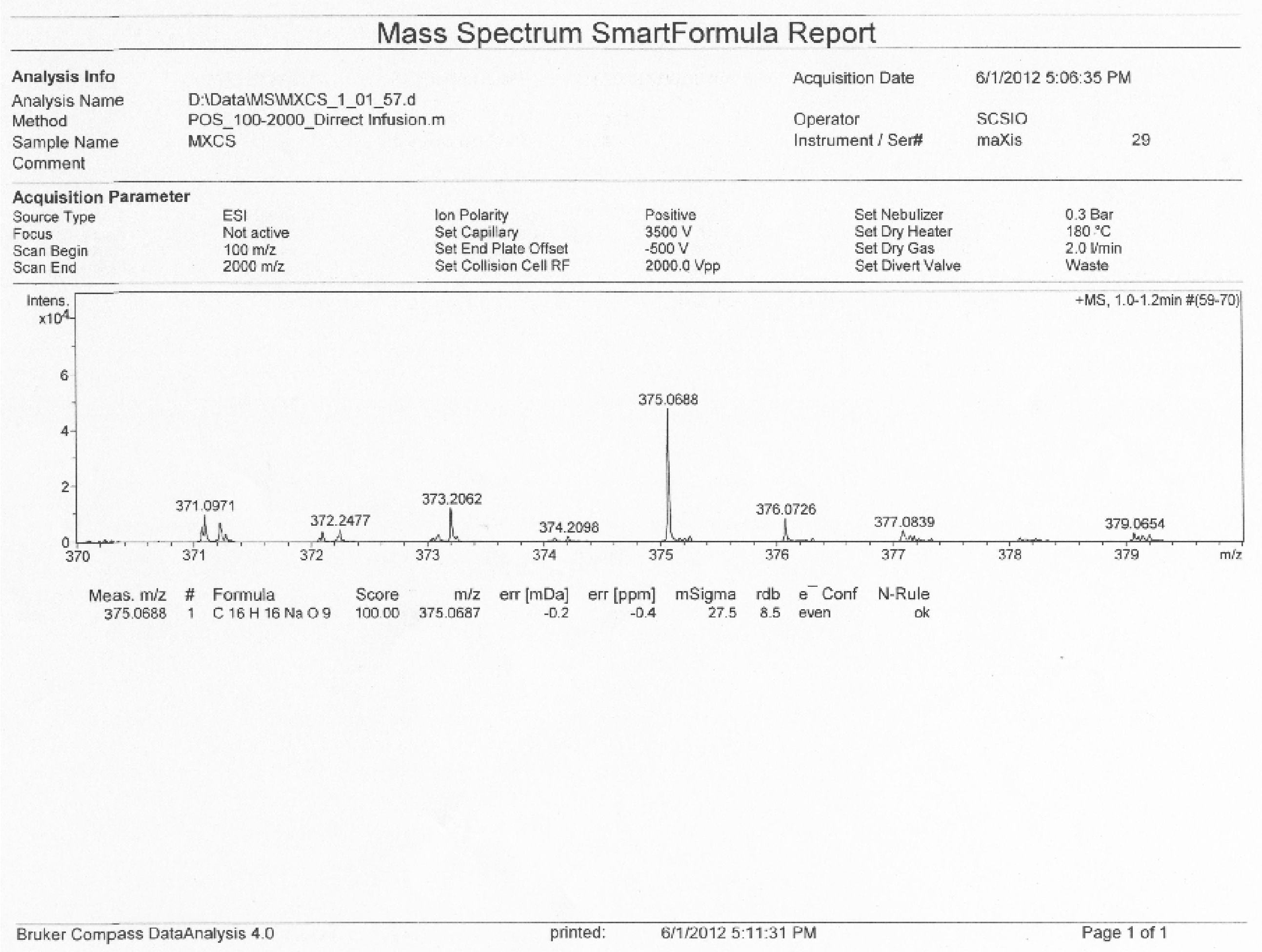

Figure S8. CD spectra of compound 1 (altersolanol O) in acetonitrile solution.

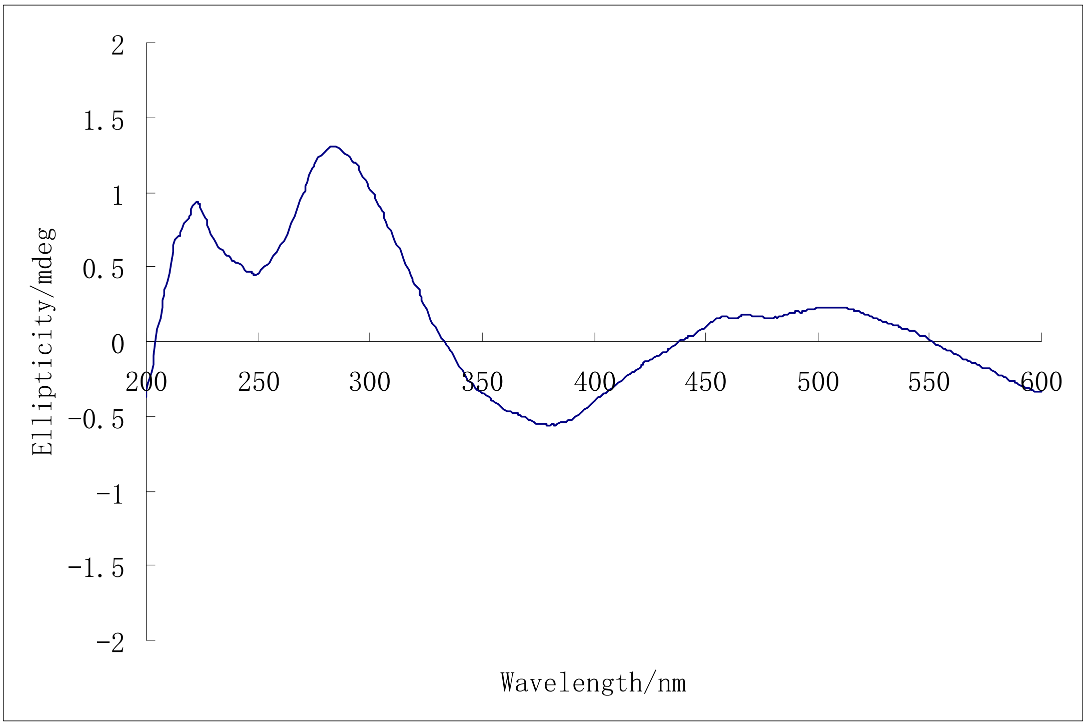

Figure S9. ^{1}H-NMR spectra of compound 2 (alterporriol S), measured at 400 MHz (DMSO- *d*6).

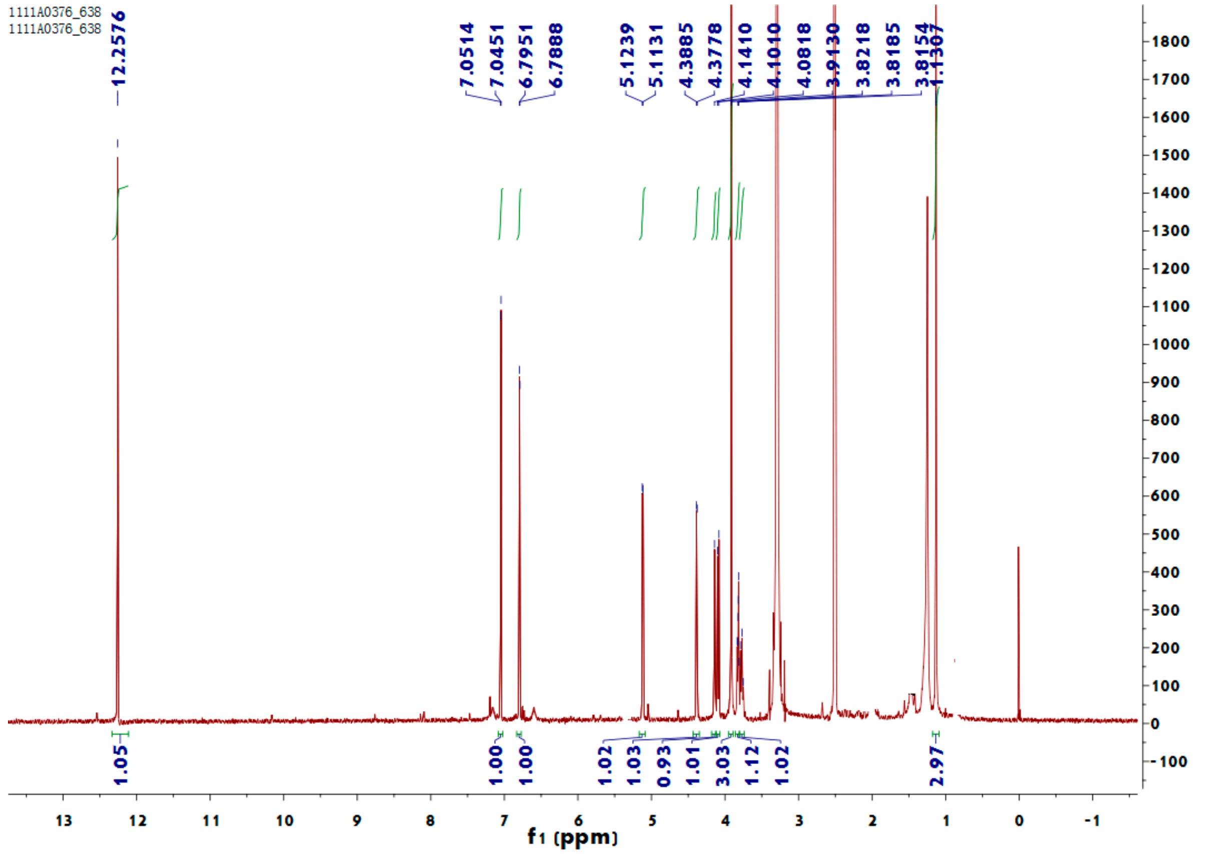

Figure S10. ^{13}C-NMR spectra of compound 2 (alterporriol S), measured at 100 MHz (DMSO- *d*6).

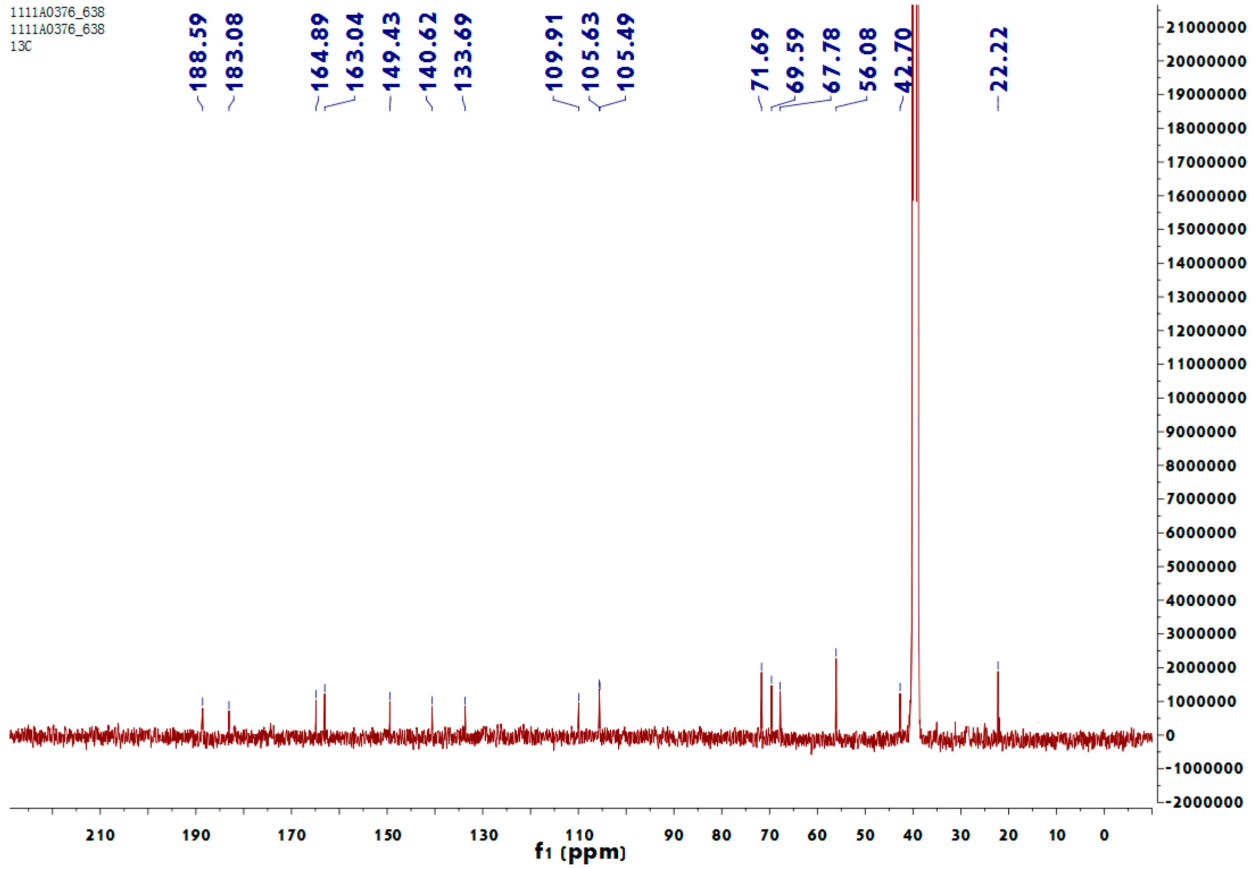

Figure S11. ^{1}H-^{1}H COSY of compound 2 (alterporriol S), measured at 400 MHz (DMSO- *d*6).

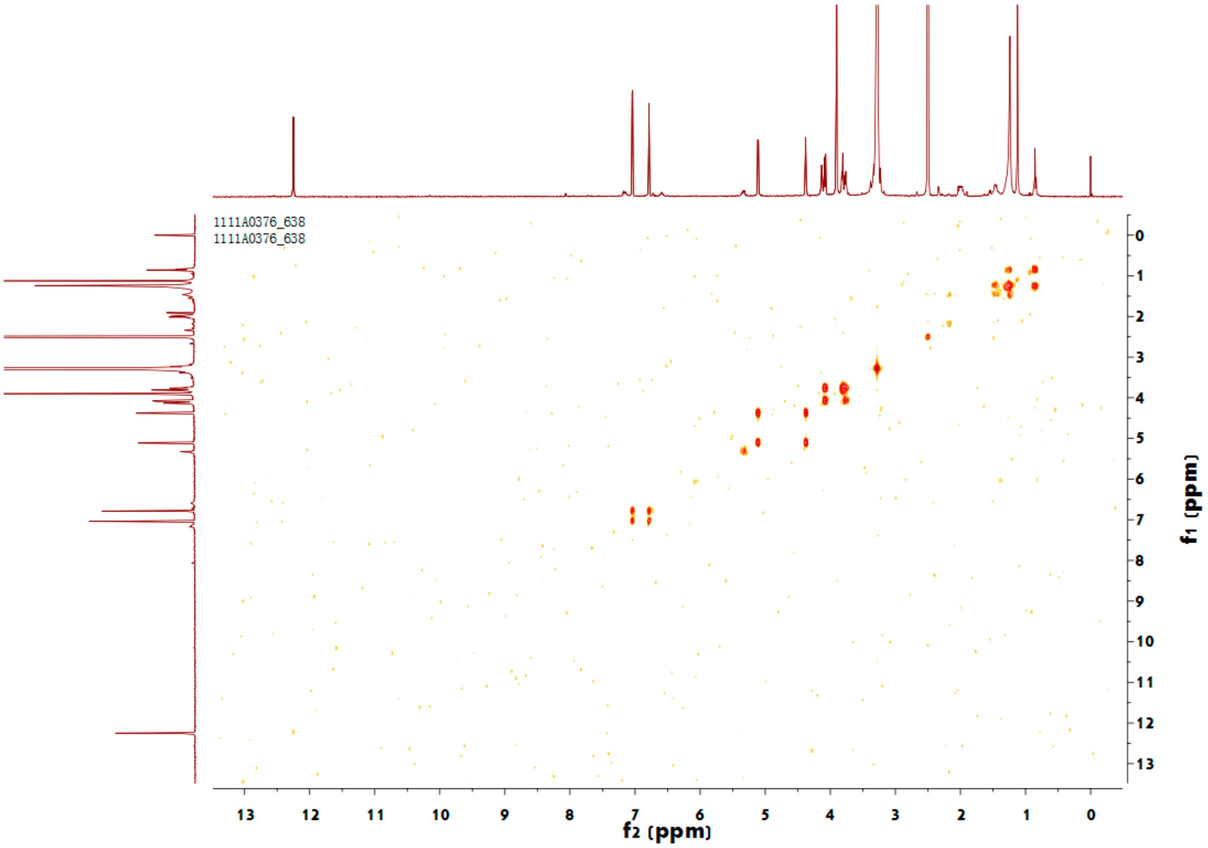

Figure S12. HSQC of compound 2 (alterporriol S), measured at 400 MHz (^{1}H) and 100 MHz (^{13}C) (DMSO- *d*6).

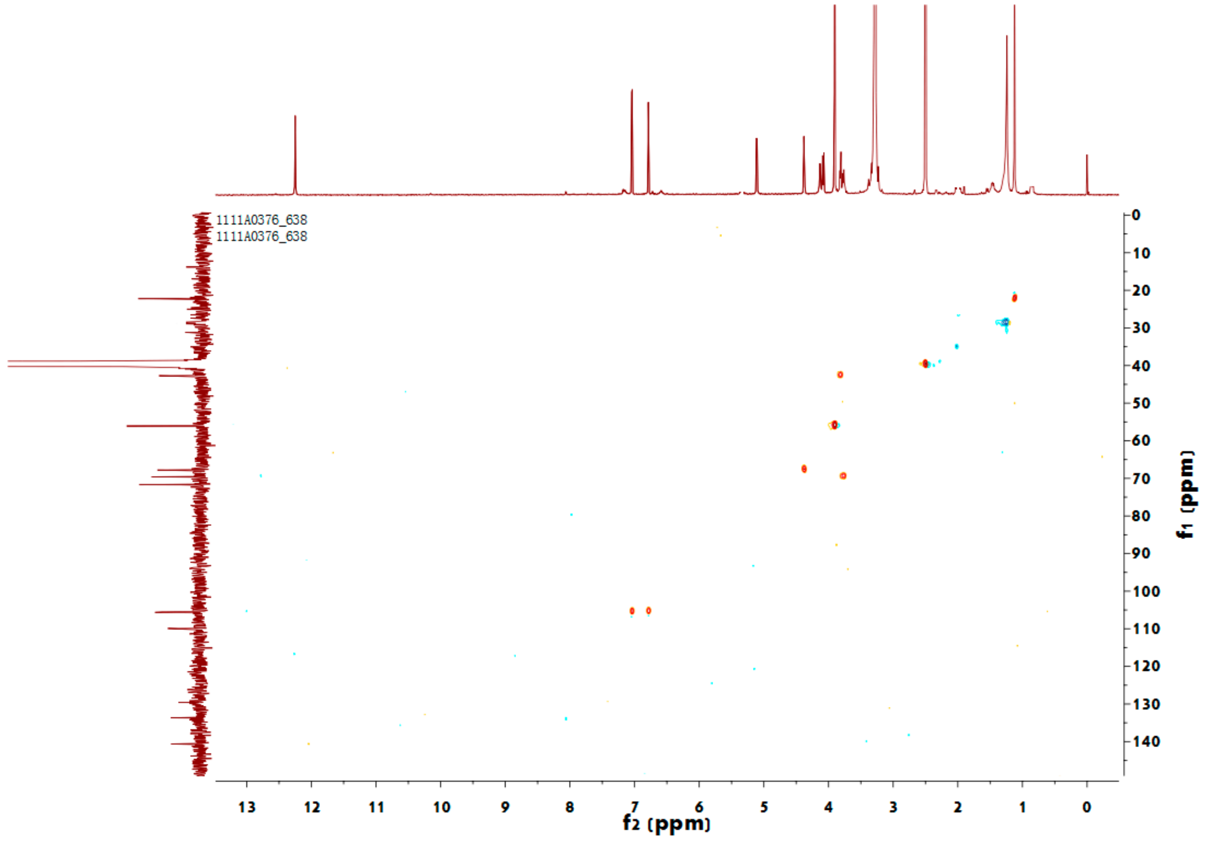

Figure S13. HMBC of compound 2 (alterporriol S), measured at 400 MHz (^{1}H) and 100 MHz (^{13}C) (DMSO- *d*6).

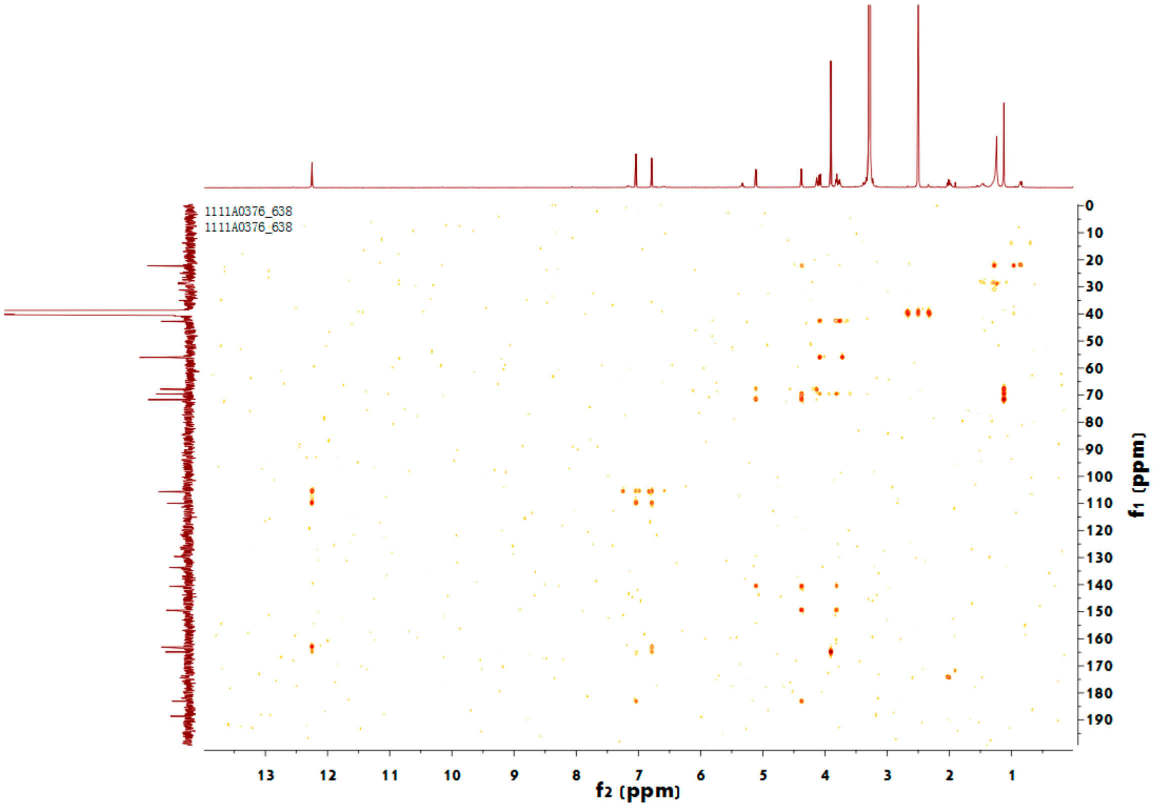

Figure S14. NOESY of compound 2 (alterporriol S), measured at 400 MHz (^{1}H) and 100 MHz (^{13}C) (DMSO- *d*6).

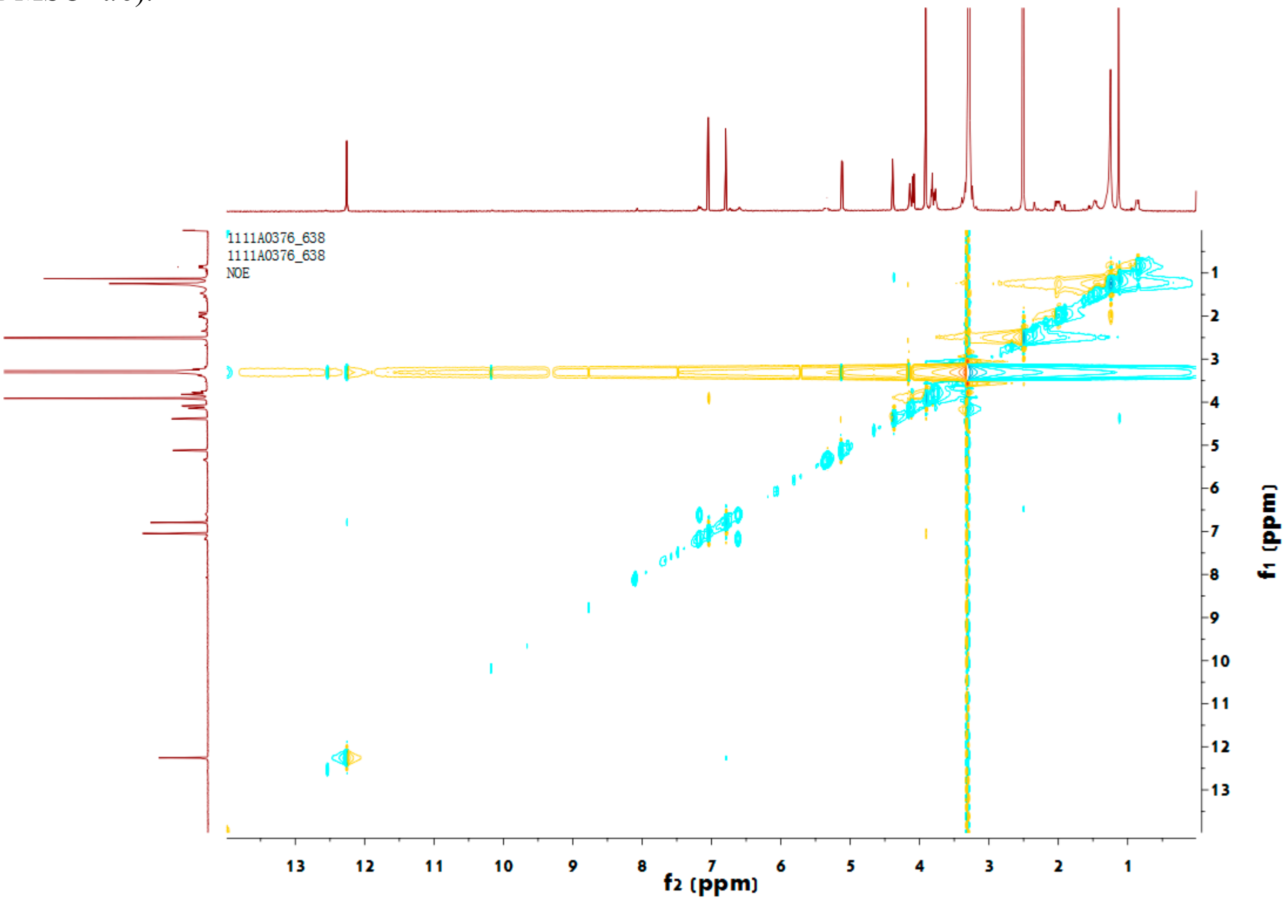

Figure S15. HR-ESI-TOF-MS spectra of compound 2 (alterporriol S).

Formula Predictor Report - 638.lcd

Page 1 of 1

Data File: F:\wang jun\xl\638.lcd

Elmt	Val.	Min	Max	Elmt	Val.	Min	Max	Elmt	Val.	Min	Max	Elmt	Val.	Min	Max	Use Adduct
H	1	0	35	N	3	0	0	P	3	0	0	Br	1	0	0	H
B	3	0	0	O	2	0	15	S	2	0	0	I	3	0	0	
C	4	0	35	F	1	0	0	Cl	1	0	0	Pt	2	0	0	

Error Margin (ppm): 200
HC Ratio: unlimited
Max Isotopes: all
MSn Iso RI (%): 90.00

DBE Range: 0.0 - 3000.0
Apply N Rule: yes
Isotope RI (%): 1.00
MSn Logic Mode: AND

Electron Ions: both
Use MSn Info: yes
Isotope Res: 10000
Max Results: 500

Event#: 2 MS(E-) Ret. Time : 0.280 -> 0.280 - 1.333 -> 2.053 Scan# : 86 -> 86 - 402 -> 618

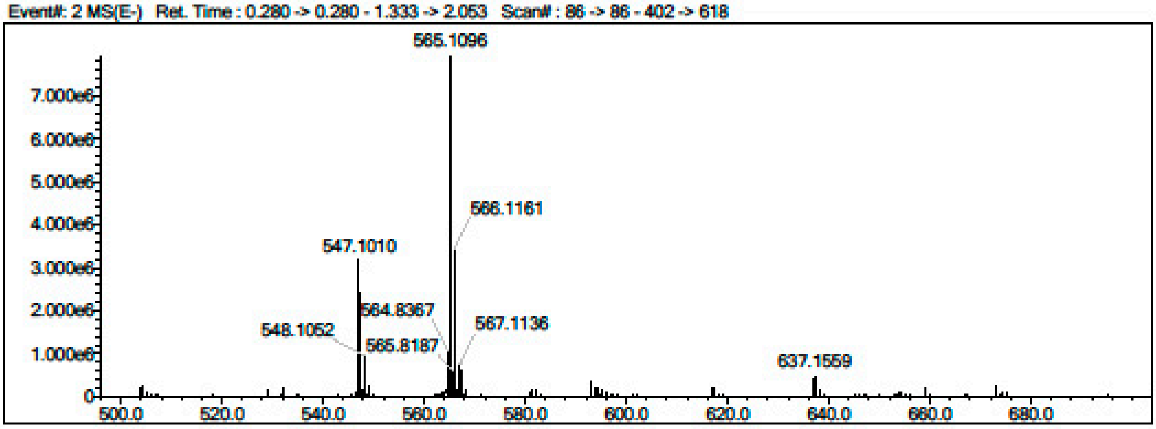

Measured region for 637.1559 m/z

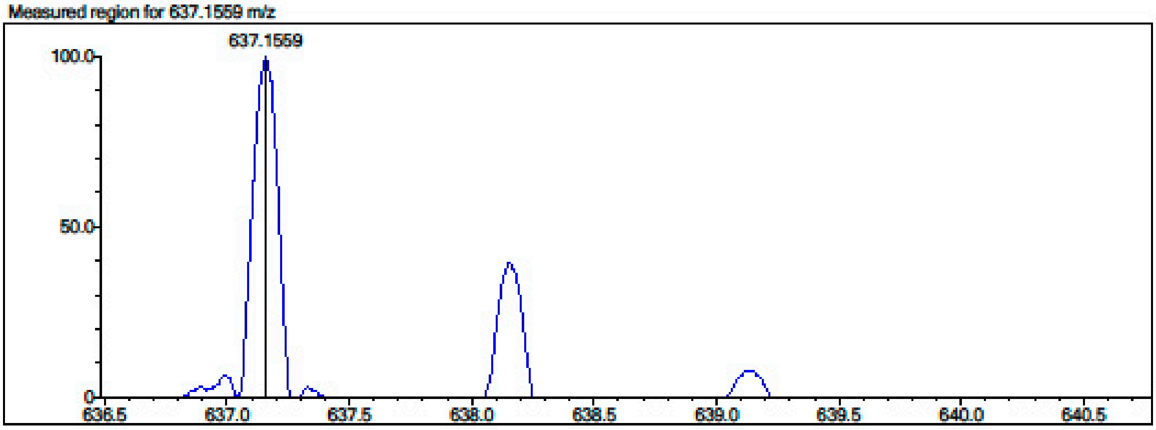

C32 H30 O14 [M-H]- : Predicted region for 637.1563 m/z

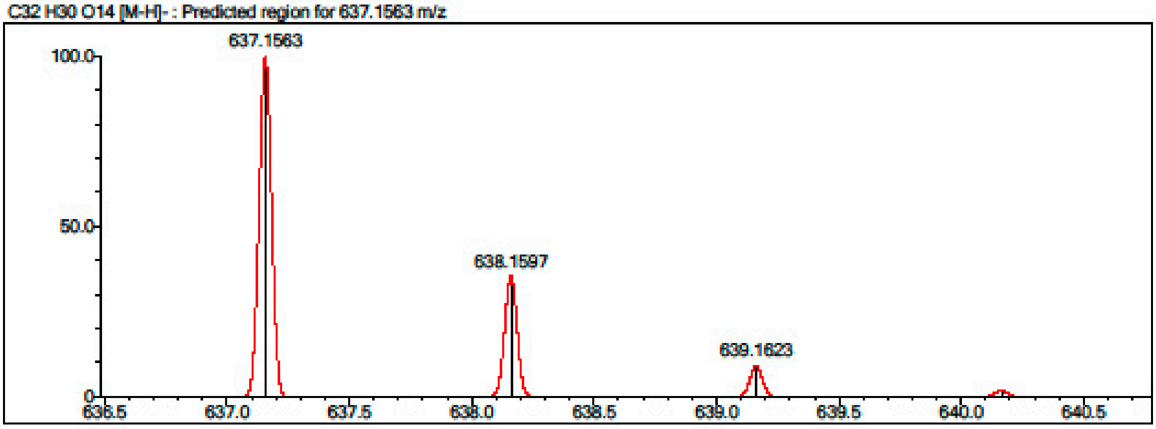

Rank	Score	Formula (M)	Ion	Meas. m/z	Pred. m/z	Df. (mDa)	Df. (ppm)	Iso	DBE
1	64.60	C32 H30 O14	[M-H]-	637.1559	637.1563	-0.4	-0.63	64.60	18.0

Figure S16. CD spectra of compound 2 (alterporriol S)in acetonitrile solution.

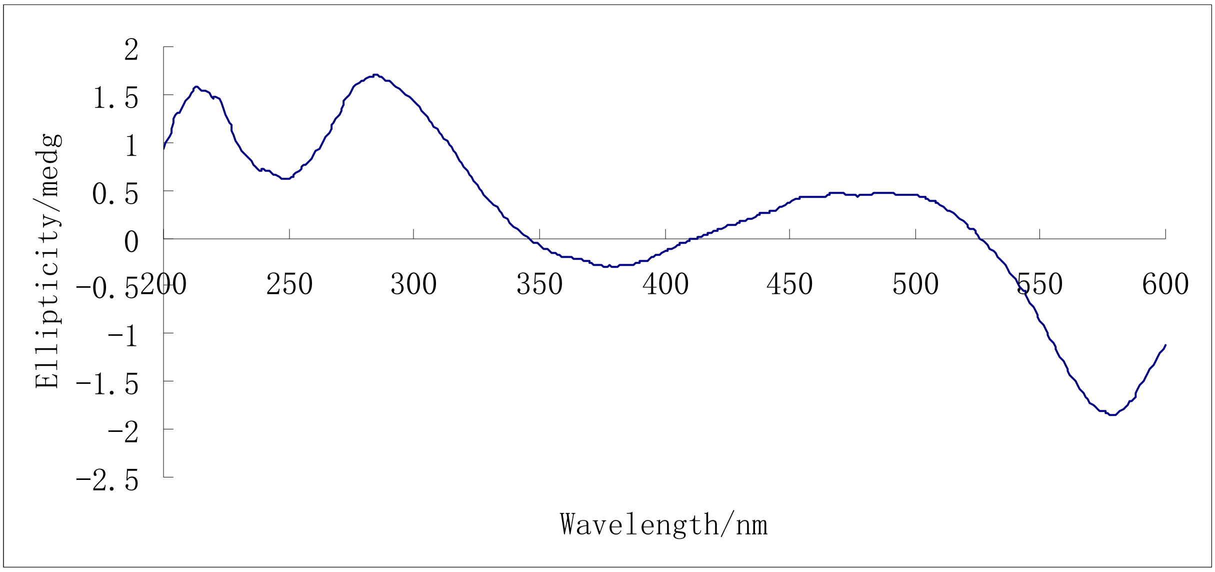

Figure S17. ^{1}H-NMR Spectra of compound 3 (alterporriol T), Measured at 400 MHz (DMSO- *d*6).

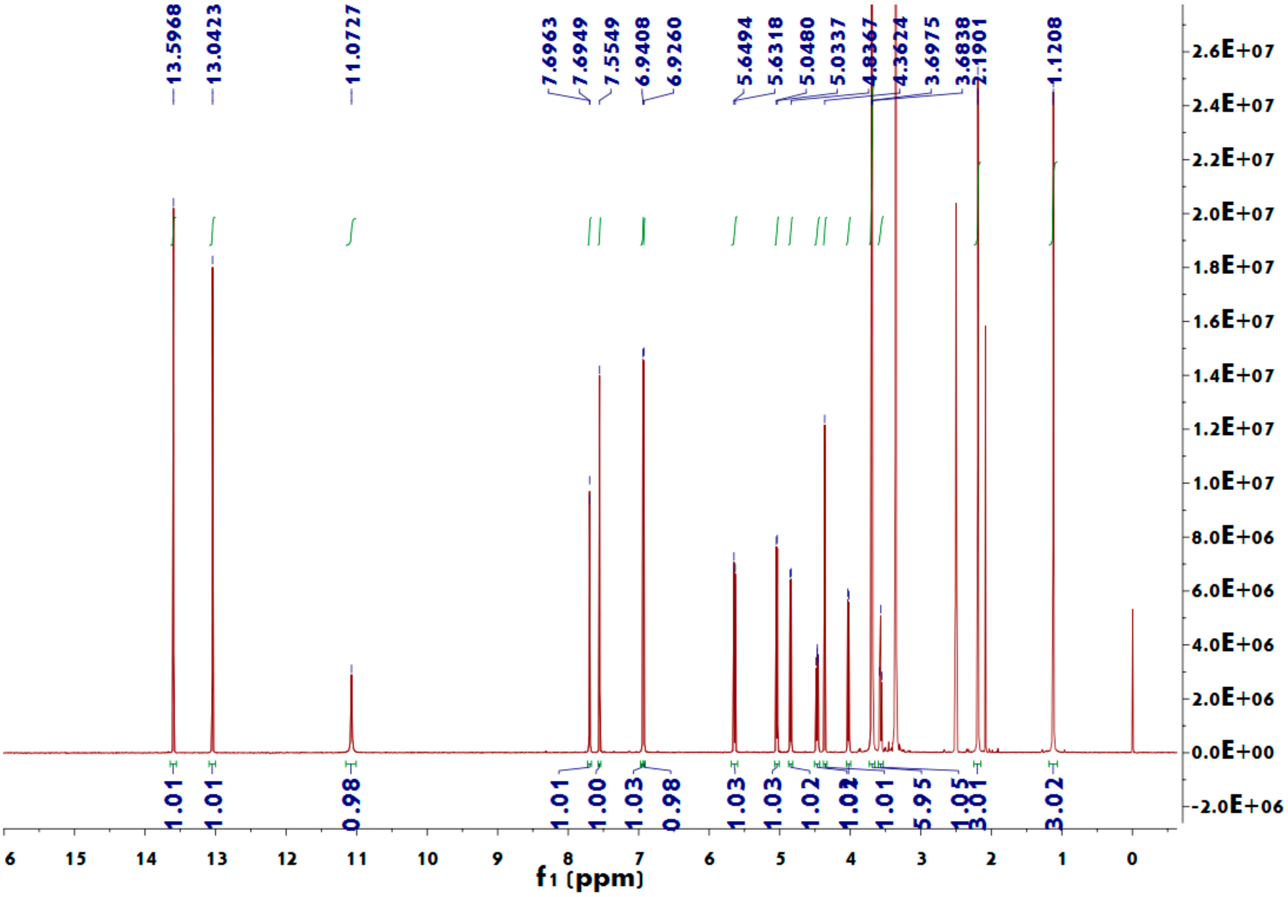

Figure S18. ^{13}C-NMR spectra of compound 3 (alterporriol T), measured at 100 MHz (DMSO- *d*6).

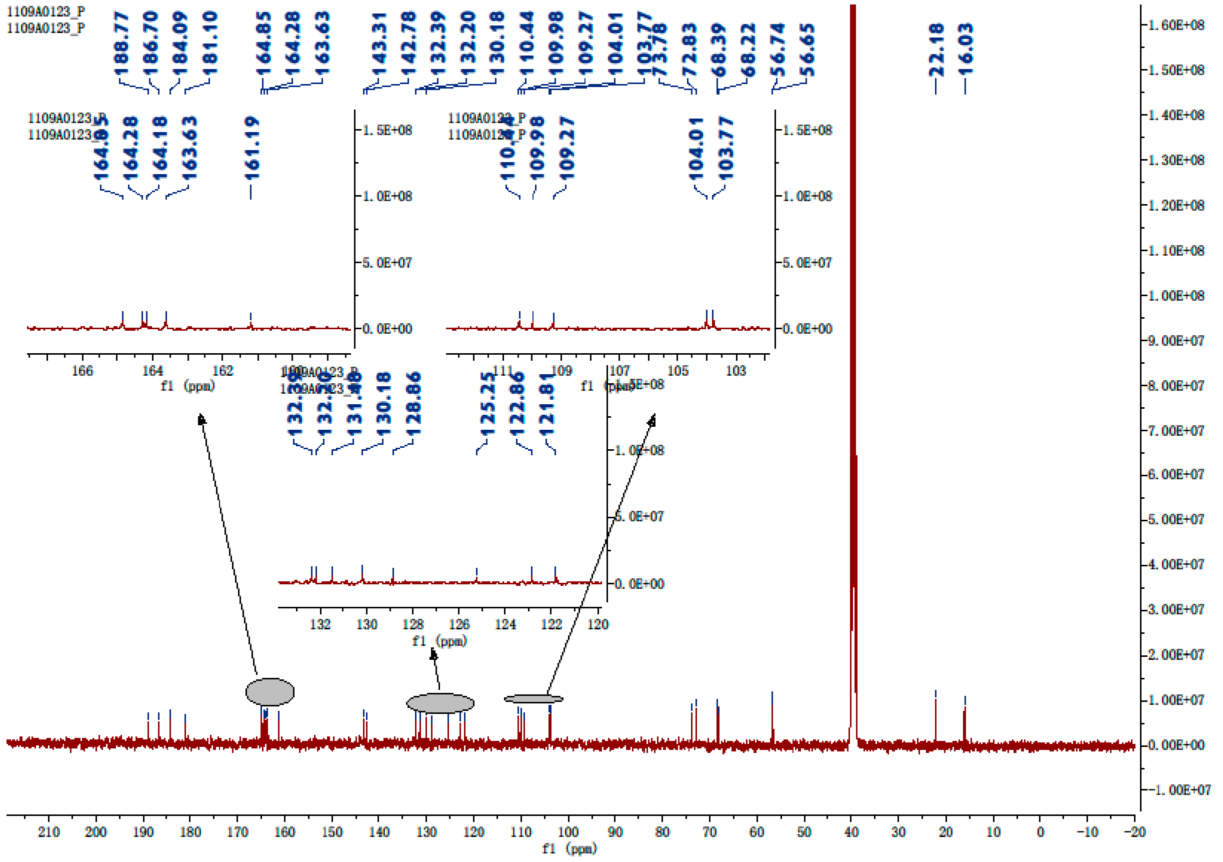

Figure S19. ^{1}H-^{1}H COSY of compound 3 (alterporriol T), measured at 400 MHz (DMSO- *d*6).

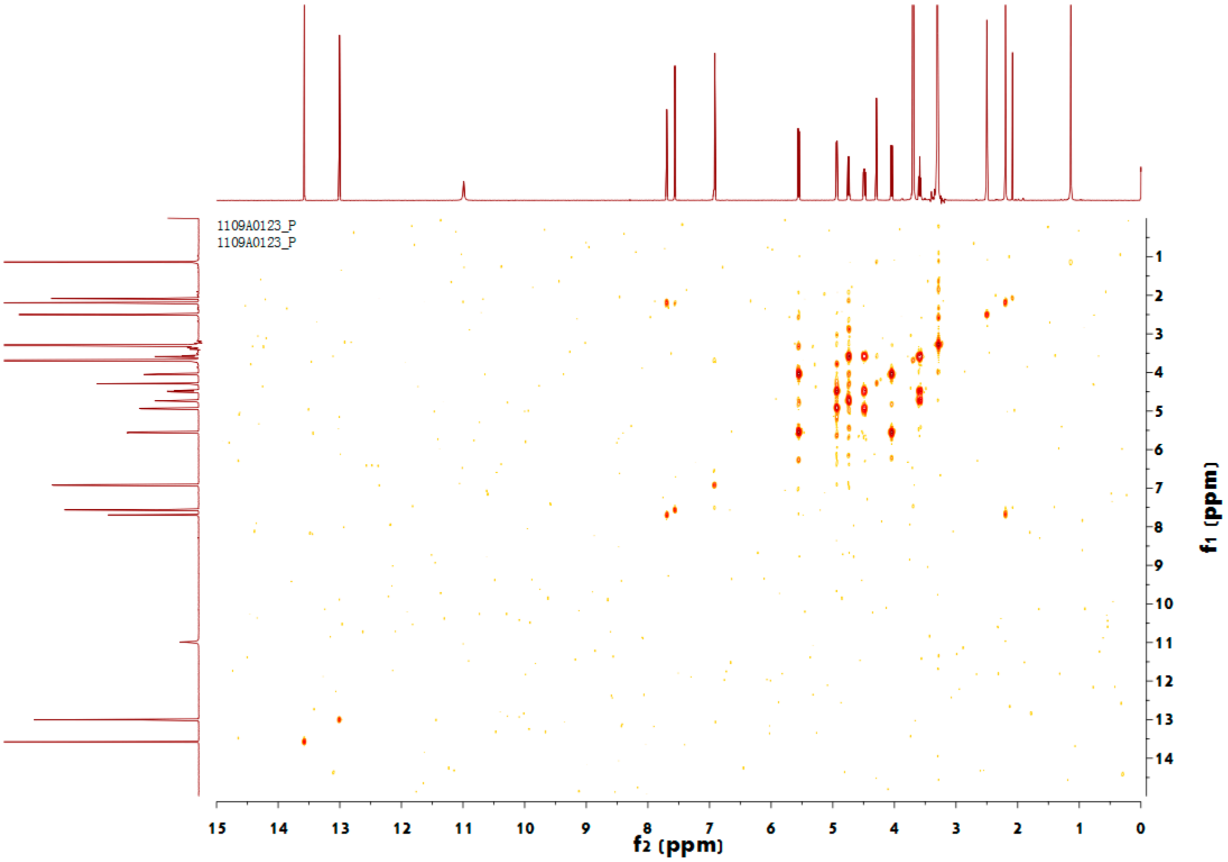

Figure S20. HSQC of compound 3 (alterporriol S), measured at 400 MHz (^{1}H) and 100 MHz (^{13}C) (DMSO- *d*6).

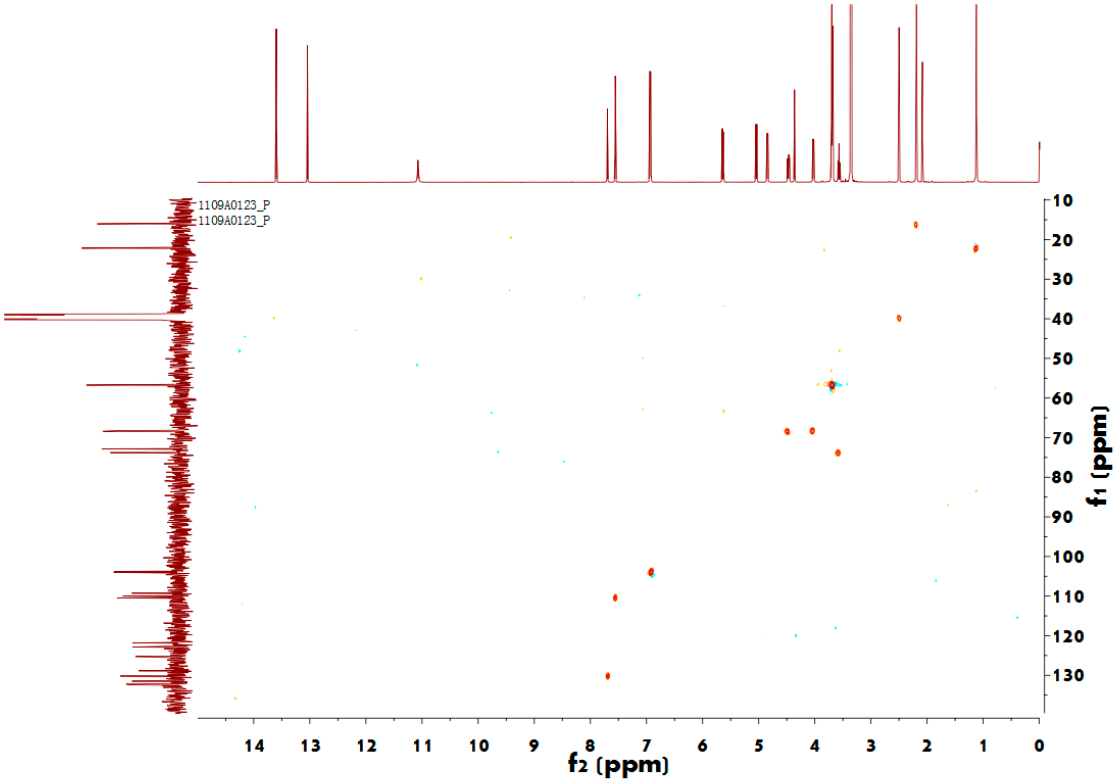

Figure S21. HMBC of compound 3 (alterporriol S), measured at 400 MHz (^{1}H) and 100 MHz (^{13}C) (DMSO- *d*6).

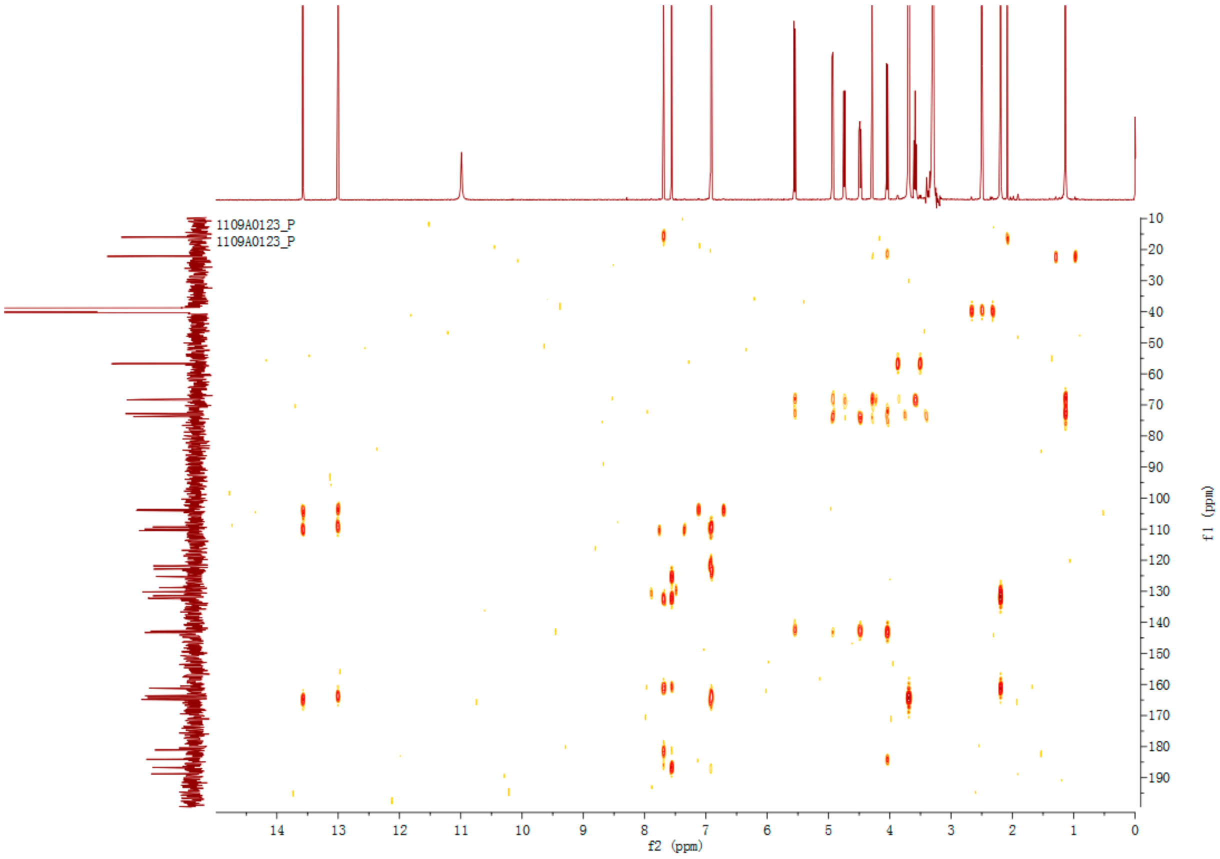

Figure S22. NOESY of compound 3 (alterporriol S), measured at 400 MHz (^{1}H) and 100 MHz (^{13}C) (DMSO- *d*6).

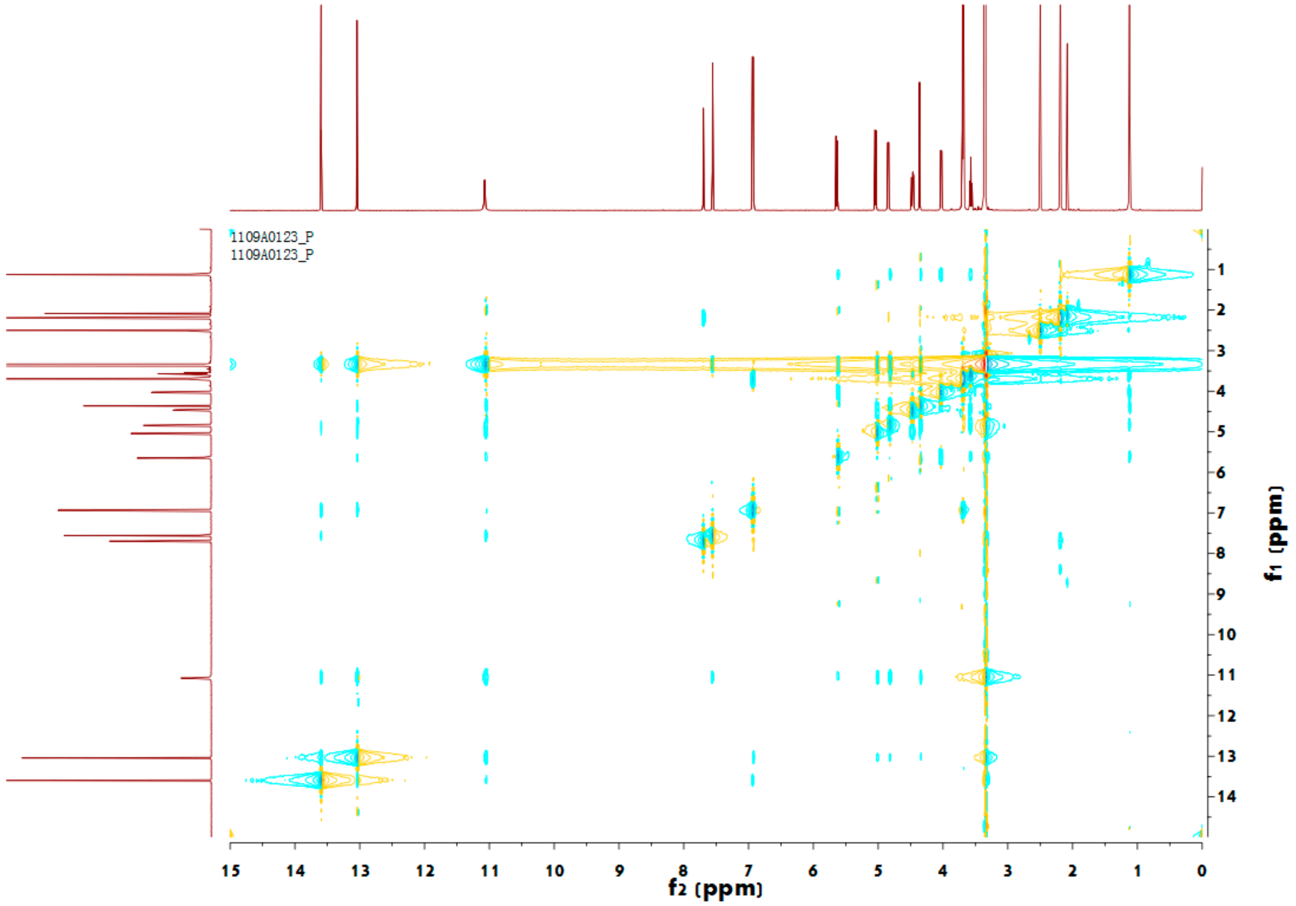

Figure S23. HR-ESI-TOF-MS spectra of compound 3 (alterporriol S).

Formula Predictor Report - 618A.lcd Page 1 of 1

Data File: F:\wang jun\xl\618A.lcd

Elmt	Val.	Min	Max	Elmt	Val.	Min	Max	Elmt	Val.	Min	Max	Elmt	Val.	Min	Max	Use Adduct
H	1	0	35	N	3	0	0	P	3	0	0	Br	1	0	0	H
B	3	0	0	O	2	0	15	S	2	0	0	I	3	0	0	
C	4	0	35	F	1	0	0	Cl	1	0	0	Pt	2	0	0	

Error Margin (ppm): 200
HC Ratio: unlimited
Max Isotopes: all
MSn Iso RI (%): 90.00

DBE Range: 0.0 - 3000.0
Apply N Rule: yes
Isotope RI (%): 1.00
MSn Logic Mode: AND

Electron Ions: both
Use MSn Info: yes
Isotope Res: 10000
Max Results: 500

Event#: 2 MS(E-) Ret. Time : 0.880 Scan# : 266

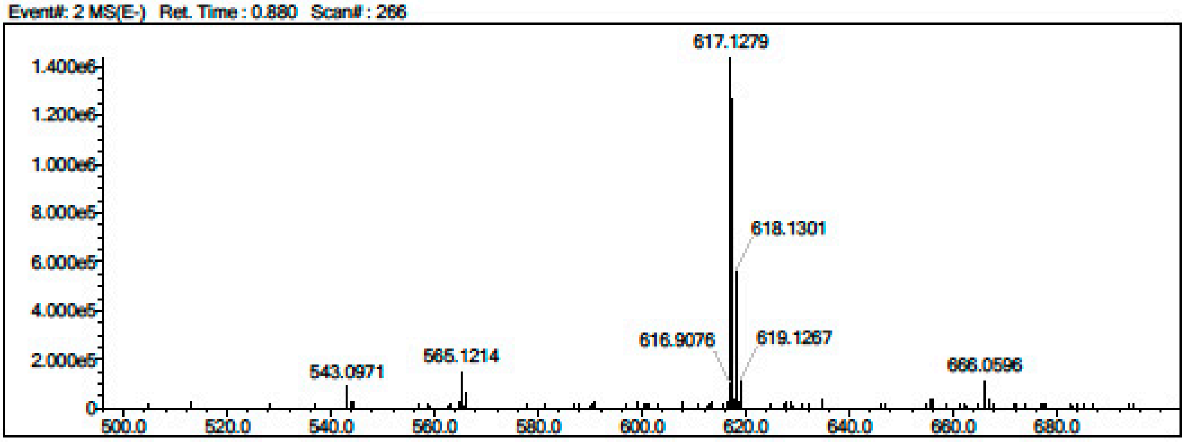

Measured region for 617.1279 m/z

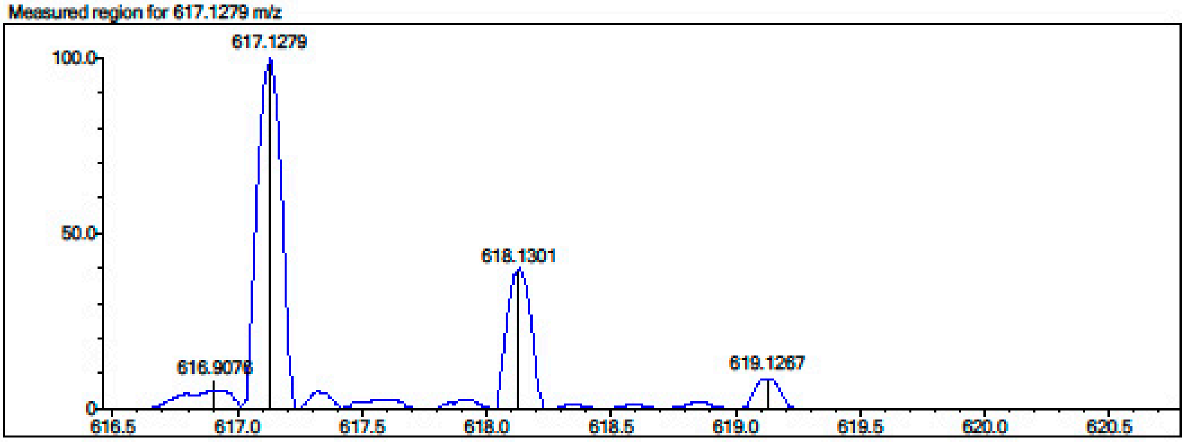

C32 H26 O13 [M-H]- : Predicted region for 617.1301 m/z

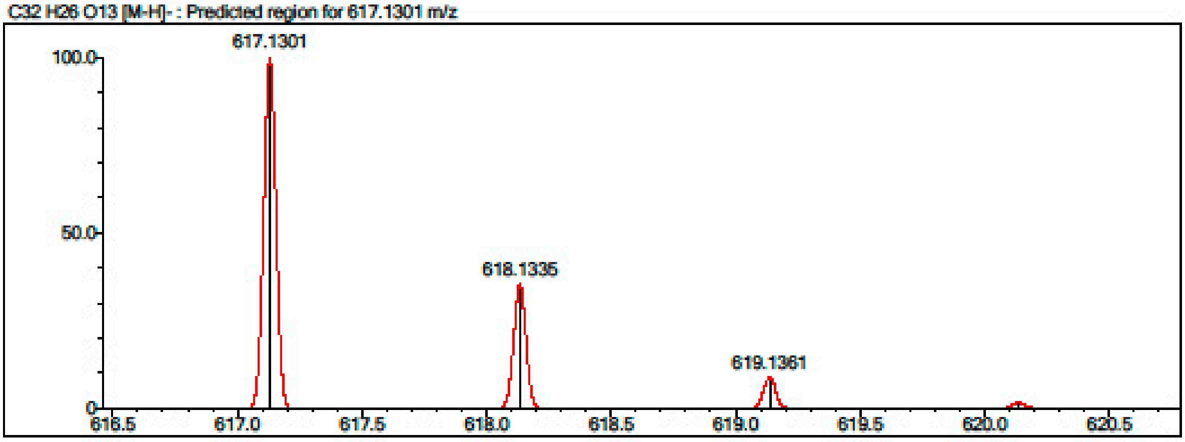

Rank	Score	Formula (M)	Ion	Meas. m/z	Pred. m/z	Df. (mDa)	Df. (ppm)	Iso	DBE
1	80.64	C32 H26 O13	[M-H]-	617.1279	617.1301	-2.2	-3.56	86.15	20.0

Figure S24. CD spectra of compound 3 (alterporriol S) in acetonitrile solution.

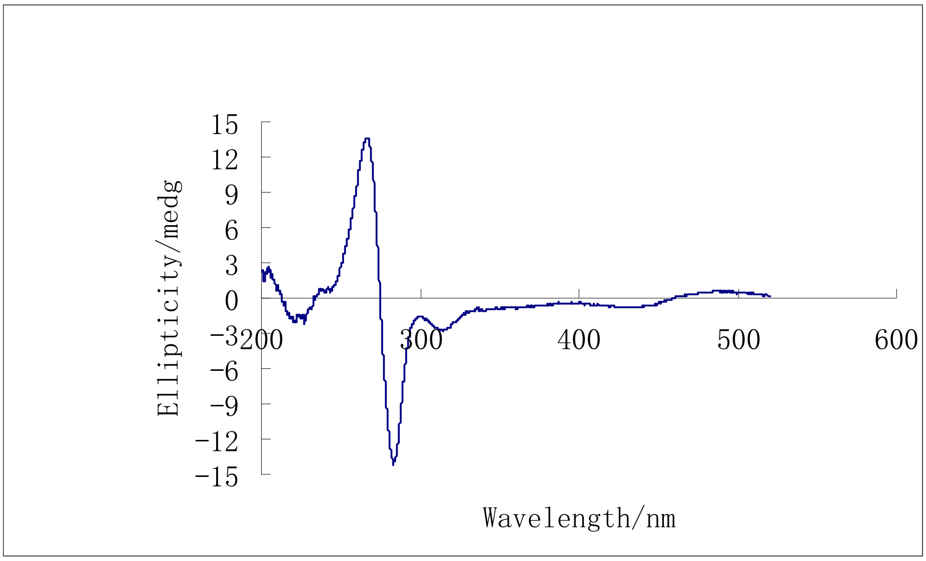

Figure S25. ^{1}H-NMR spectra of compound 4 (alterporriol U), measured at 400 MHz (DMSO- *d*6).

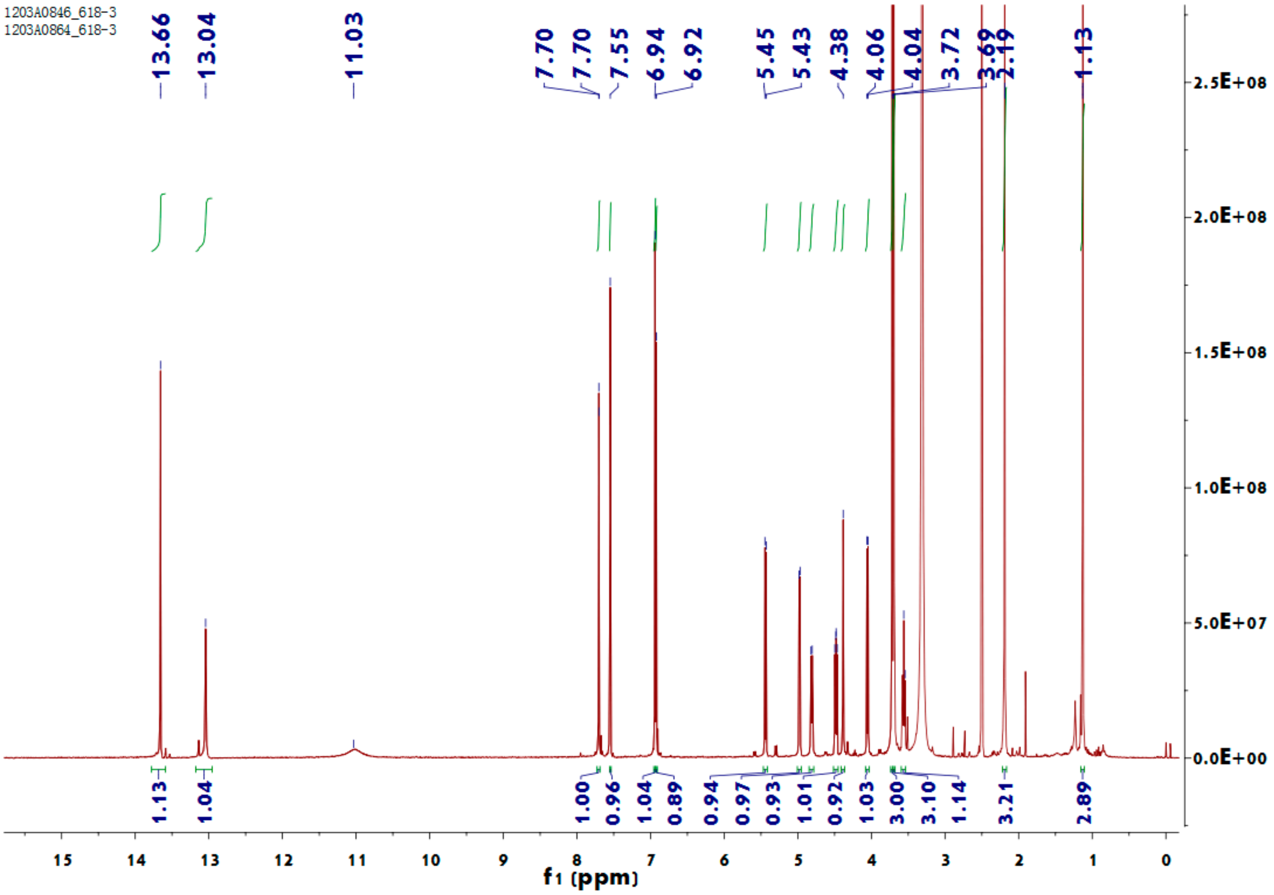

Figure S26. ^{13}C-NMR spectra of compound 4 (alterporriol U), measured at 100 MHz (DMSO- *d*6).

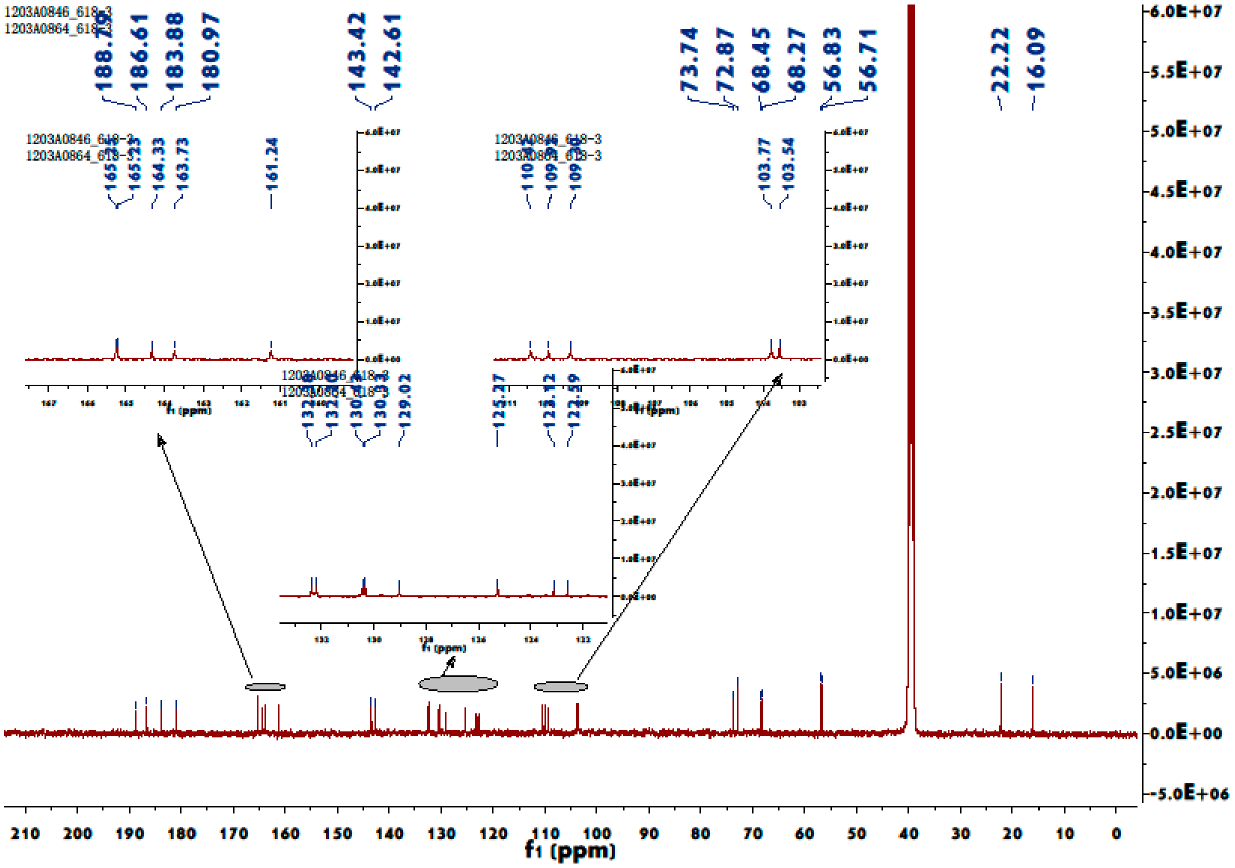

Figure S27. ^{1}H-^{1}H COSY of compound 4 (alterporriol U), measured at 400 MHz (DMSO- *d*6).

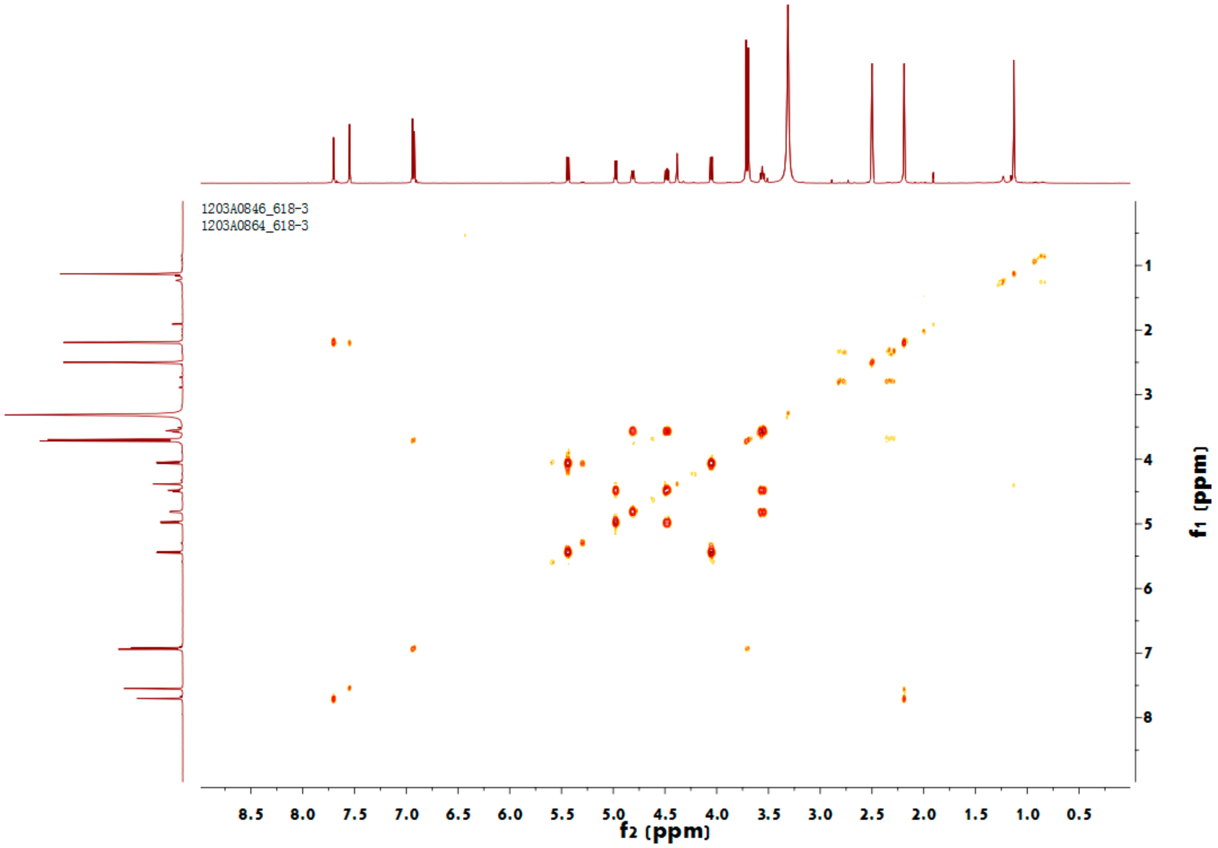

Figure S28. HSQC of compound 4 (alterporriol U), measured at 400 MHz (^{1}H) and 100 MHz (^{13}C) (DMSO- *d*6).

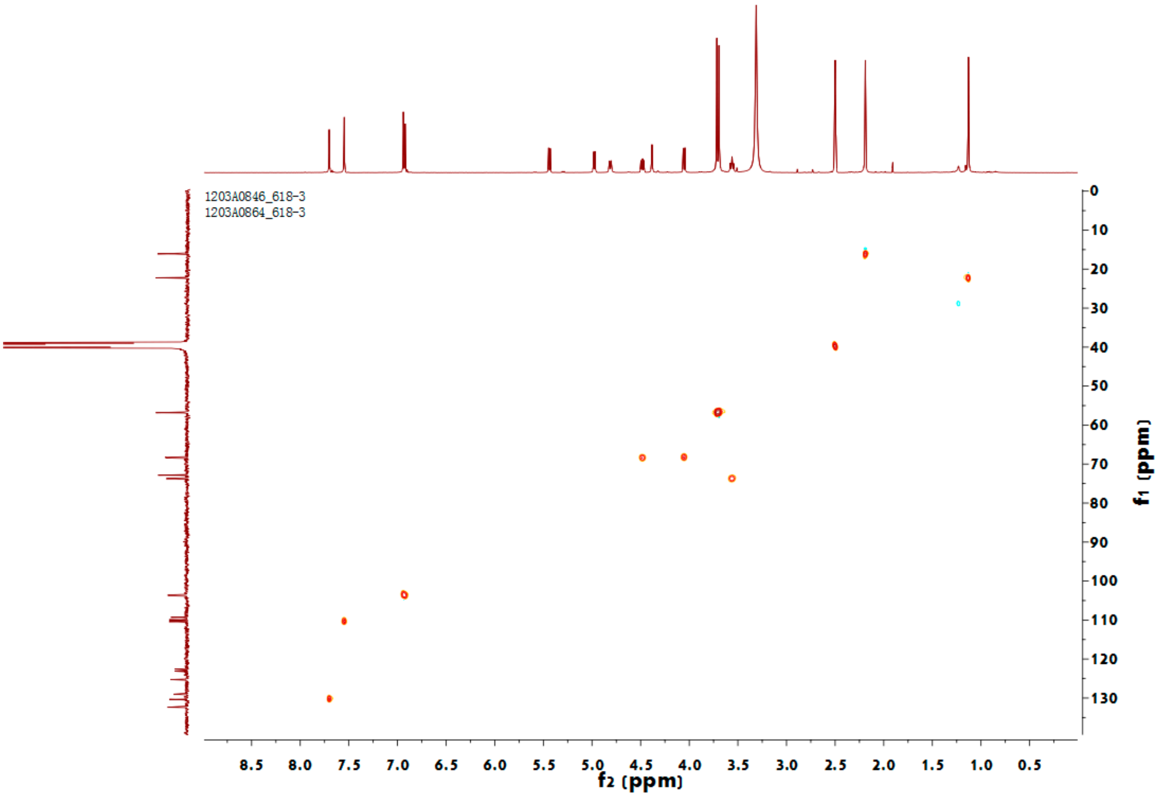

Figure S29. HMBC of compound 4 (alterporriol U), measured at 400 MHz (^{1}H) and 100 MHz (^{13}C) (DMSO- *d*6).

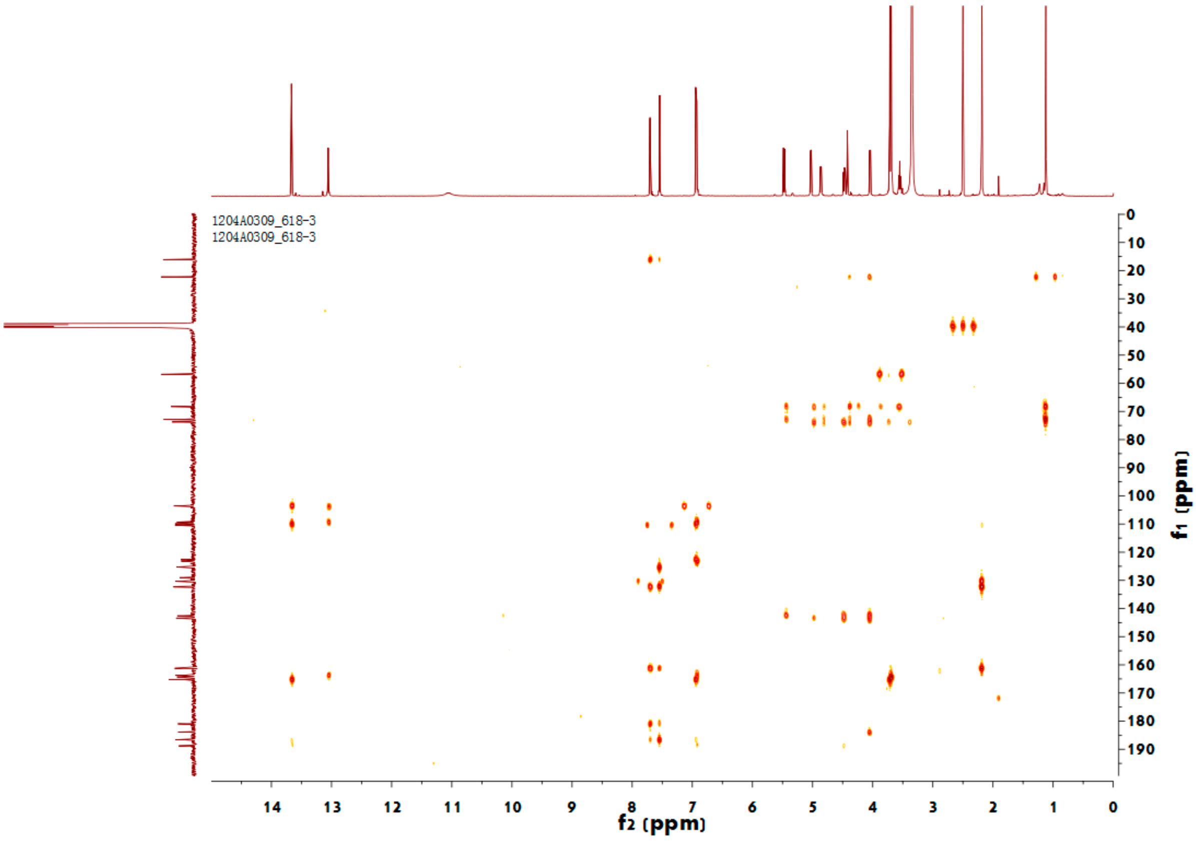

Figure S30. NOESY of compound 4 (alterporriol U), measured at 400 MHz (^{1}H) and 100 MHz (^{13}C) (DMSO- *d*6).

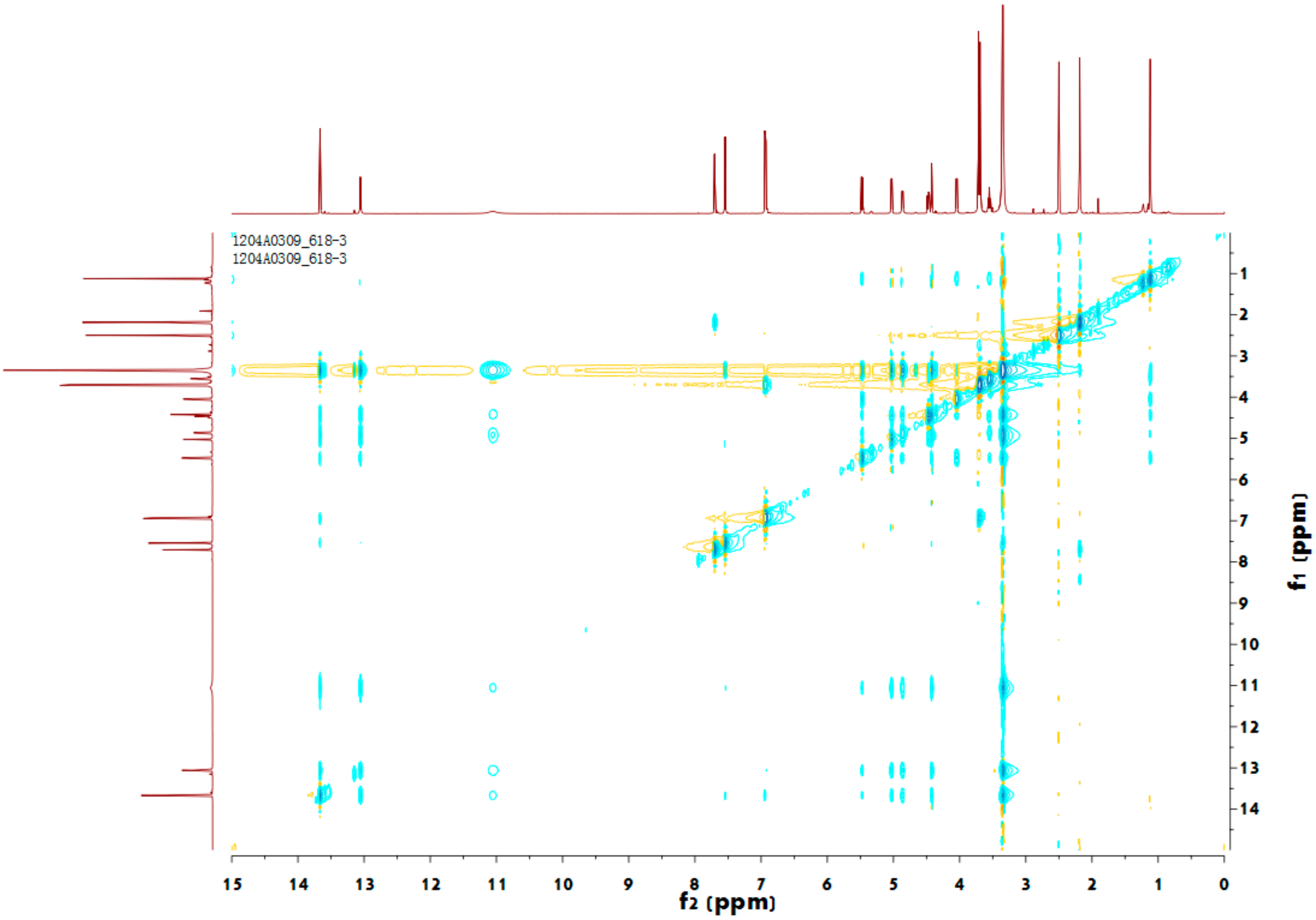

Figure S31. HR-ESI-TOF-MS spectra of compound 4 (alterporriol U).

Formula Predictor Report - D3.lcd　　　　Page 1 of 1

Data File: F:\wang jun\xl\D3.lcd

Elmt	Val.	Min	Max	Elmt	Val.	Min	Max	Elmt	Val.	Min	Max	Elmt	Val.	Min	Max	Use Adduct
H	1	0	35	N	3	0	0	P	3	0	0	Br	1	0	0	H
B	3	0	0	O	2	0	16	S	2	0	0	I	3	0	0	
C	4	0	35	F	1	0	0	Cl	1	0	0	Pt	2	0	0	

Error Margin (ppm):	200	DBE Range:	0.0 - 3000.0	Electron Ions:	both
HC Ratio:	unlimited	Apply N Rule:	yes	Use MSn Info:	yes
Max Isotopes:	all	Isotope RI (%):	1.00	Isotope Res:	10000
MSn Iso RI (%):	90.00	MSn Logic Mode:	AND	Max Results:	500

Event#: 2 MS(E-)　Ret. Time : 0.327 -> 0.327 - 1.693 -> 2.158　Scan# : 100 -> 100 - 510 -> 650

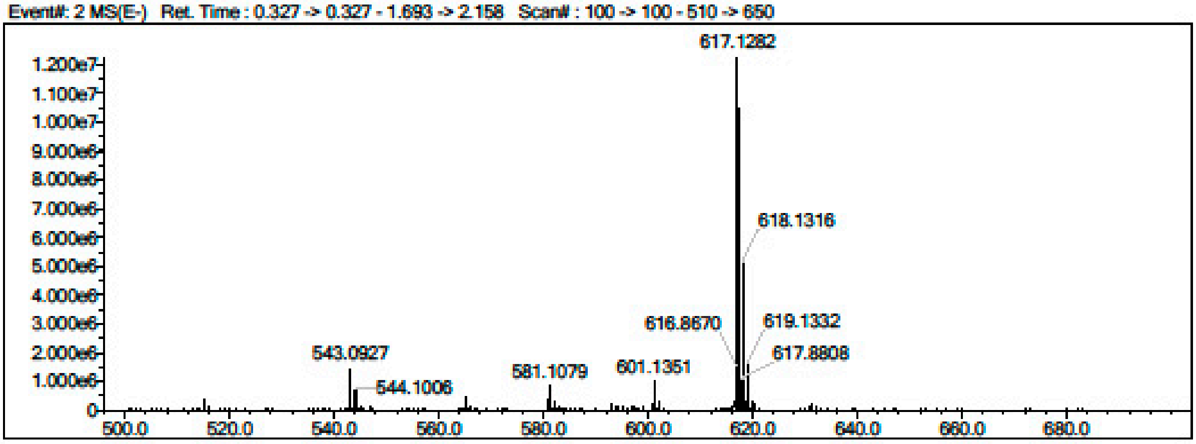

Measured region for 617.1282 m/z

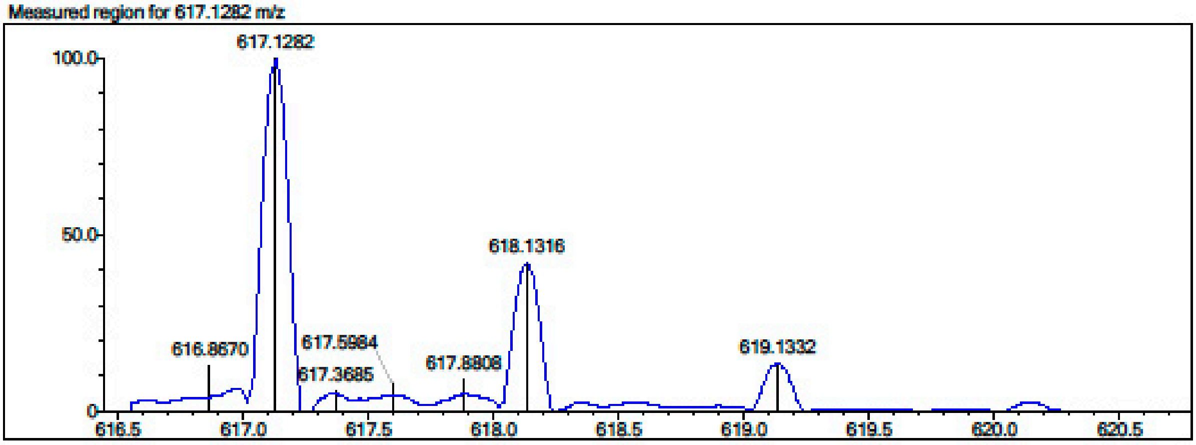

C32 H26 O13 [M-H]- : Predicted region for 617.1301 m/z

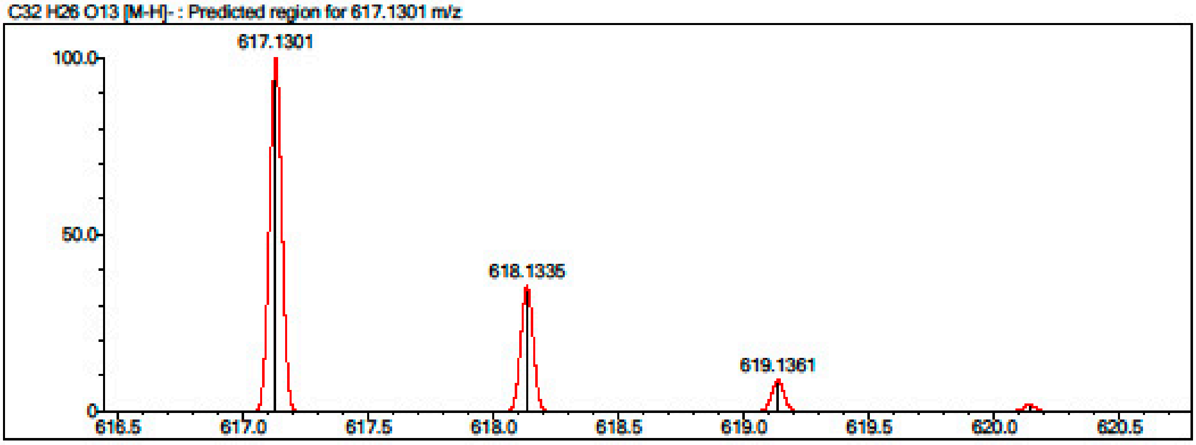

Rank	Score	Formula (M)	Ion	Meas. m/z	Pred. m/z	Df. (mDa)	Df. (ppm)	Iso	DBE
1	80.07	C32 H26 O13	[M-H]-	617.1282	617.1301	-1.9	-3.08	84.47	20.0

Figure S32. CD spectra of compound 4 (alterporriol U) in acetonitrile solution.

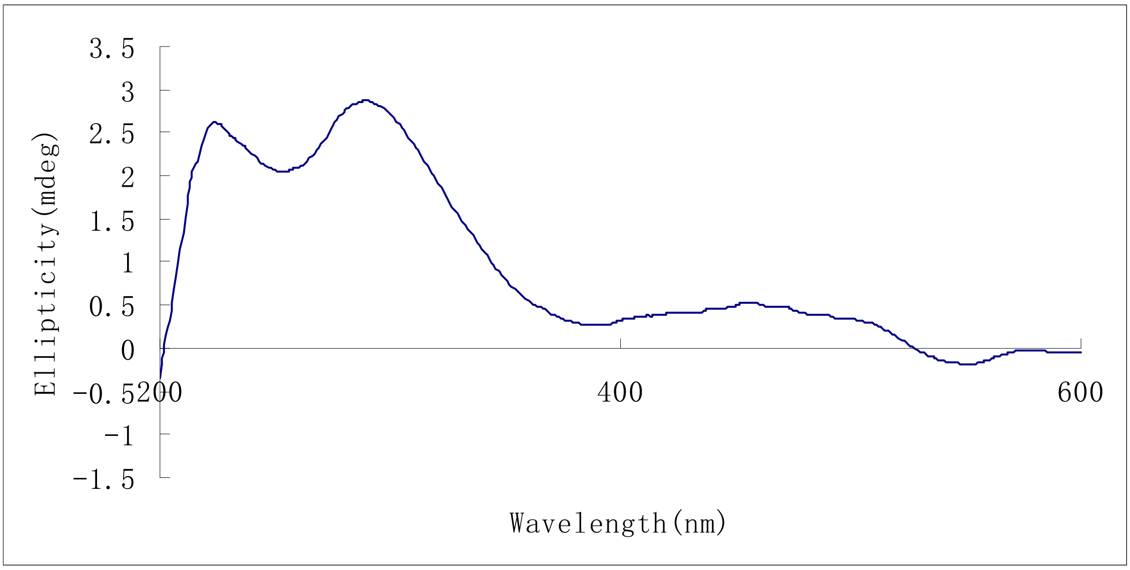

Figure S33. ^{1}H-NMR data of compound 5 (alterporriol E), measured at 400 MHz (DMSO- *d*6).

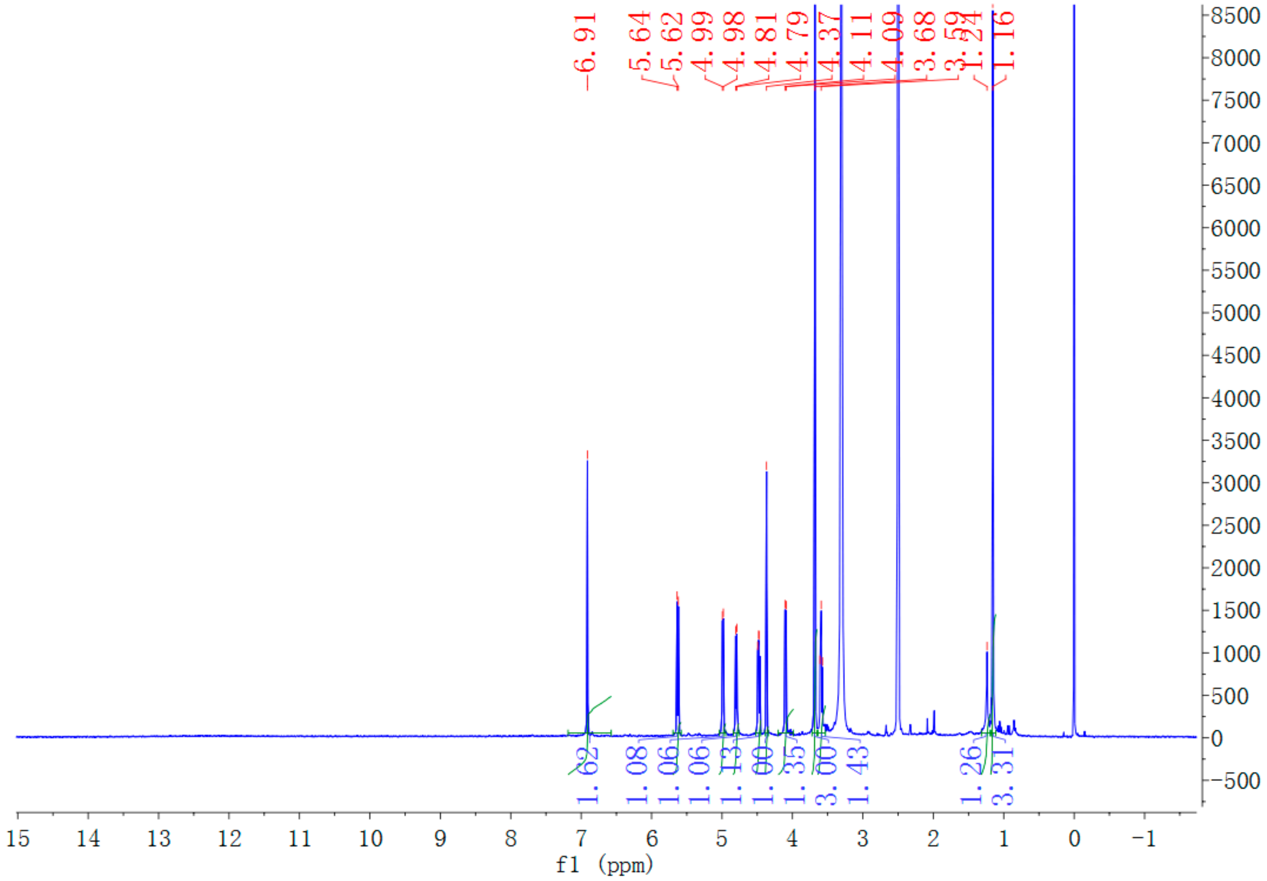

Figure S34. ^{13}C-NMR data of compound 5 (alterporriol E), measured at 100 MHz (DMSO- *d*6).

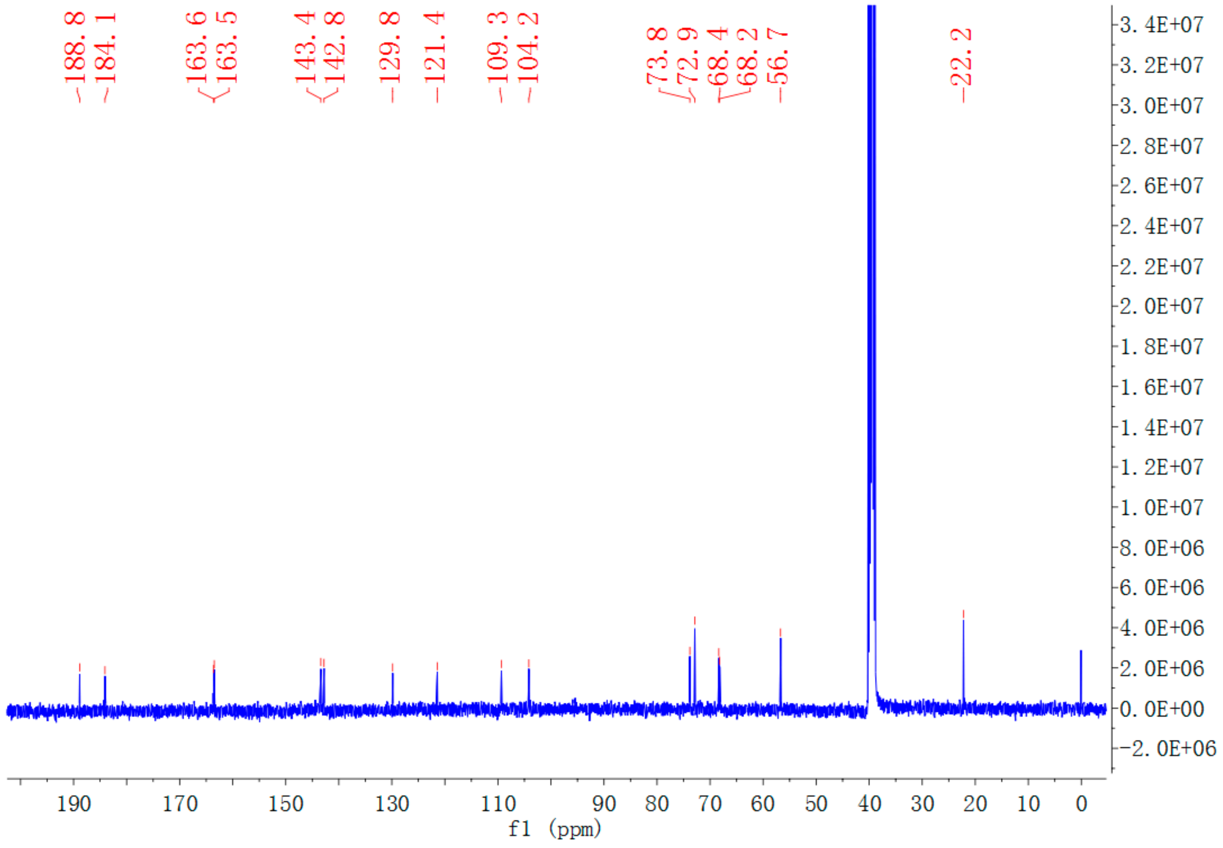

Figure S35. DEPT-90 ^{13}C-NMR data of compound 5 (alterporriol E), measured at 100 MHz (DMSO- *d*6).

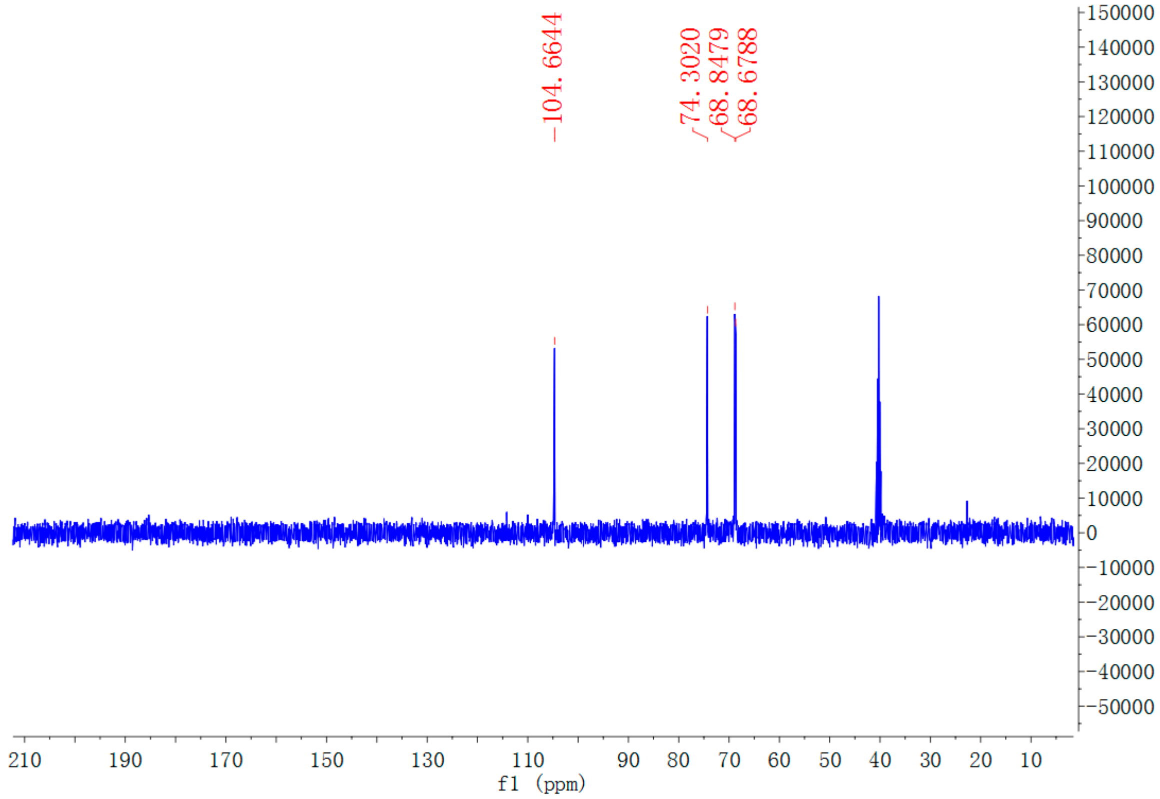

Figure S36. DEPT-135 ^{13}C-NMR data of compound 5 (alterporriol E), measured at 100 MHz (DMSO- *d*6).

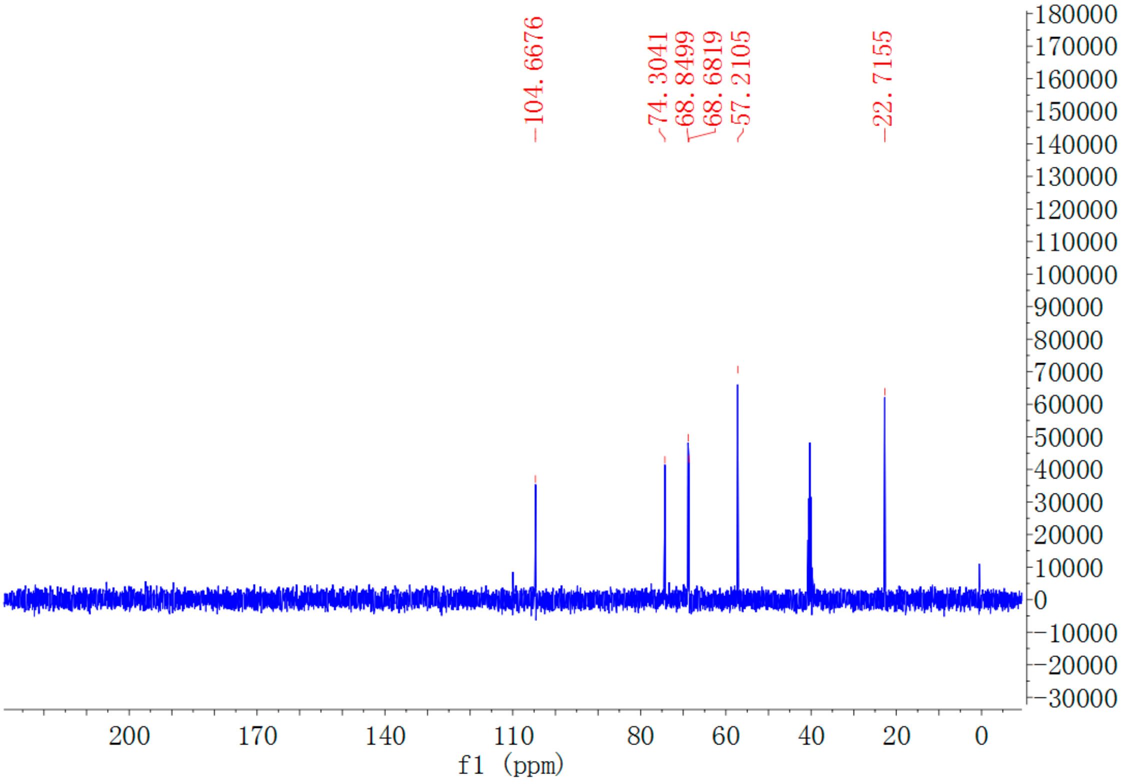

Figure S37. ^{1}H-^{1}H COSY of compound 5 (alterporriol E), measured at 400 MHz (DMSO- *d*6).

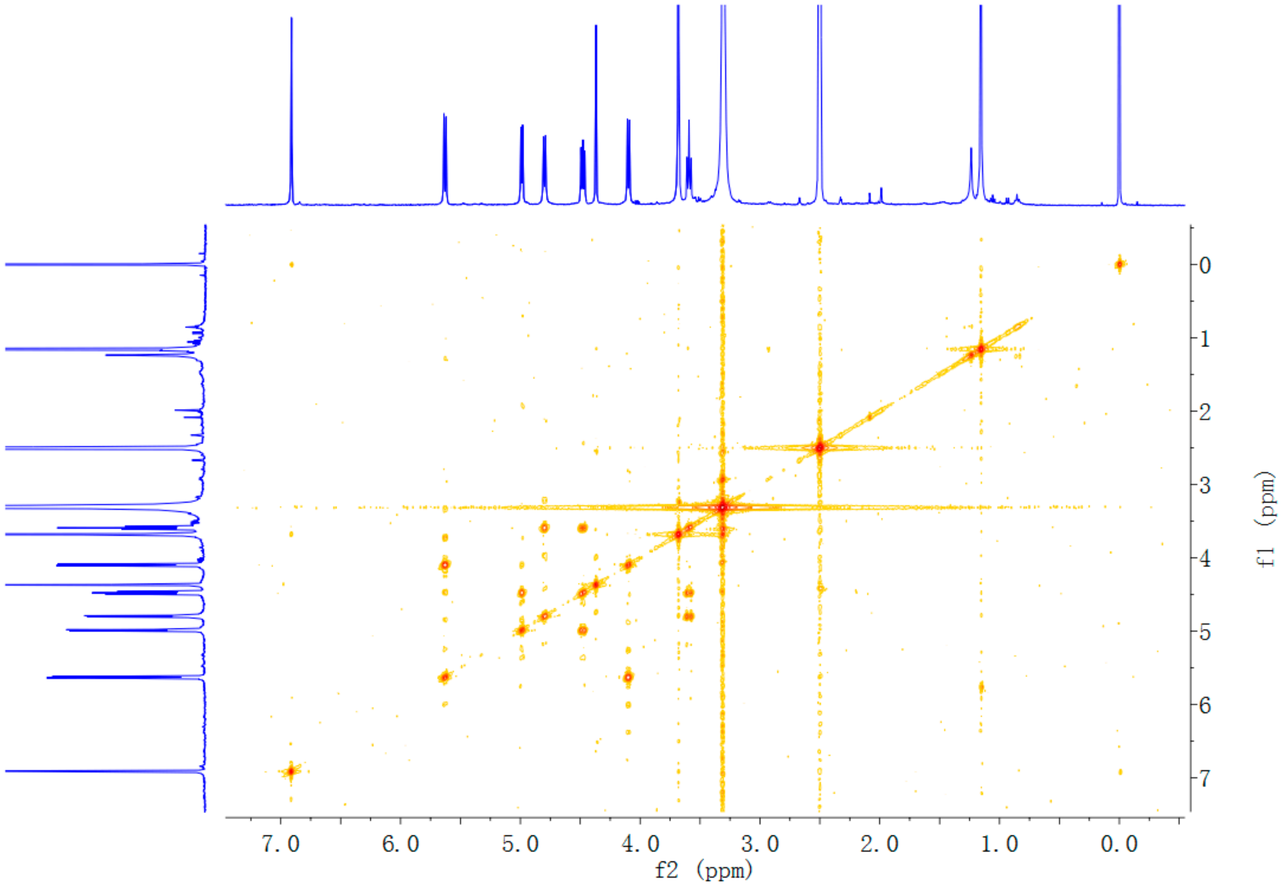

Figure S38. HSQC of compound 5 (alterporriol E), measured at 400 MHz (^{1}H) and 100 MHz (^{13}C) (DMSO- *d*6).

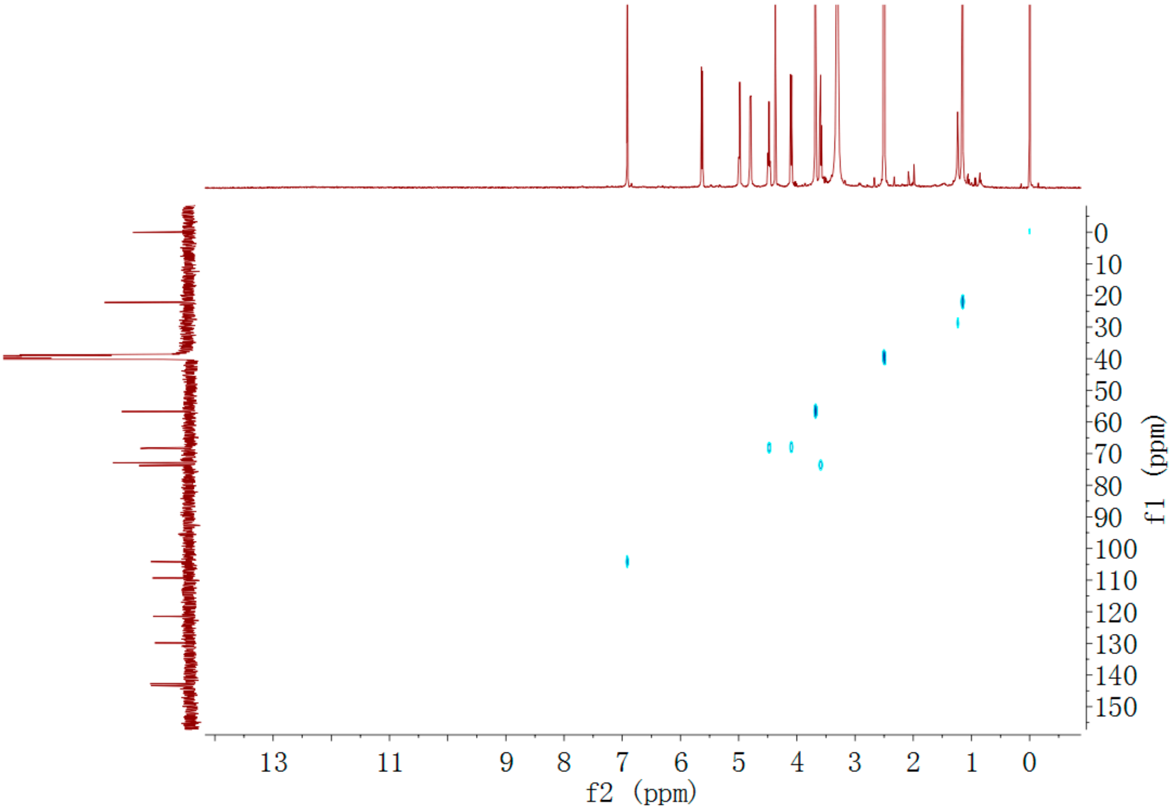

Figure S39. HMBC of compound 5 (alterporriol E), measured at 400 MHz (^{1}H) and 100 MHz (^{13}C) (DMSO- *d*6).

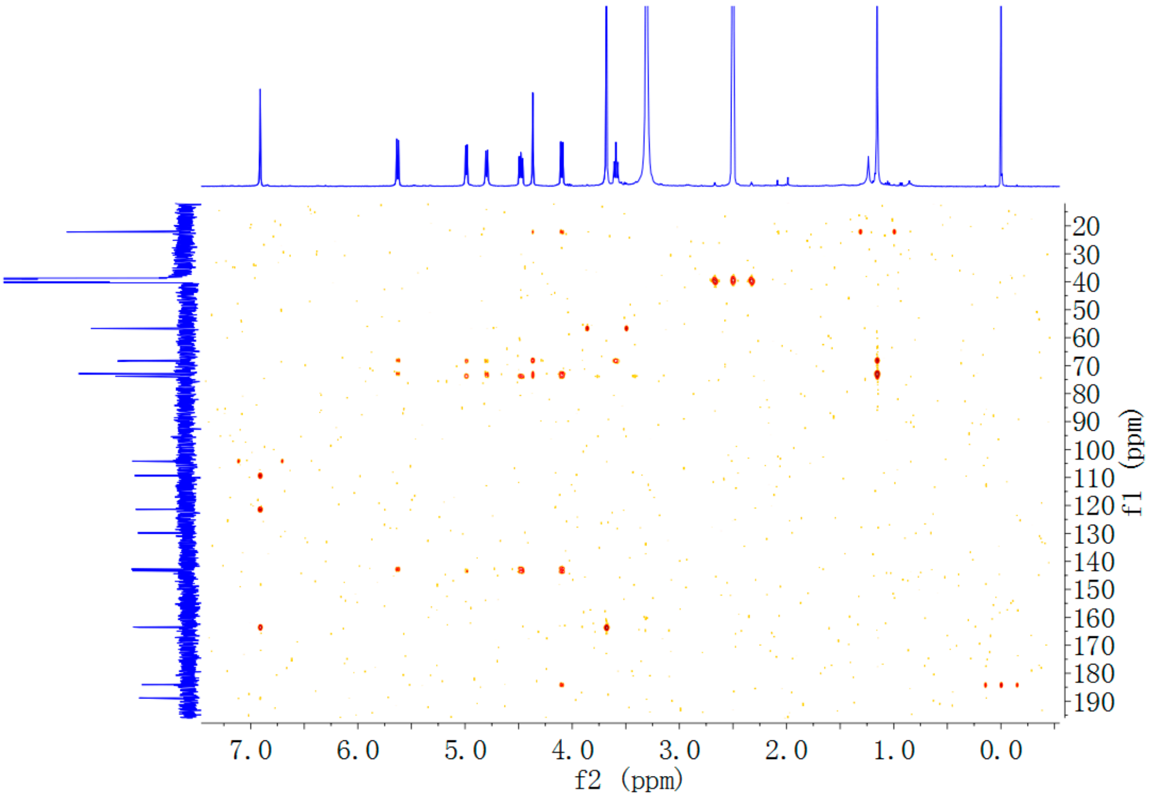

Figure S40. NOESY of compound 5 (alterporriol E), measured at 400 MHz (^{1}H) and 100 MHz (^{13}C) (DMSO- *d*6).

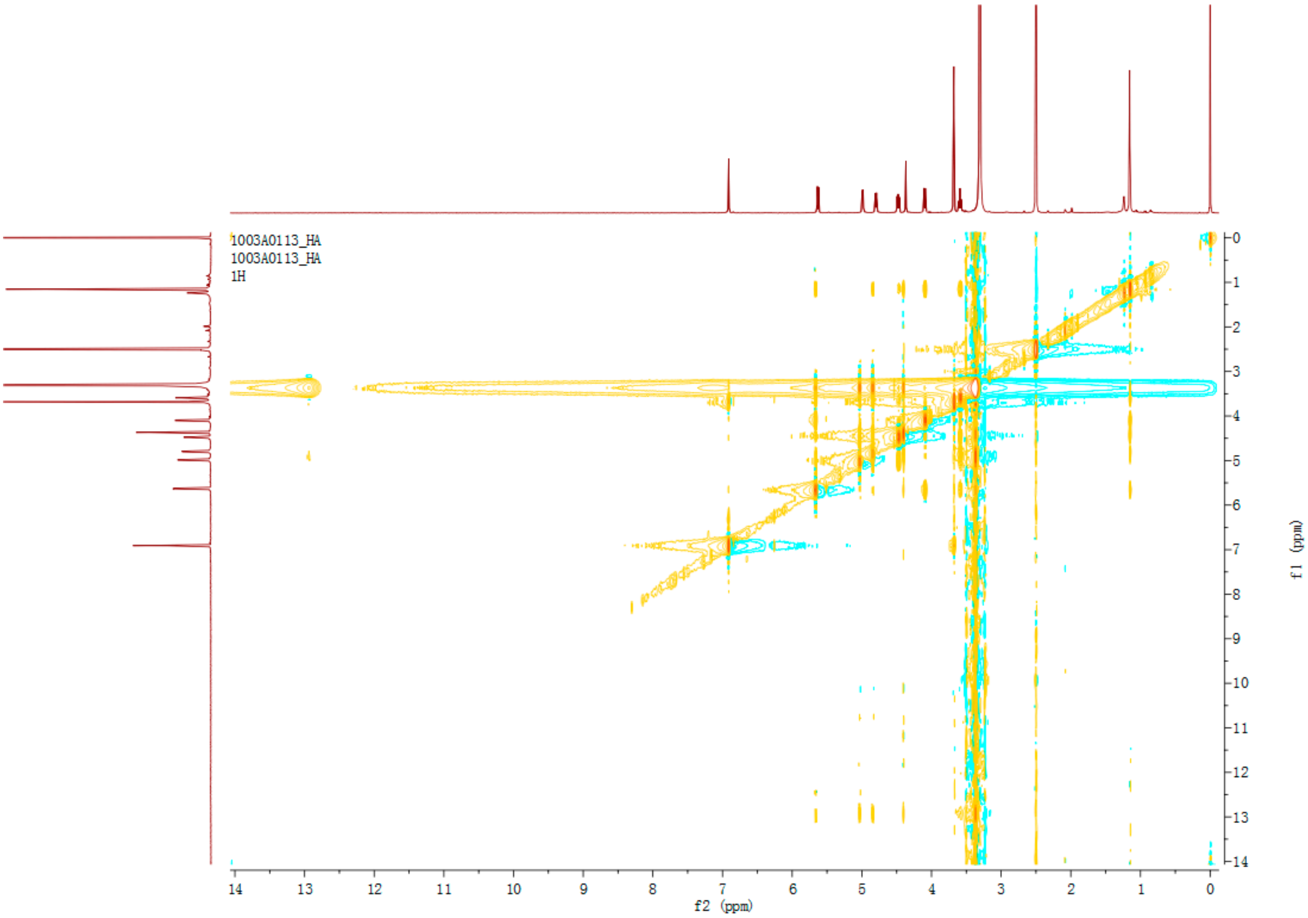

Figure S41. HR-ESI-TOF-MS spectra of compound 1 (alterporriol S).

Formula Predictor Report - 670A.lcd

Page 1 of 1

Data File: F:\wang jun\xl\670A.lcd

Elmt	Val.	Min	Max	Elmt	Val.	Min	Max	Elmt	Val.	Min	Max	Elmt	Val.	Min	Max	Use Adduct
H	1	0	35	N	3	0	0	P	3	0	0	Br	1	0	0	H
B	3	0	0	O	2	0	16	S	2	0	0	I	3	0	0	
C	4	0	35	F	1	0	0	Cl	1	0	0	Pt	2	0	0	

Error Margin (ppm): 200
HC Ratio: unlimited
Max Isotopes: all
MSn Iso RI (%): 90.00

DBE Range: 0.0 - 3000.0
Apply N Rule: yes
Isotope RI (%): 1.00
MSn Logic Mode: AND

Electron Ions: both
Use MSn Info: yes
Isotope Res: 10000
Max Results: 500

Event#: 2 MS(E-) Ret. Time : 0.307 -> 0.307 Scan# : 94 -> 94

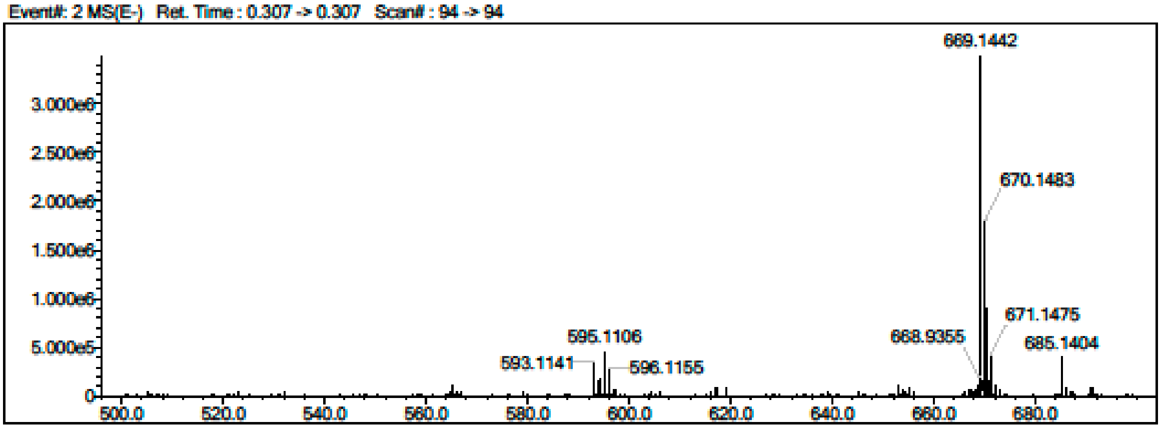

Measured region for 669.1442 m/z

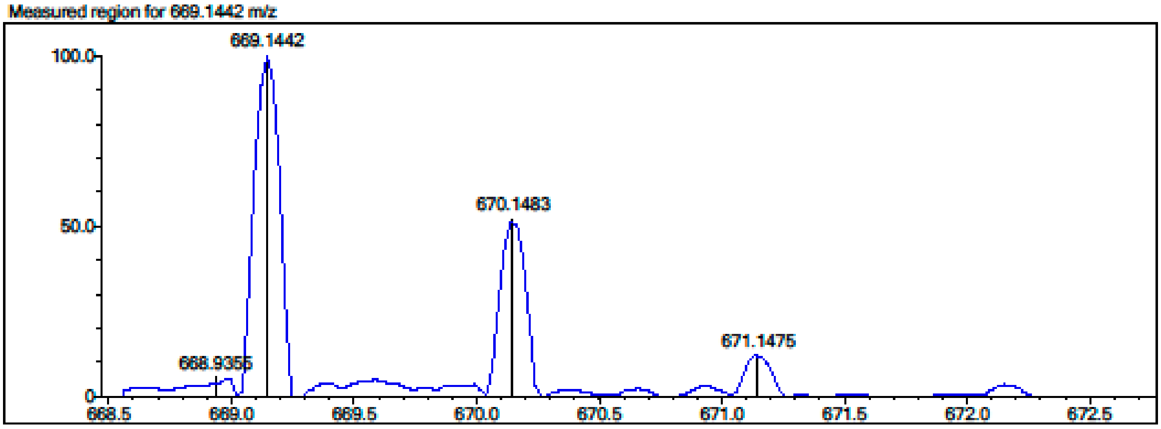

C32 H30 O16 [M-H]- : Predicted region for 669.1461 m/z

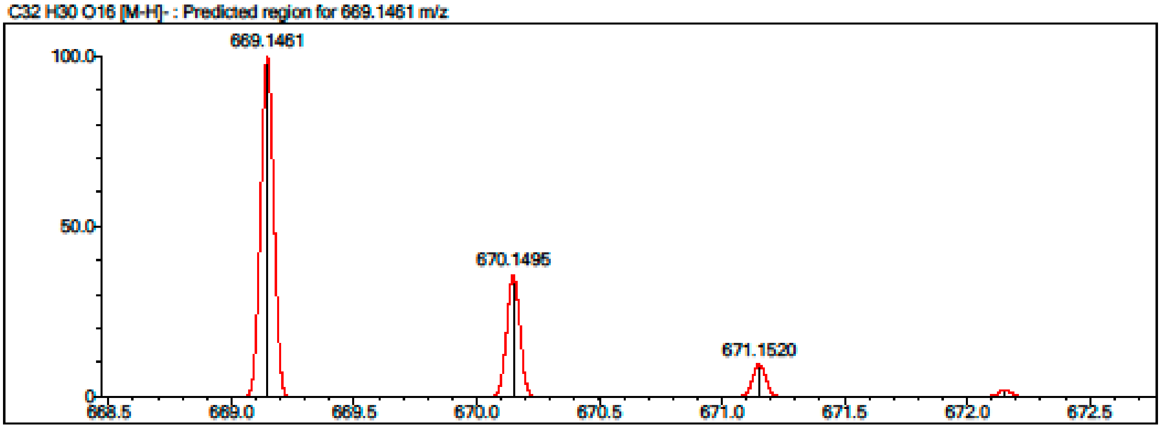

Rank	Score	Formula (M)	Ion	Meas. m/z	Pred. m/z	Df. (mDa)	Df. (ppm)	Iso	DBE
1	57.99	C32 H30 O16	[M-H]-	669.1442	669.1461	-1.9	-2.84	60.79	18.0

Figure S42. CD spectra of compound 5 (alterporriol E) in acetonitrile solution.

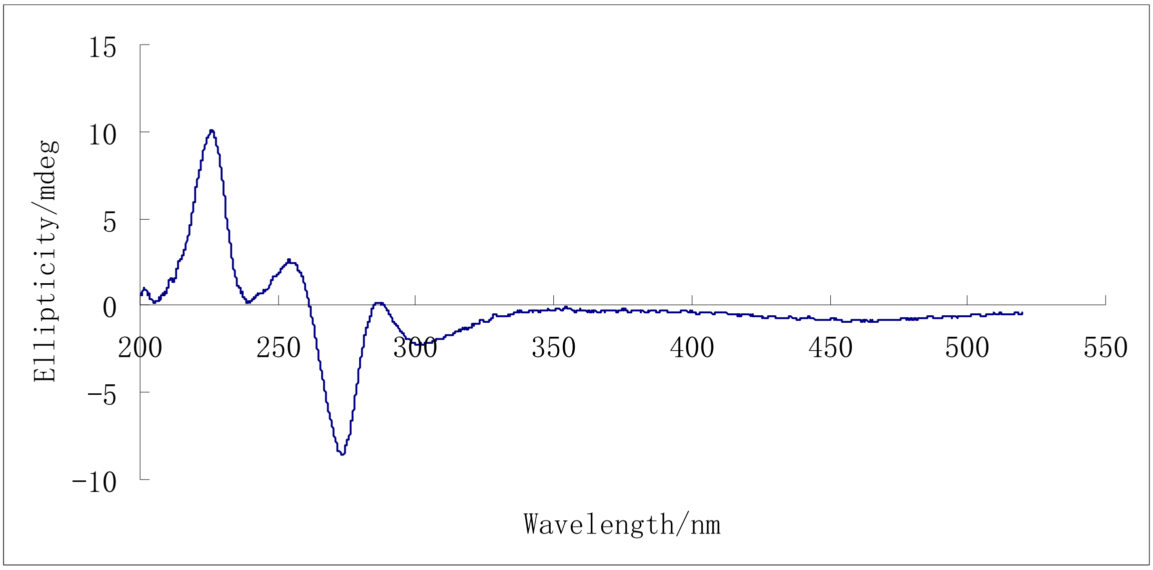

Figure S43. ^{1}H-NMR data of compound 6, measured at 400 MHz (DMSO- *d*6).

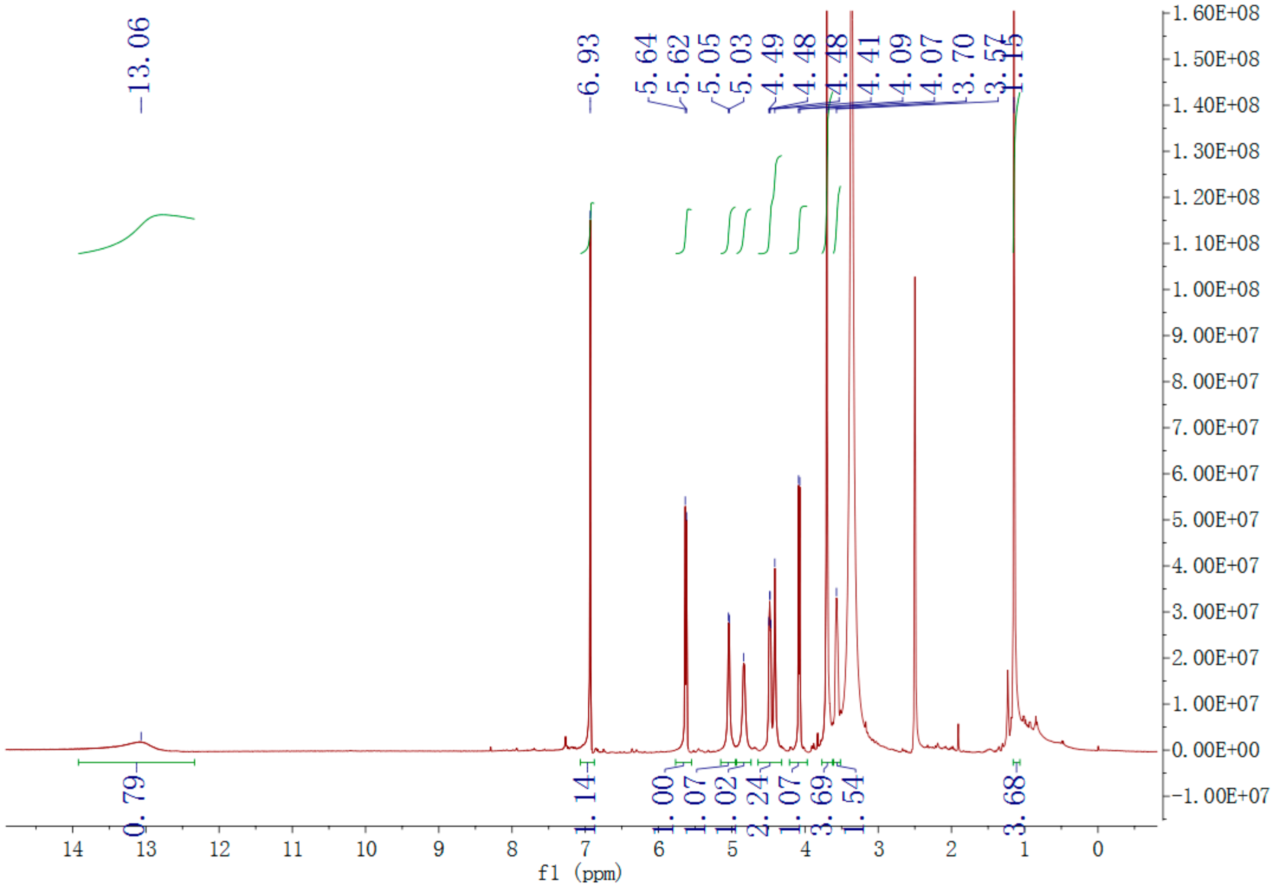

Figure S44. ^{13}C-NMR data of compound 6, measured at 100 MHz (DMSO- *d*6).

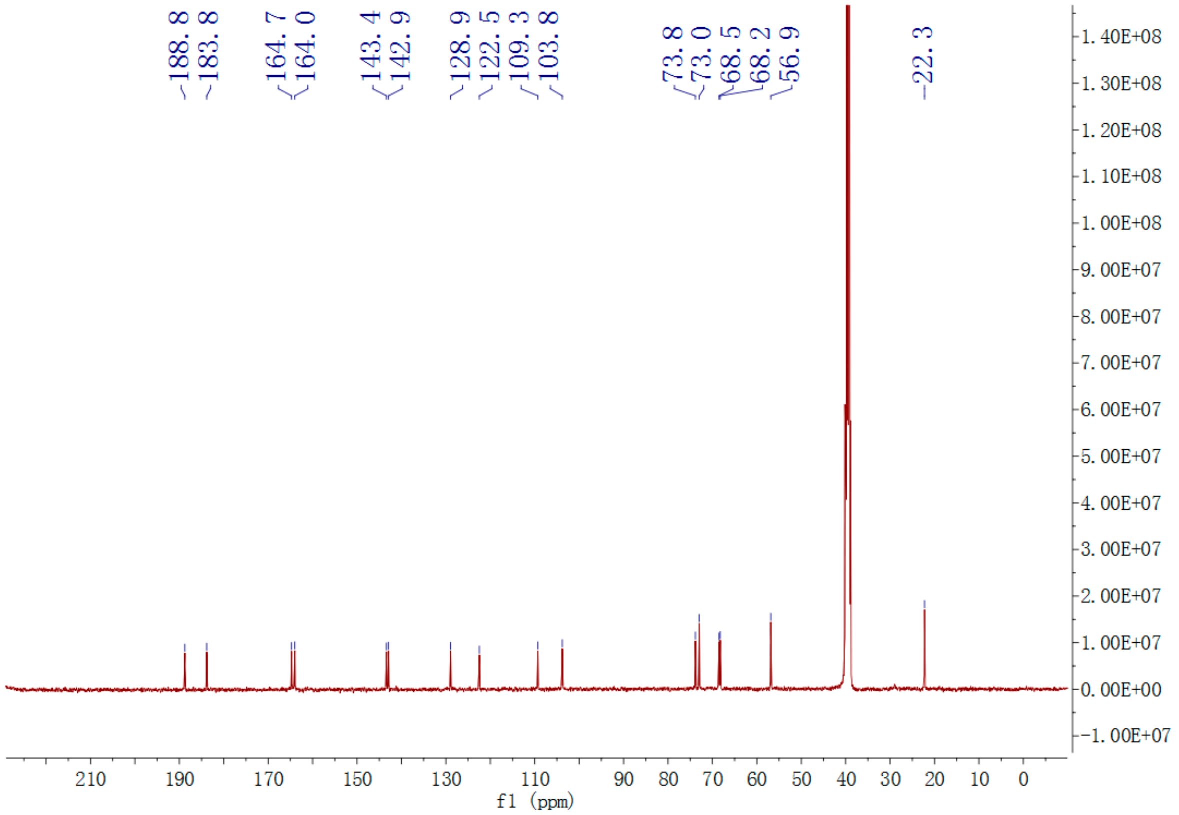

Figure S45. HSQC of compound 6, measured at 400 MHz (^{1}H) and 100 MHz (^{13}C) (DMSO- *d*6).

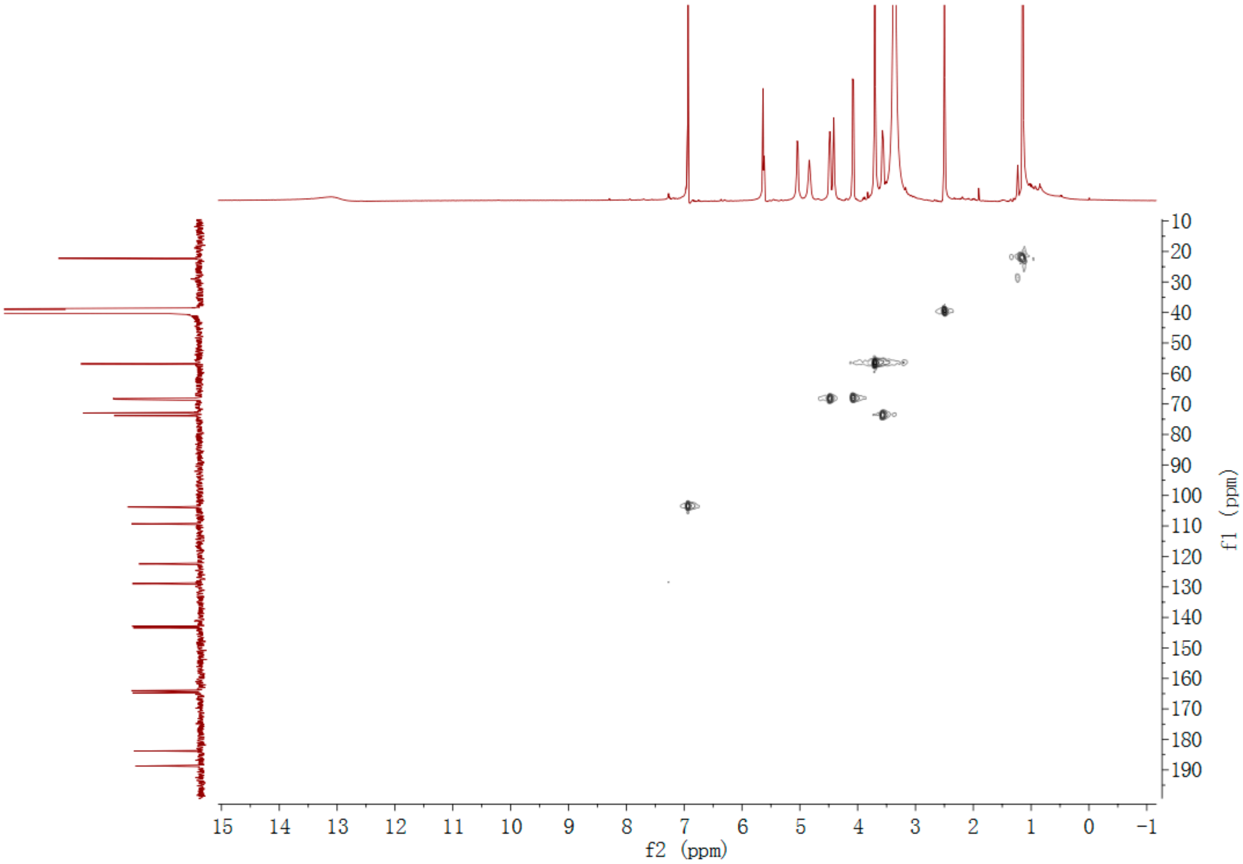

Figure S46. HMBC of compound 6, measured at 400 MHz (^{1}H) and 100 MHz (^{13}C) (DMSO- *d*6).

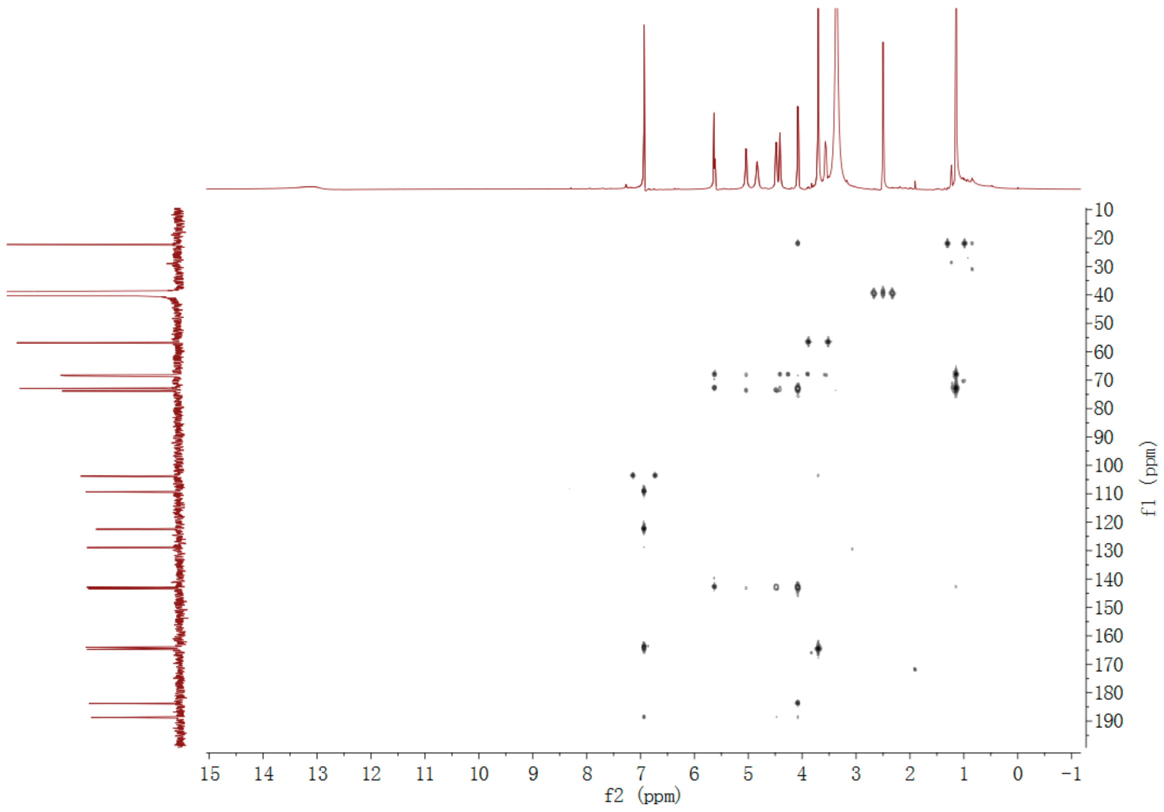

Figure S47. NOESY of compound 6, measured at 400 MHz (^{1}H) and 100 MHz (^{13}C) (DMSO- *d*6).

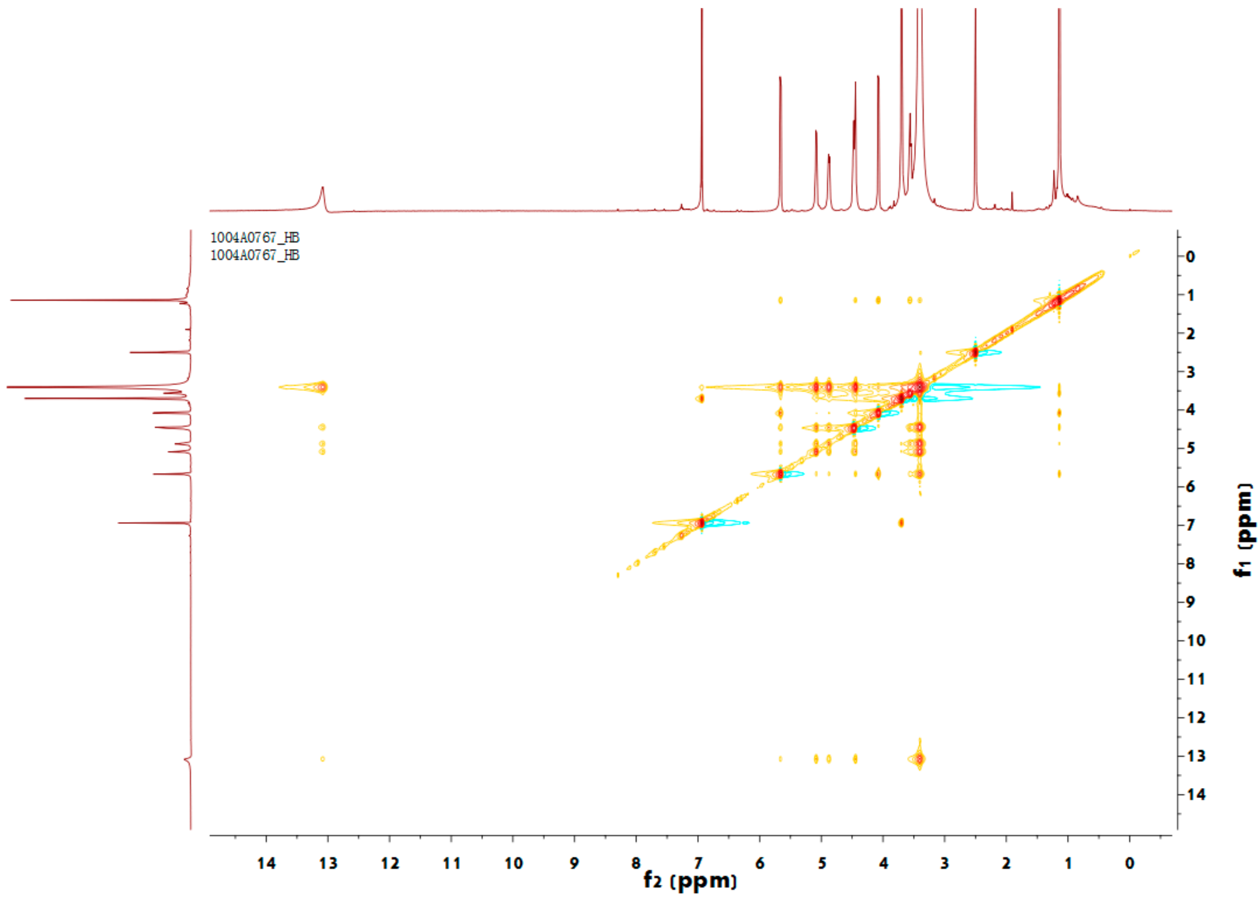

Figure S48. CD spectra of compound 6 in acetonitrile solution.

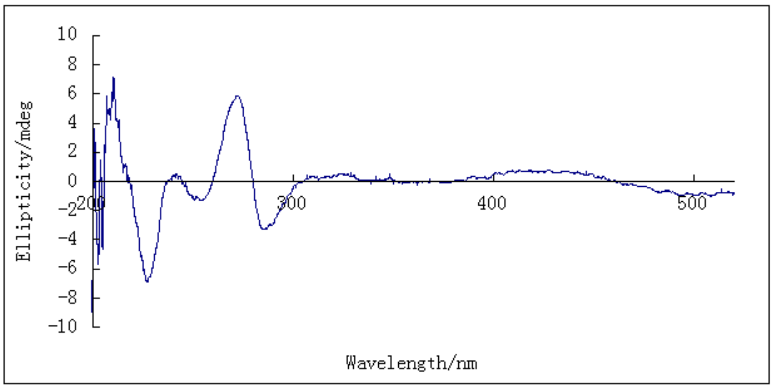

Figure S49. HR-ESI-TOF-MS spectra of compound 6.

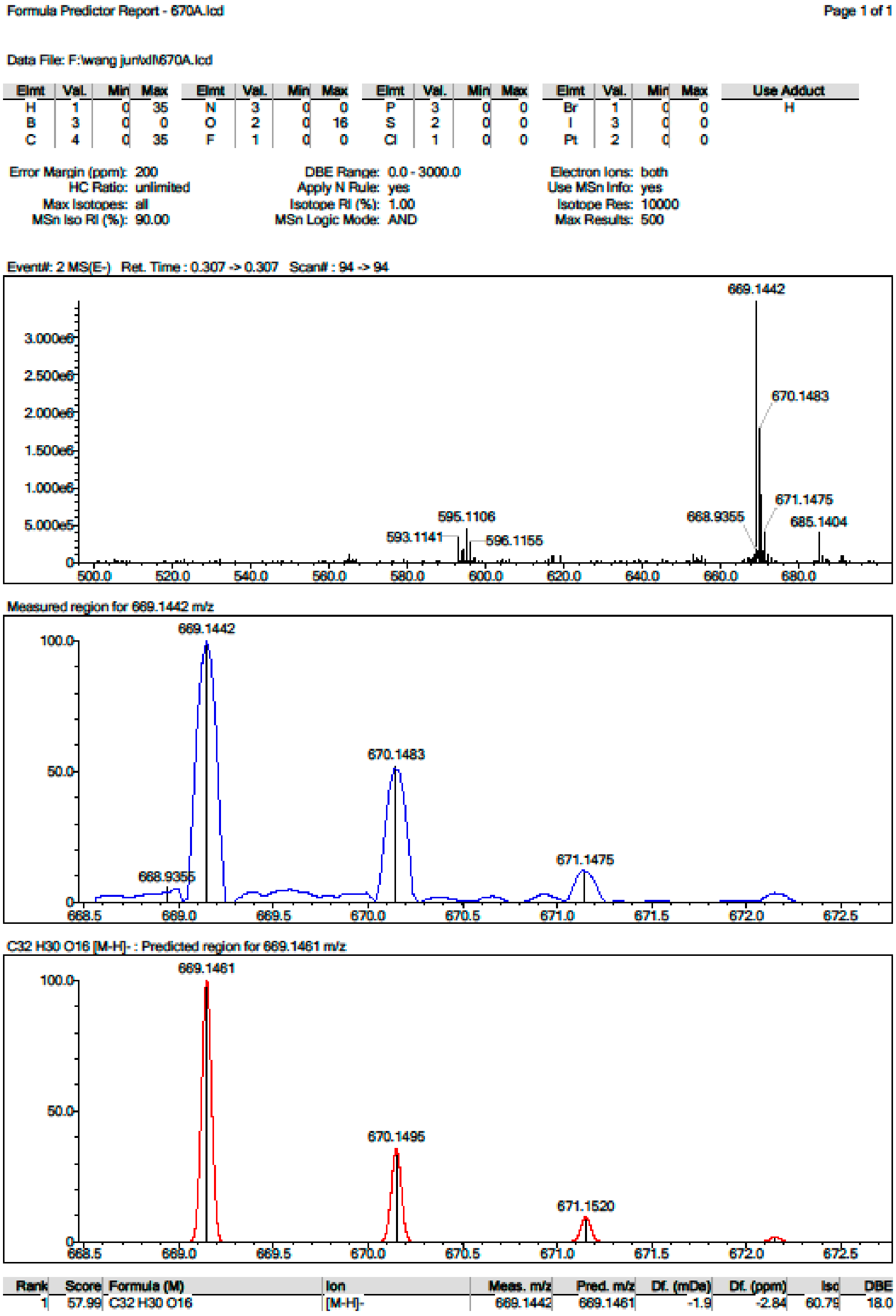

Figure S50. ^{1}H-NMR spectra of compound 7, measured at 400 MHz (DMSO- *d*6).

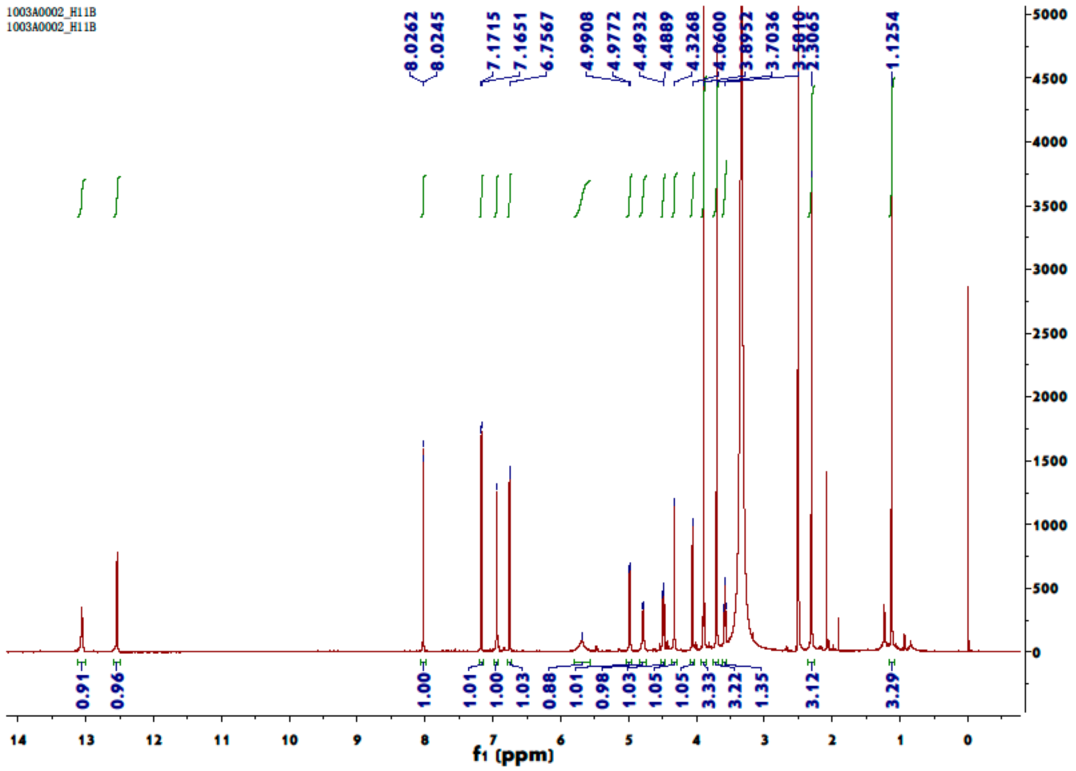

Figure S51. ^{13}C-NMR spectra of compound 7, measured at 100 MHz (DMSO- *d*6).

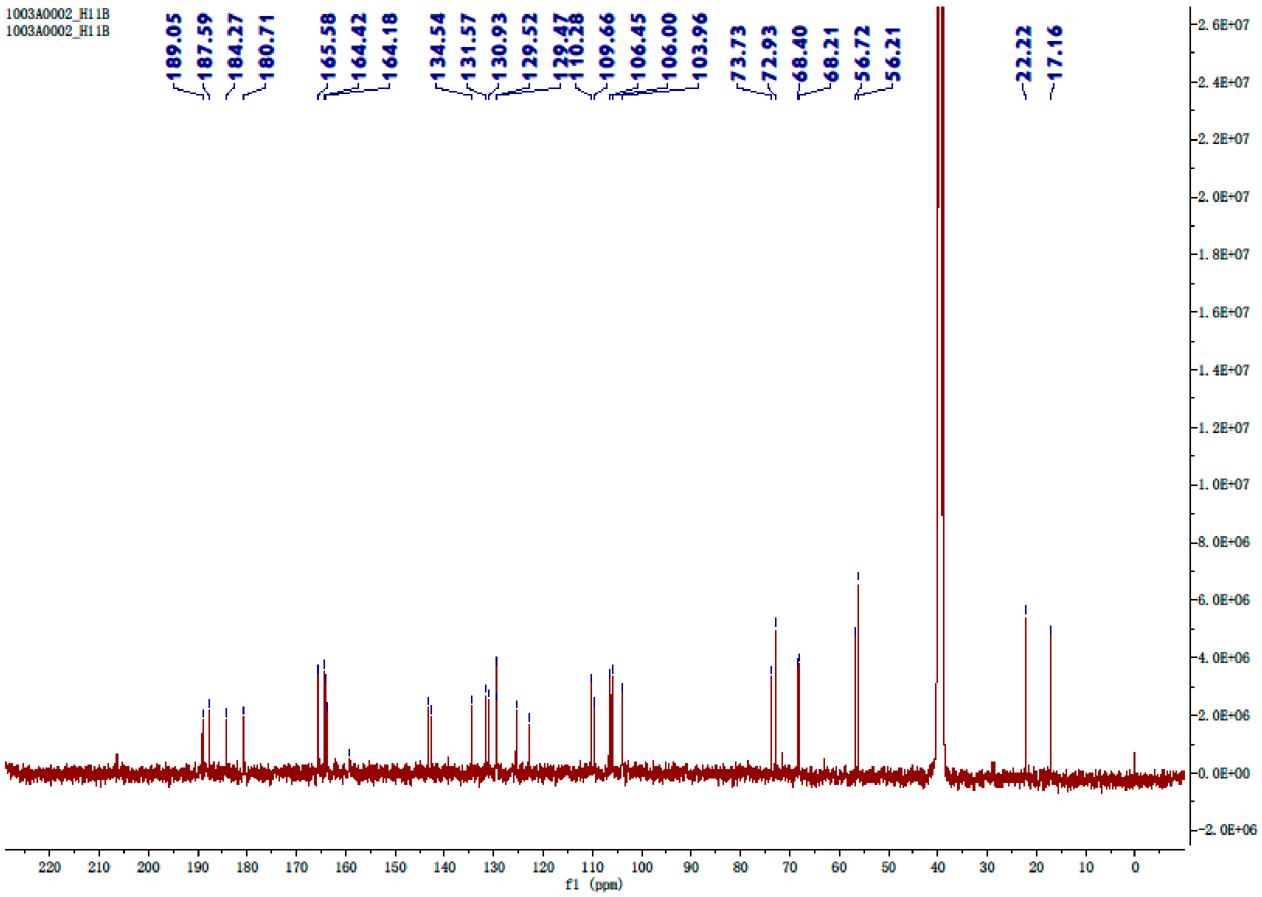

Figure S52. ^{1}H-^{1}H COSY of compound 7, measured at 400 MHz (DMSO- *d*6).

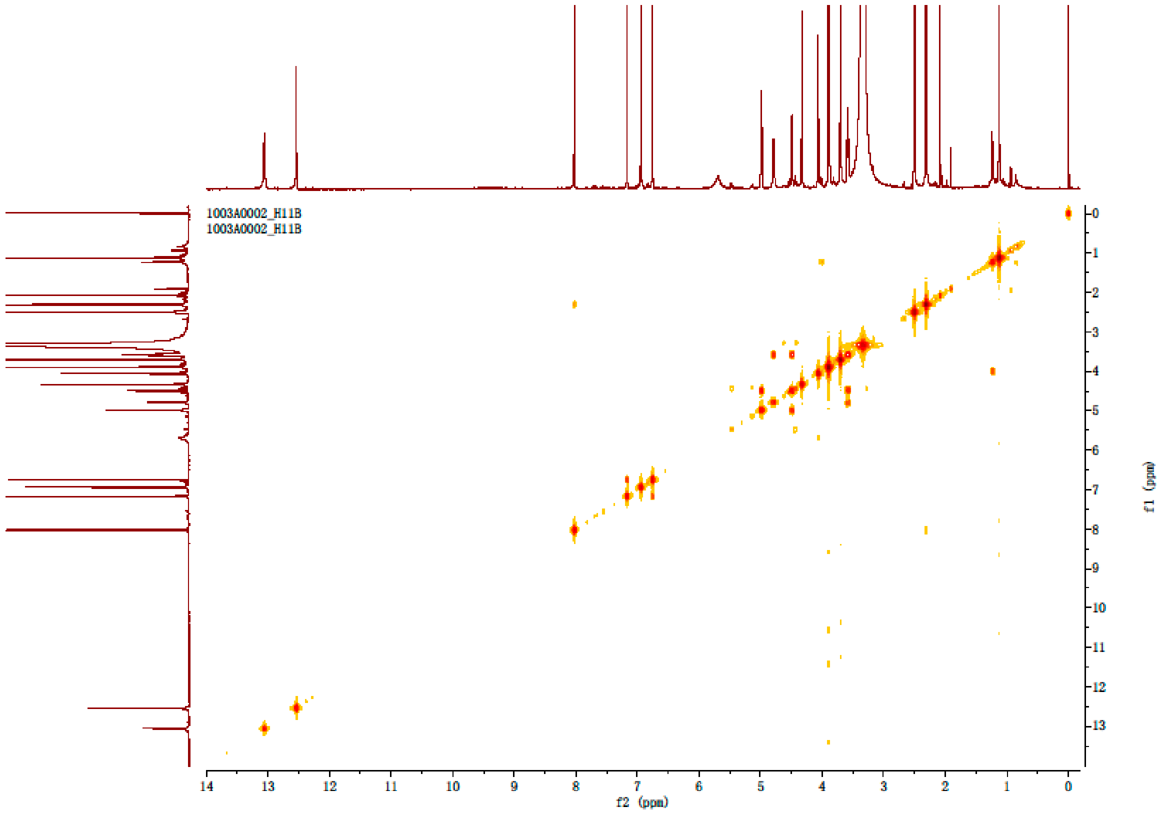

Figure S53. HSQC of compound 7, measured at 400 MHz (^{1}H) and 100 MHz (^{13}C) (DMSO- *d*6).

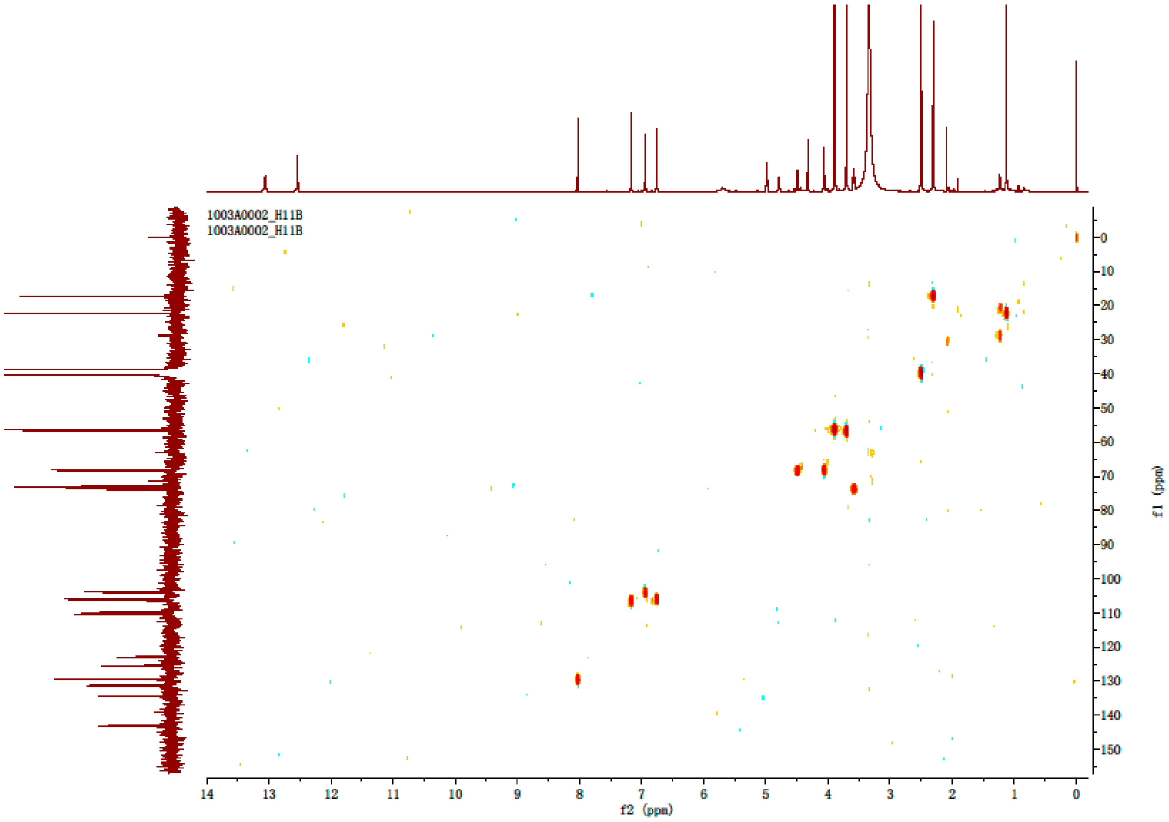

Figure S54. HMBC of compound 7, measured at 400 MHz (^{1}H) and 100 MHz (^{13}C) (DMSO- *d*6).

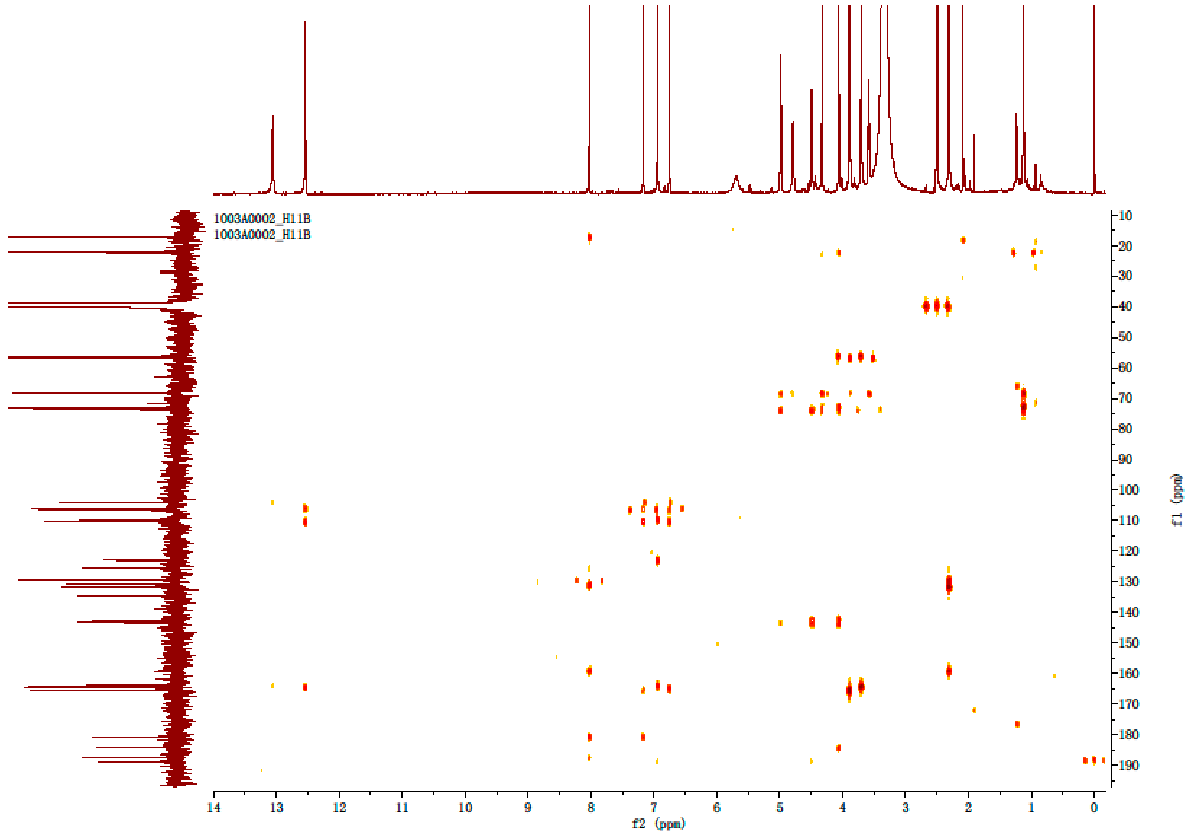

Figure S55. NOESY of compound 7, measured at 400 MHz (^{1}H) and 100 MHz (^{13}C) (DMSO- *d*6).

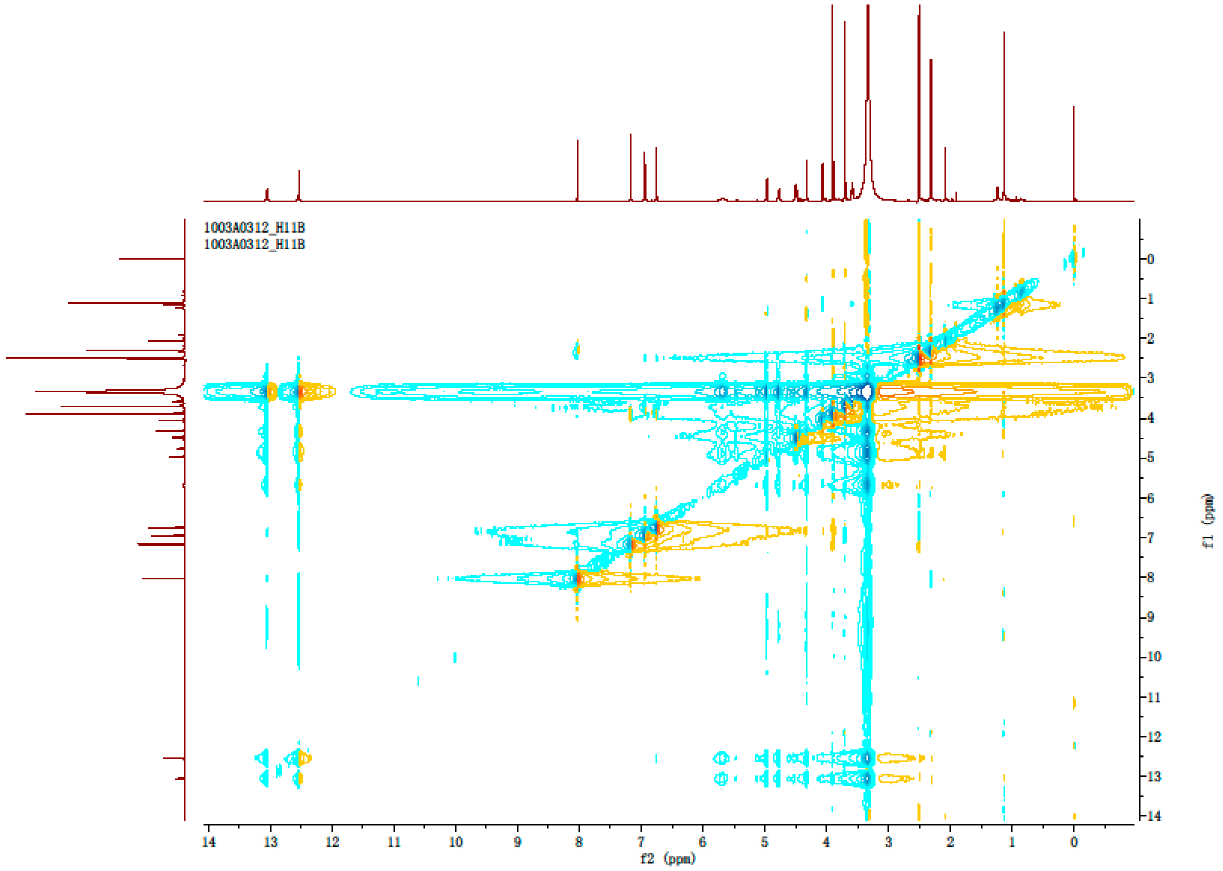

Figure S56. HR-ESI-TOF-MS spectra of compound 7.

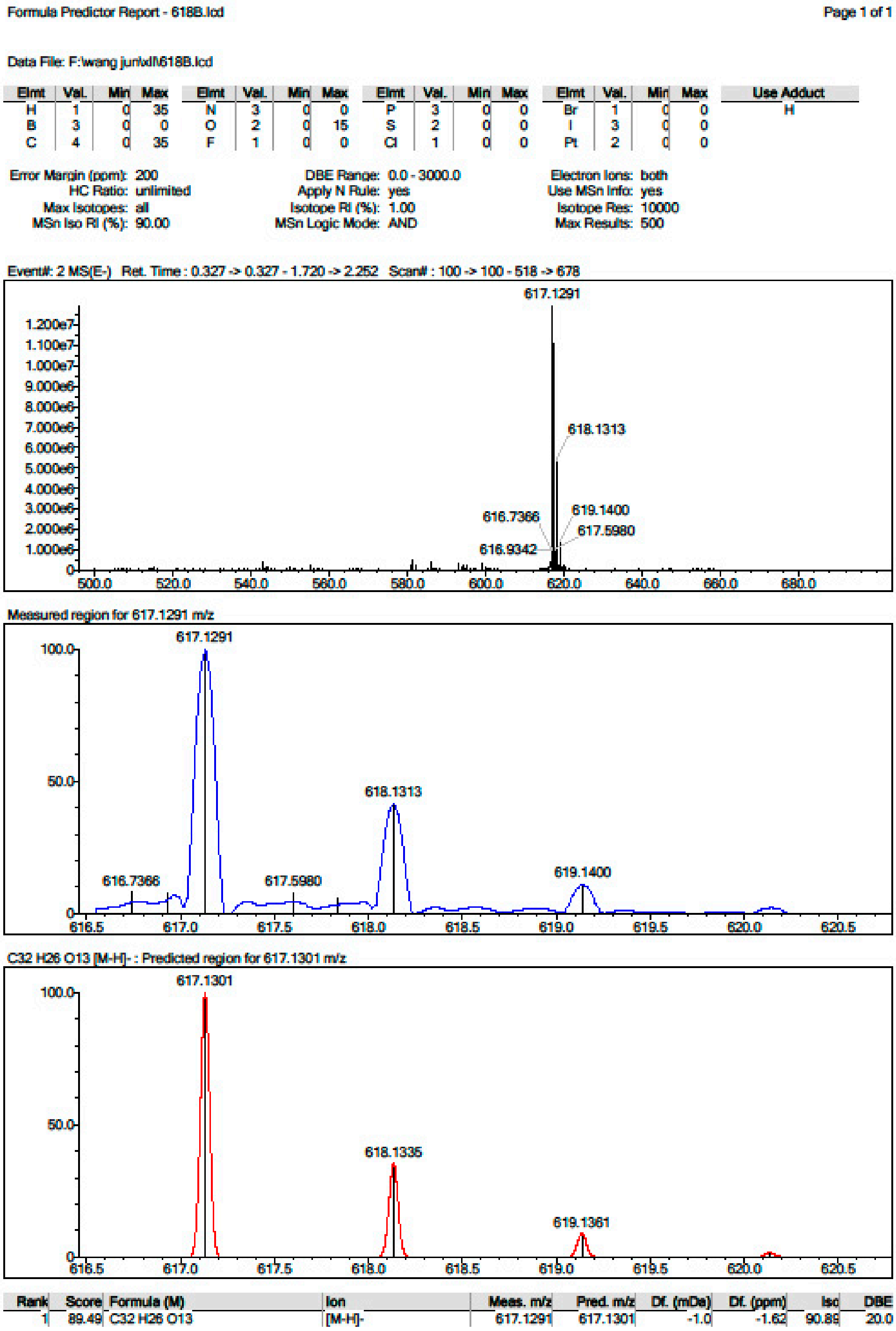

Figure S57. CD spectra of compound 7 in acetonitrile solution.

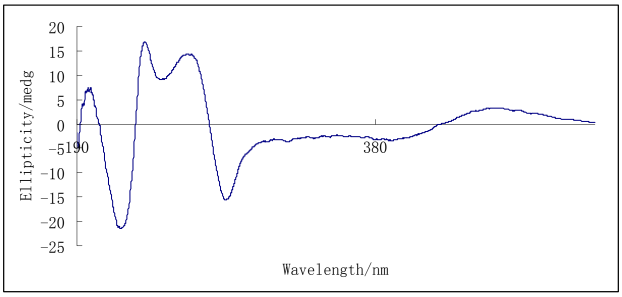

Figure S58. ^{1}H-NMR spectra of compound 8, measured at 400 MHz (DMSO- *d*6).

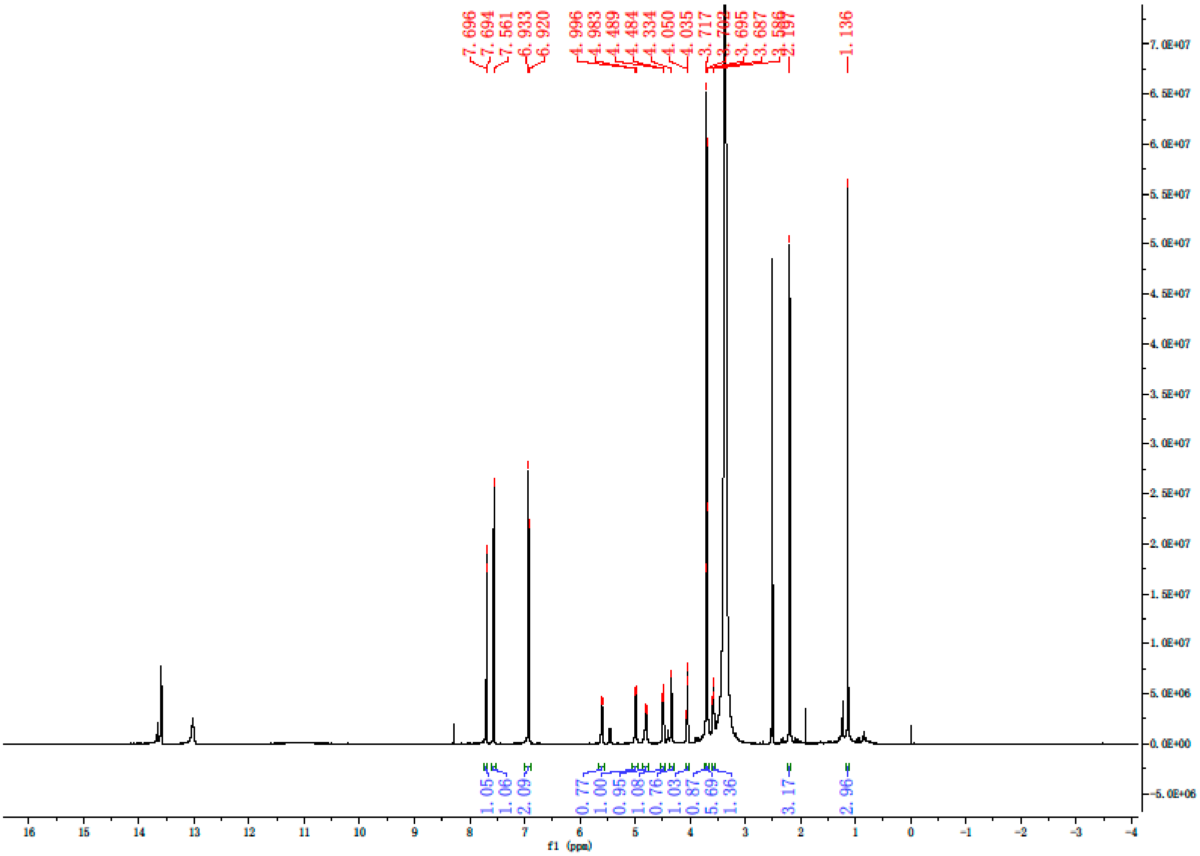

Figure S59. ^{13}C-NMR spectra of compound 8, measured at 100 MHz (DMSO- *d*6).

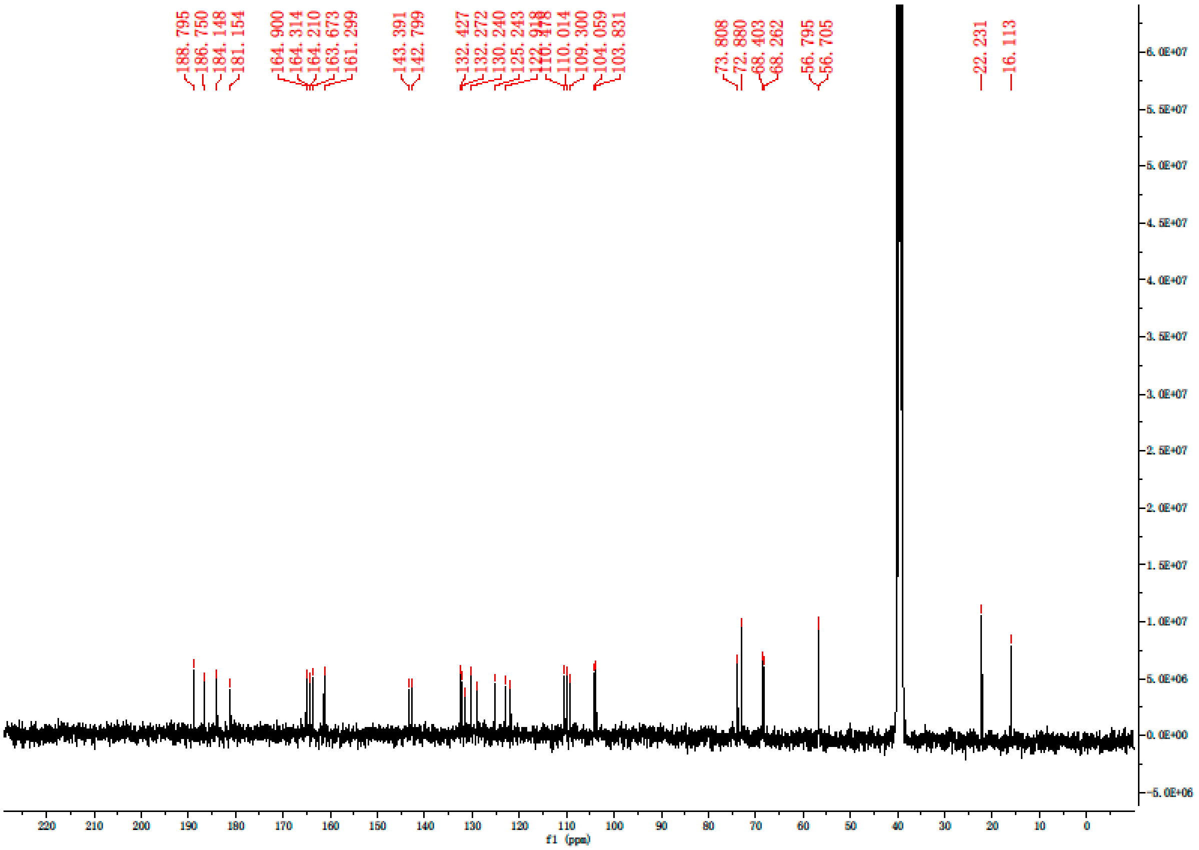

Figure S60. ^{1}H-^{1}H COSY of compound 8, measured at 400 MHz (DMSO- *d*6).

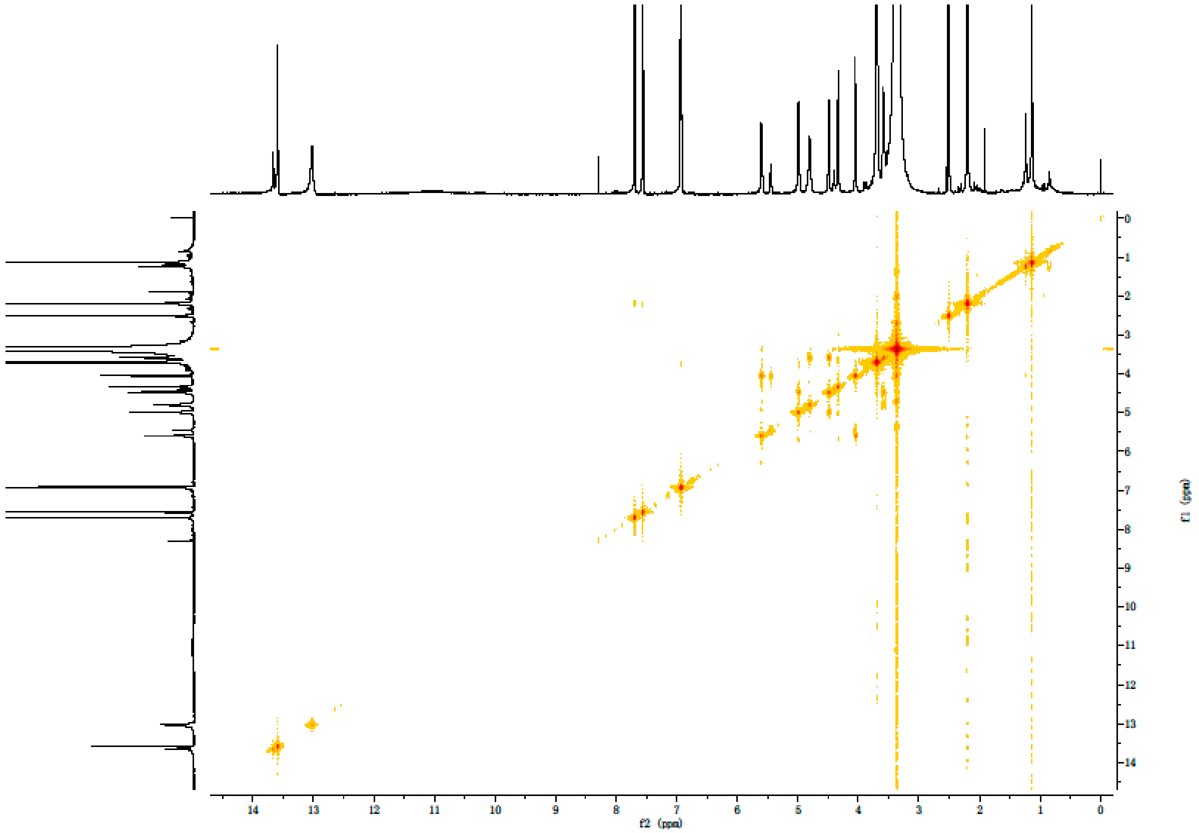

Figure S61. HSQC of compound 8, measured at 400 MHz (^{1}H) and 100 MHz (^{13}C) (DMSO- *d*6).

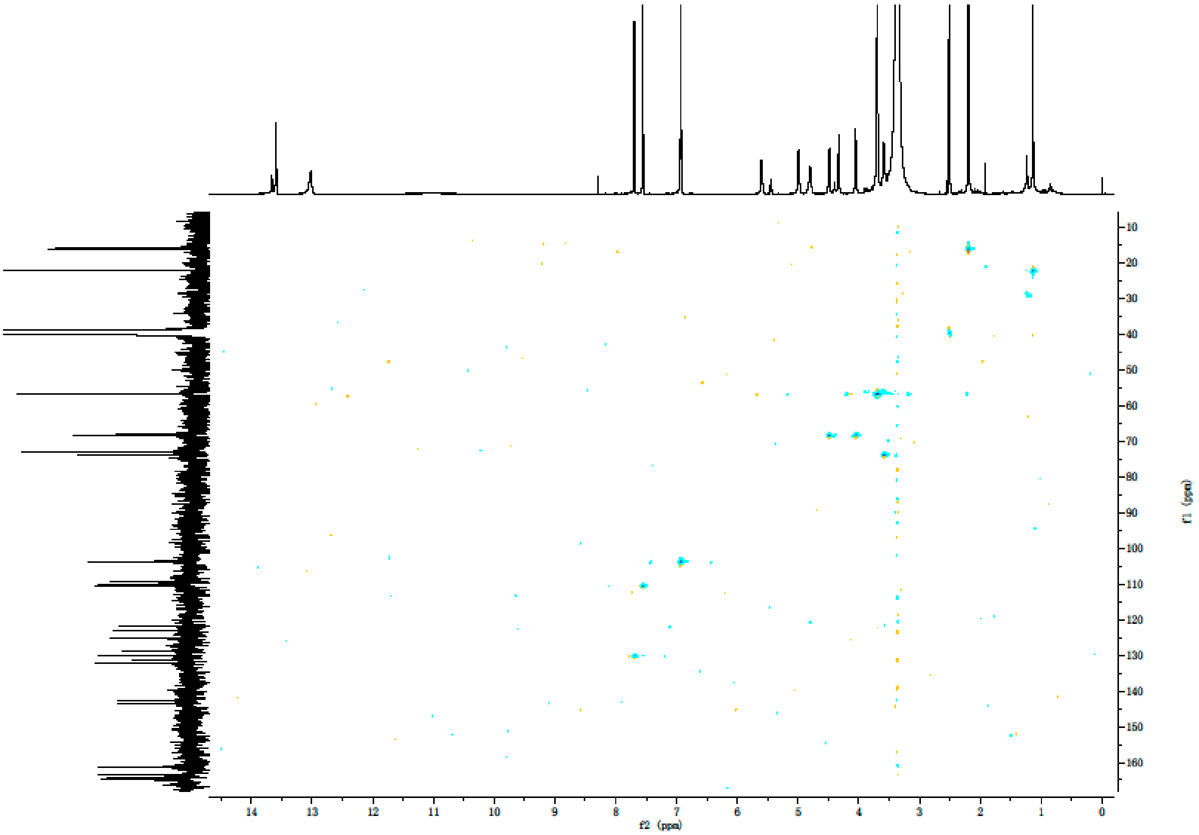

Figure S62. HMBC of compound 8, measured at 400 MHz (^{1}H) and 100 MHz (^{13}C) (DMSO- *d*6).

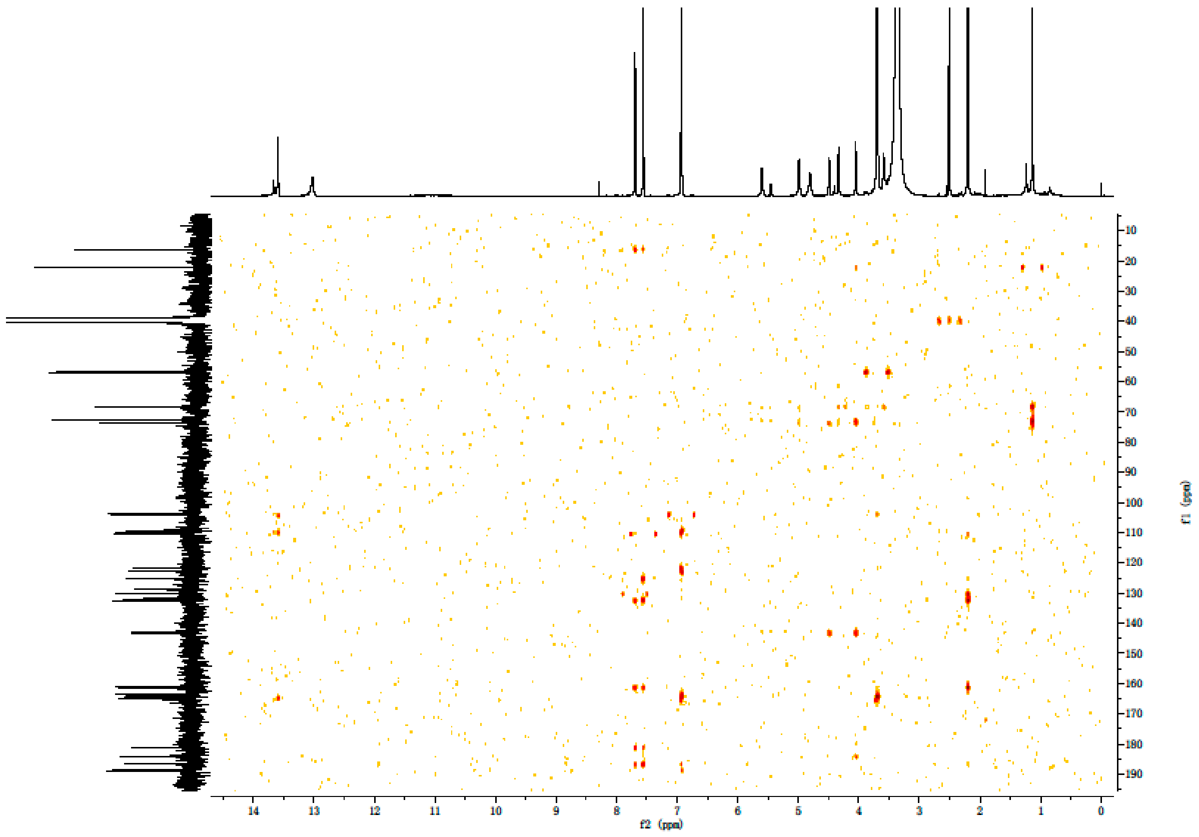

Figure S63. NOESY of compound 8, measured at 400 MHz (^{1}H) and 100 MHz (^{13}C) (DMSO- *d*6).

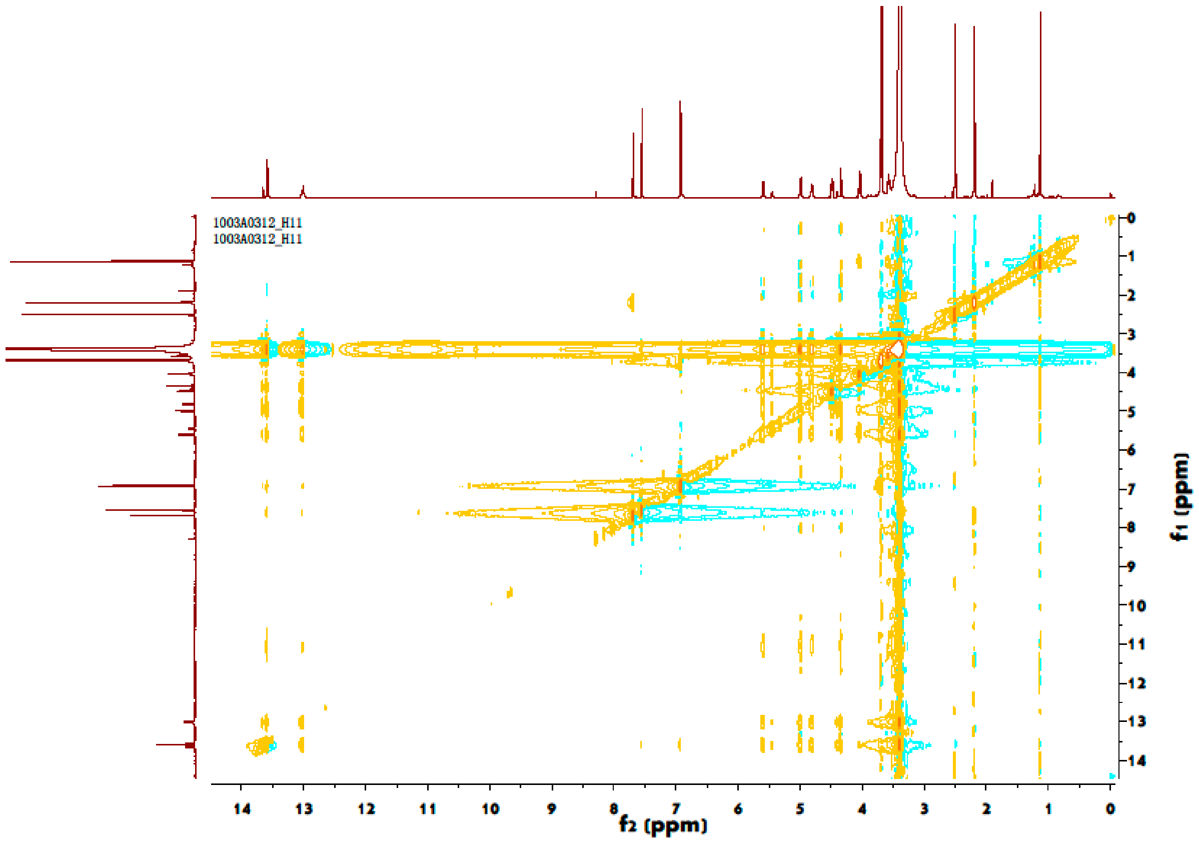

Figure S64. HR-ESI-TOF-MS spectra of compound 8.

Formula Predictor Report - h11.lcd Page 1 of 1

Data File: F:\wang jun\xl\h11.lcd

Elmt	Val.	Min	Max	Elmt	Val.	Min	Max	Elmt	Val.	Min	Max	Elmt	Val.	Min	Max	Use Adduct
H	1	0	35	N	3	0	0	P	3	0	0	Br	1	0	0	H
B	3	0	0	O	2	0	15	S	2	0	0	I	3	0	0	
C	4	0	35	F	1	0	0	Cl	1	0	0	Pt	2	0	0	

Error Margin (ppm):	200	DBE Range:	0.0 - 3000.0	Electron Ions:	both
HC Ratio:	unlimited	Apply N Rule:	yes	Use MSn Info:	yes
Max Isotopes:	all	Isotope RI (%):	1.00	Isotope Res:	10000
MSn Iso RI (%):	90.00	MSn Logic Mode:	AND	Max Results:	500

Event#: 2 MS(E-) Ret. Time : 0.293 -> 0.293 - 0.107 -> 0.181 Scan# : 90 -> 90 - 34 -> 56

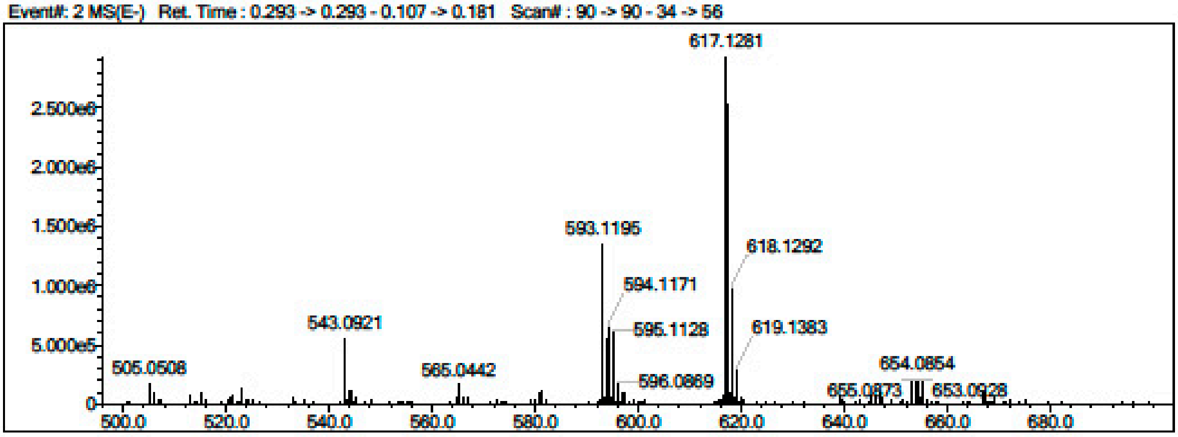

Measured region for 617.1281 m/z

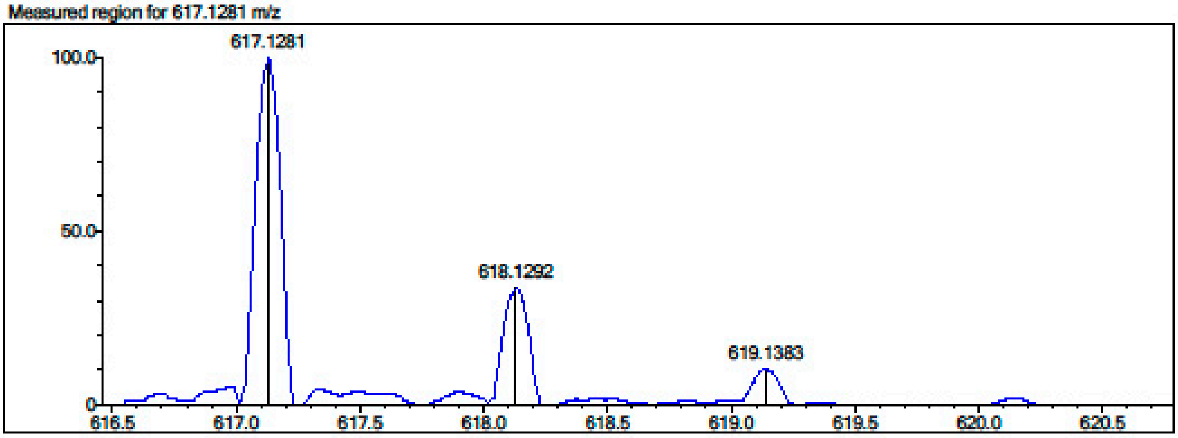

C32 H26 O13 [M-H]- : Predicted region for 617.1301 m/z

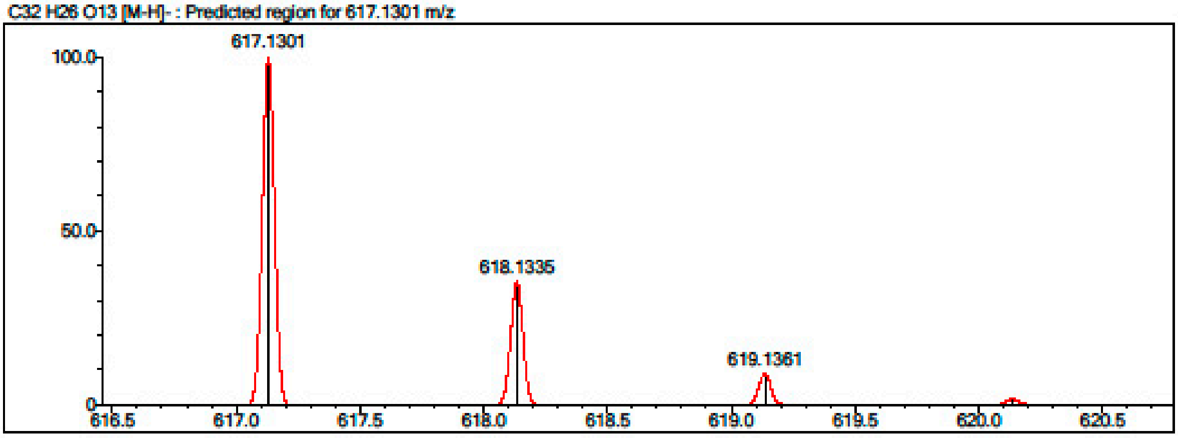

Rank	Score	Formula (M)	Ion	Meas. m/z	Pred. m/z	Df. (mDa)	Df. (ppm)	Iso	DBE
1	94.40	C32 H26 O13	[M-H]-	617.1281	617.1301	-2.0	-3.24	100.00	20.0

Figure S65. ^{1}H-NMR spectra of compound 9, measured at 400 MHz (DMSO- *d*6).

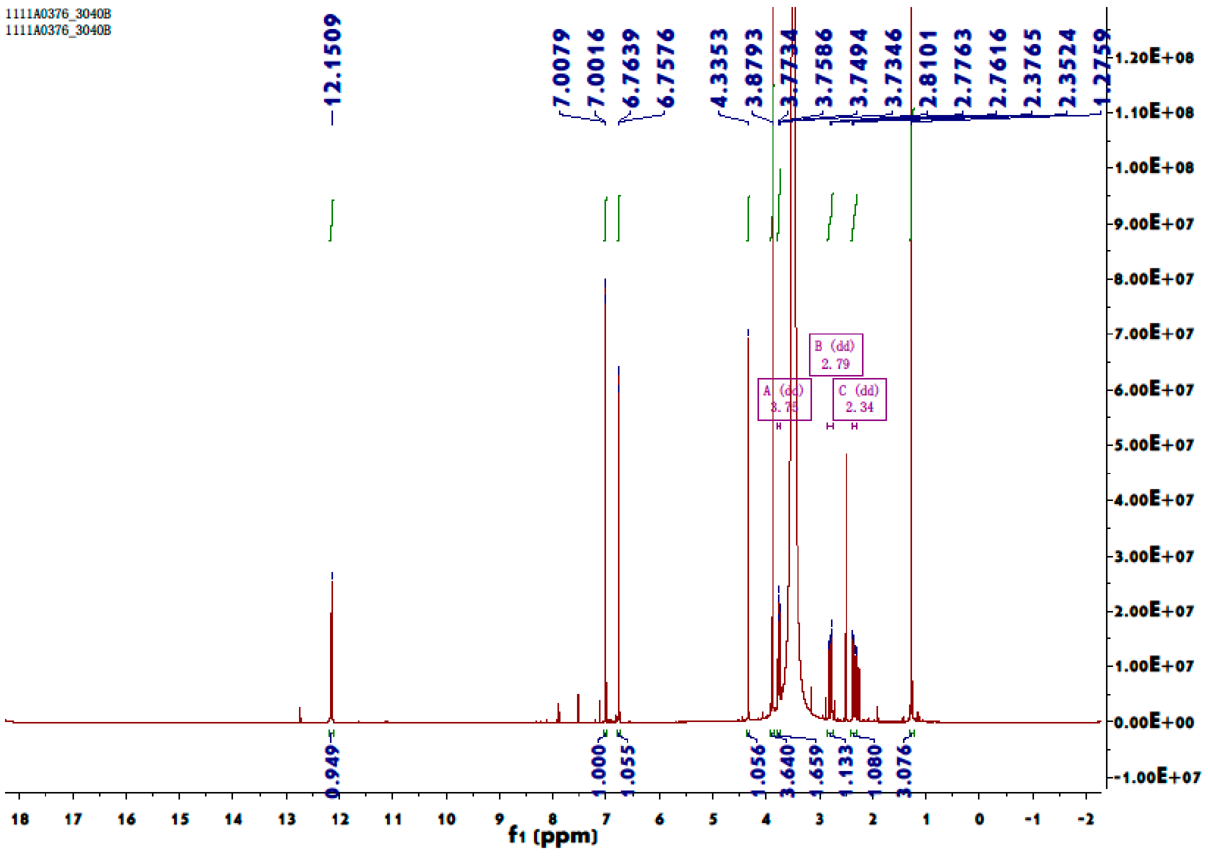

Figure S66. ^{13}C-NMR spectra of compound 9, measured at 100 MHz (DMSO- *d*6).

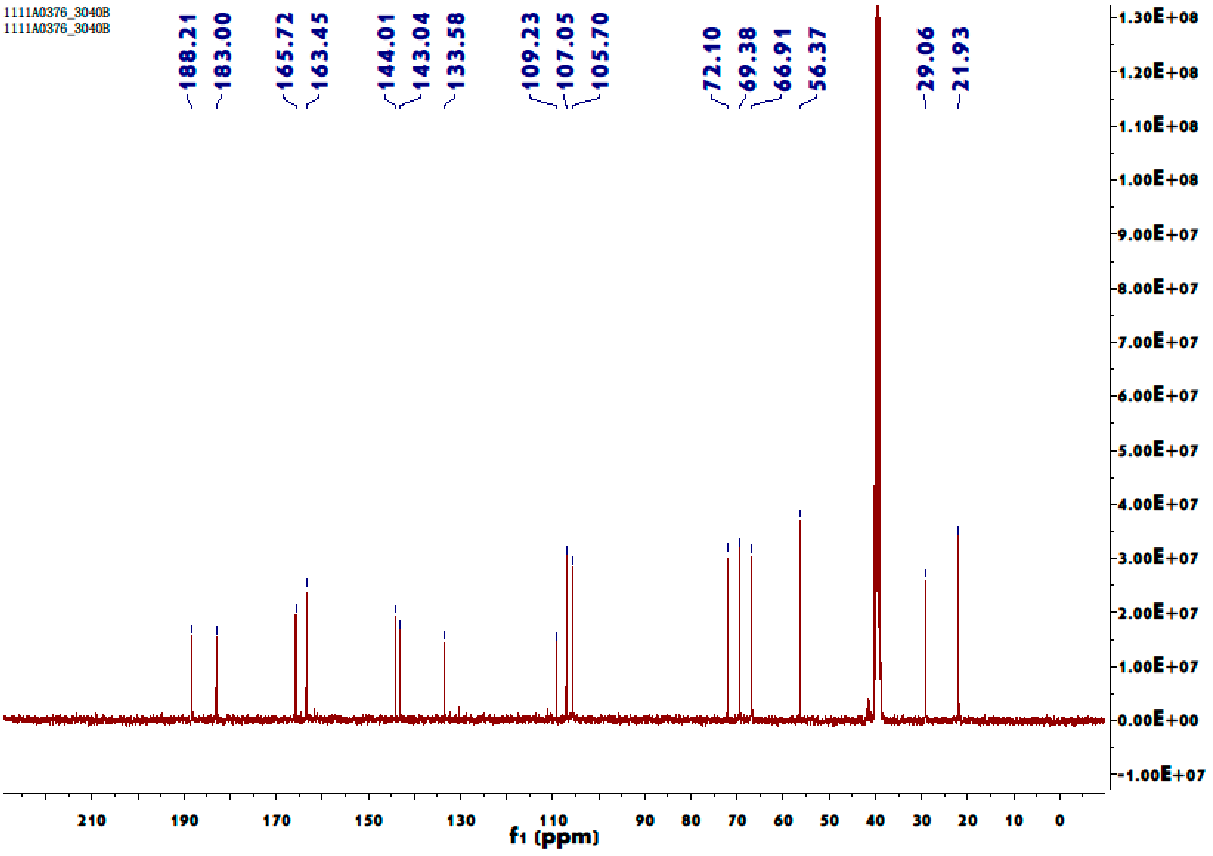

Figure S67. DEPT-90 ^{13}C-NMR data of compound 9, measured at 100 MHz (DMSO- *d*6).

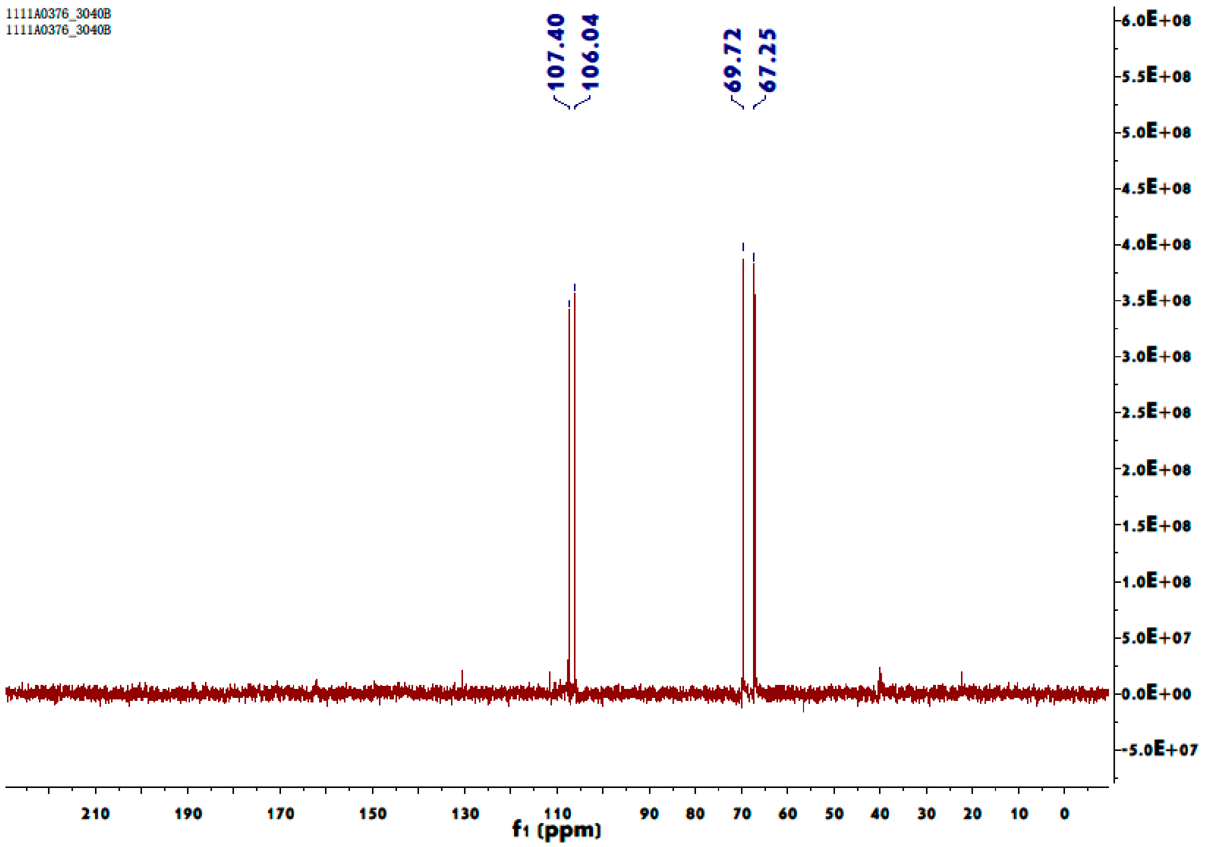

Figure 68. DEPT-135 ^{13}C-NMR data of compound 9, measured at 100 MHz (DMSO- *d*6).

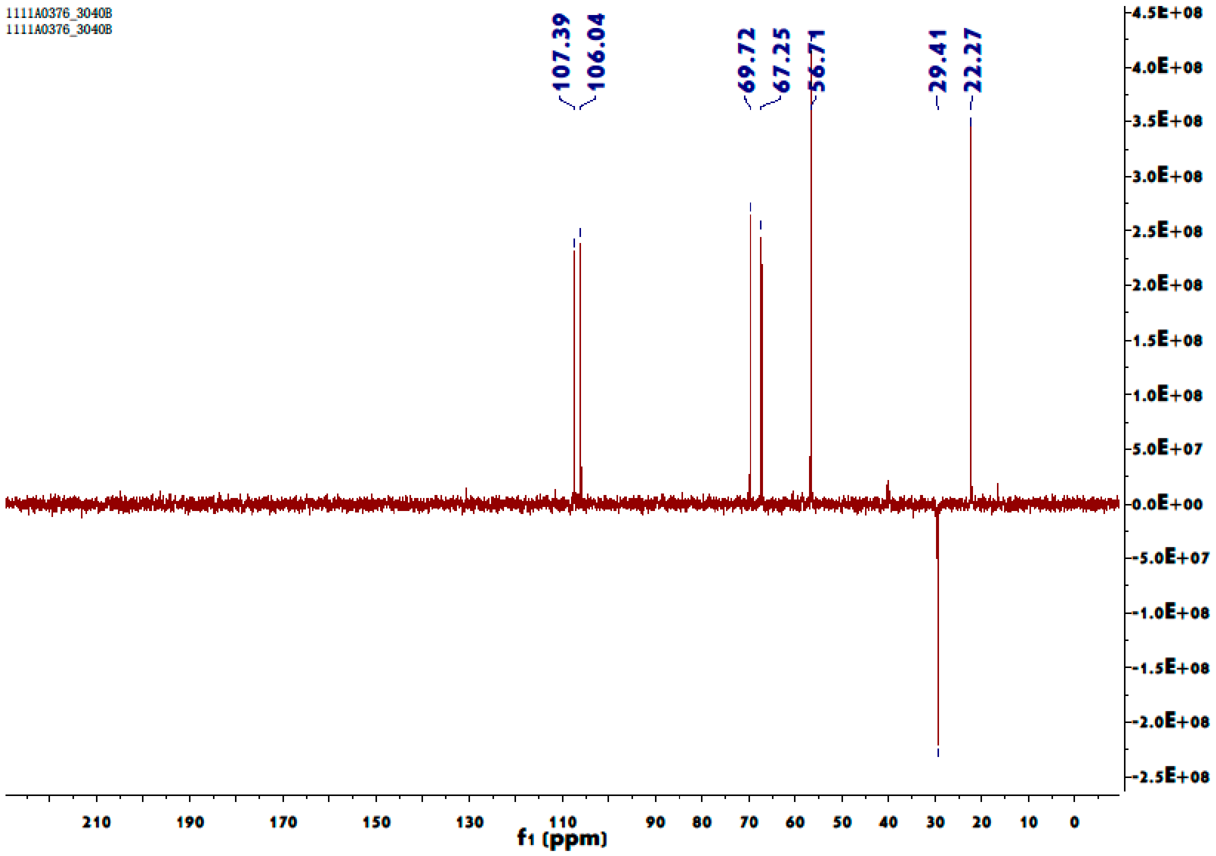

Figure S69. ^{1}H-^{1}H COSY of compound 9, measured at 400 MHz (DMSO- *d*6).

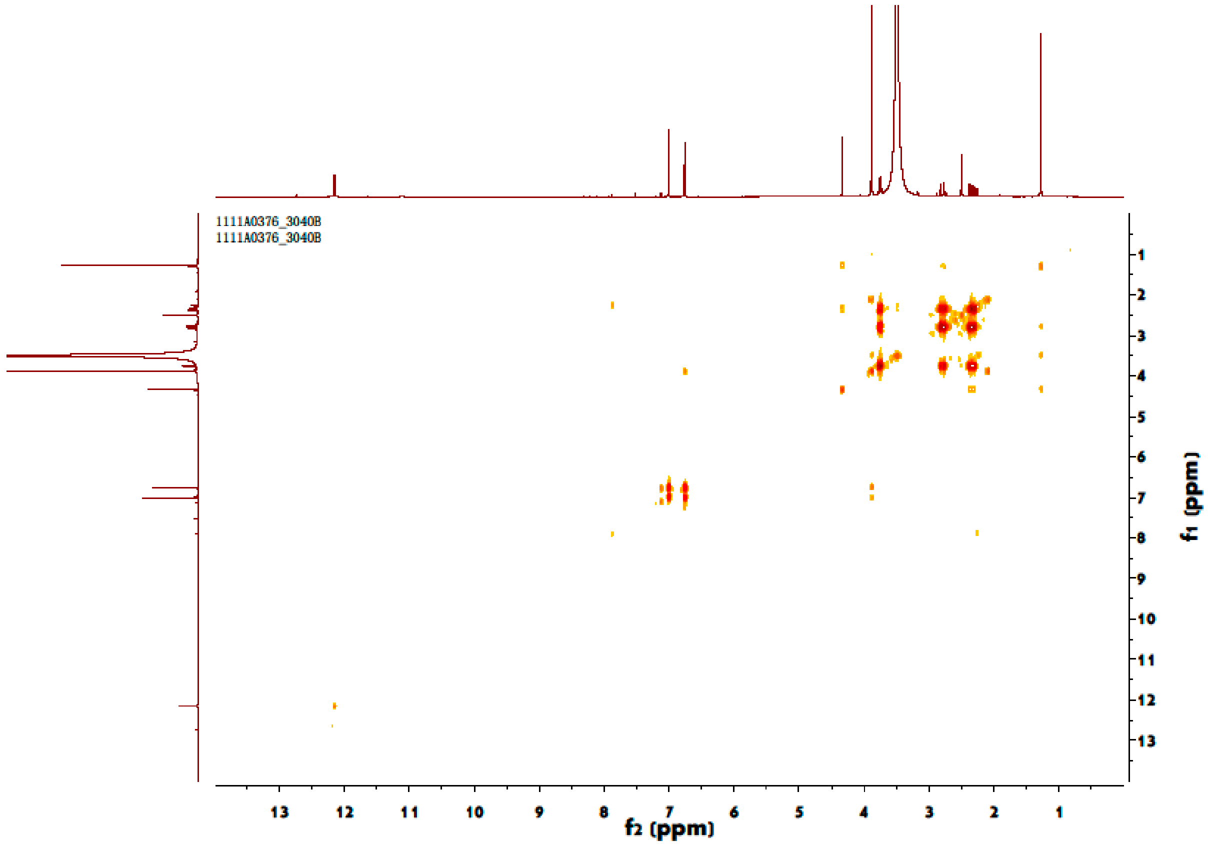

Figure S70. HSQC of compound 9, measured at 400 MHz (^{1}H) and 100 MHz (^{13}C) (DMSO- *d*6).

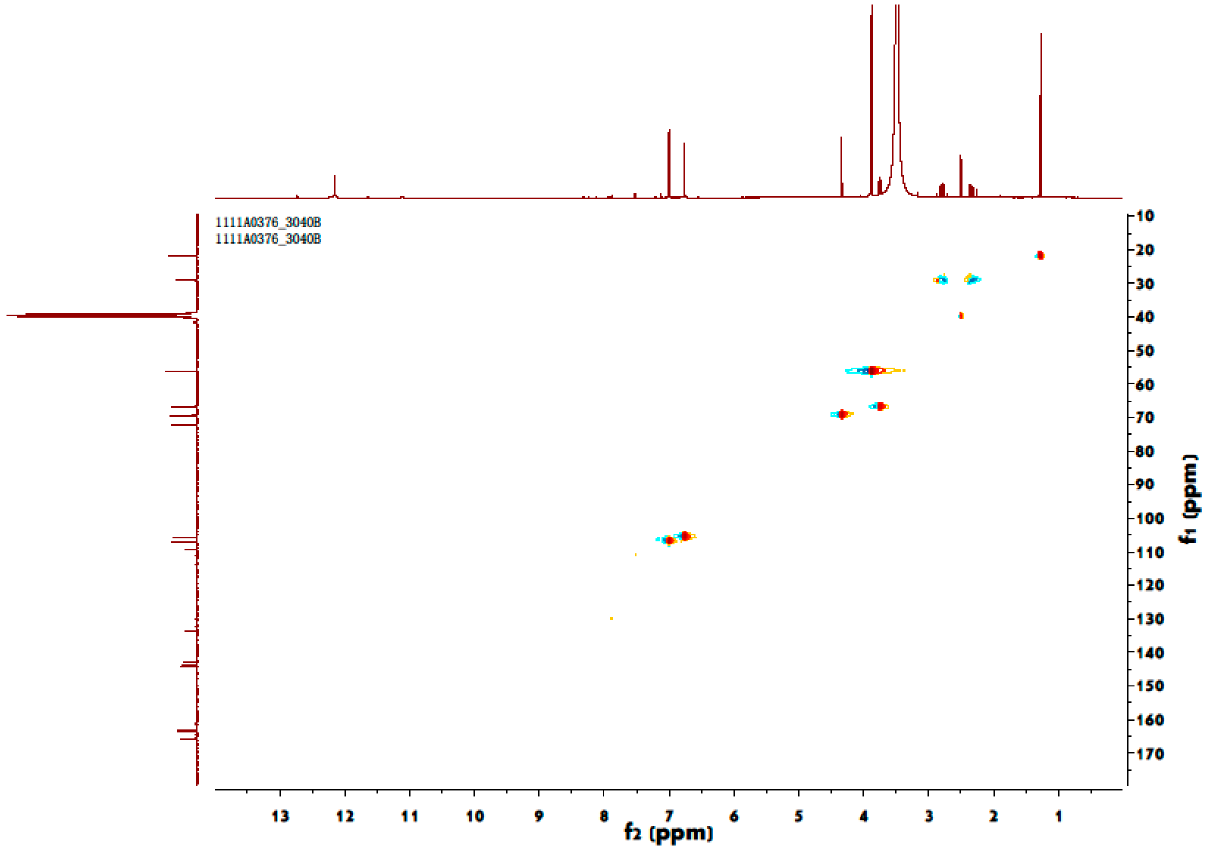

Figure S71. HMBC of compound 9, measured at 400 MHz (^{1}H) and 100 MHz (^{13}C) (DMSO- *d*6).

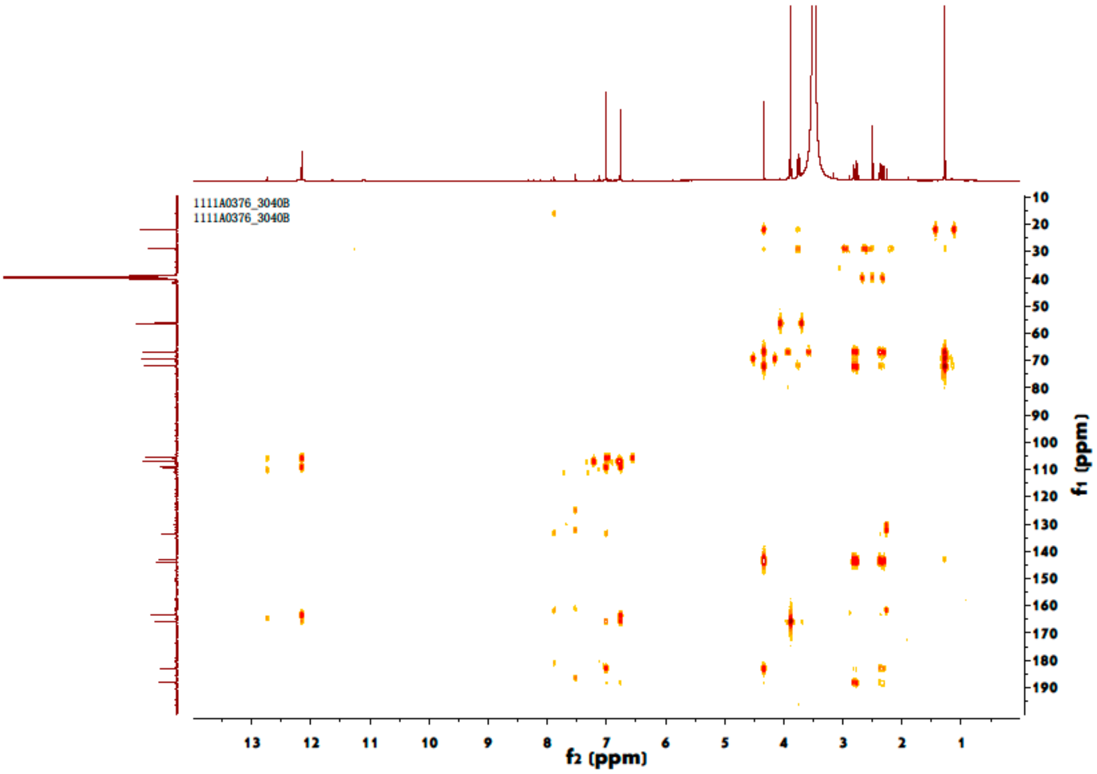

Figure S72. ESI -MS spectra of compound 9.

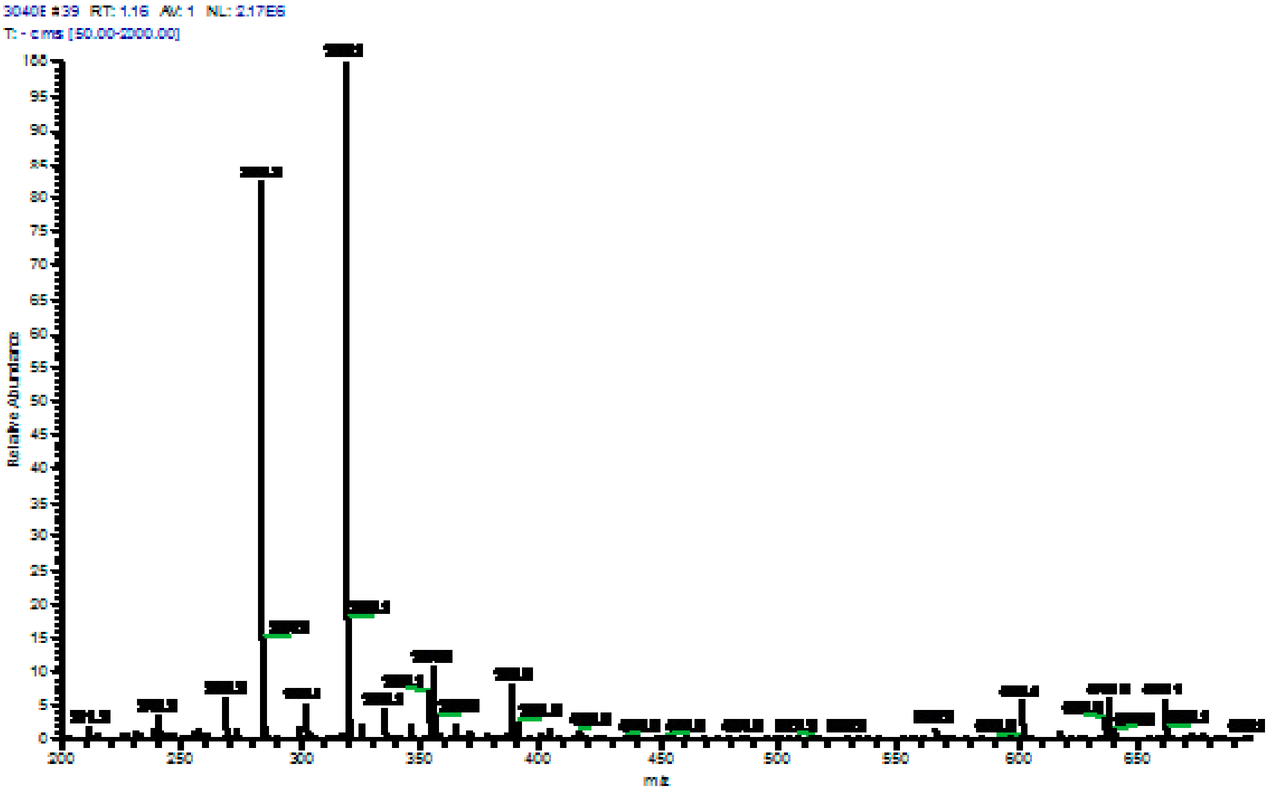

Figure S73. ^{1}H-NMR spectra of compound 10, measured at 400 MHz (DMSO- *d*6).

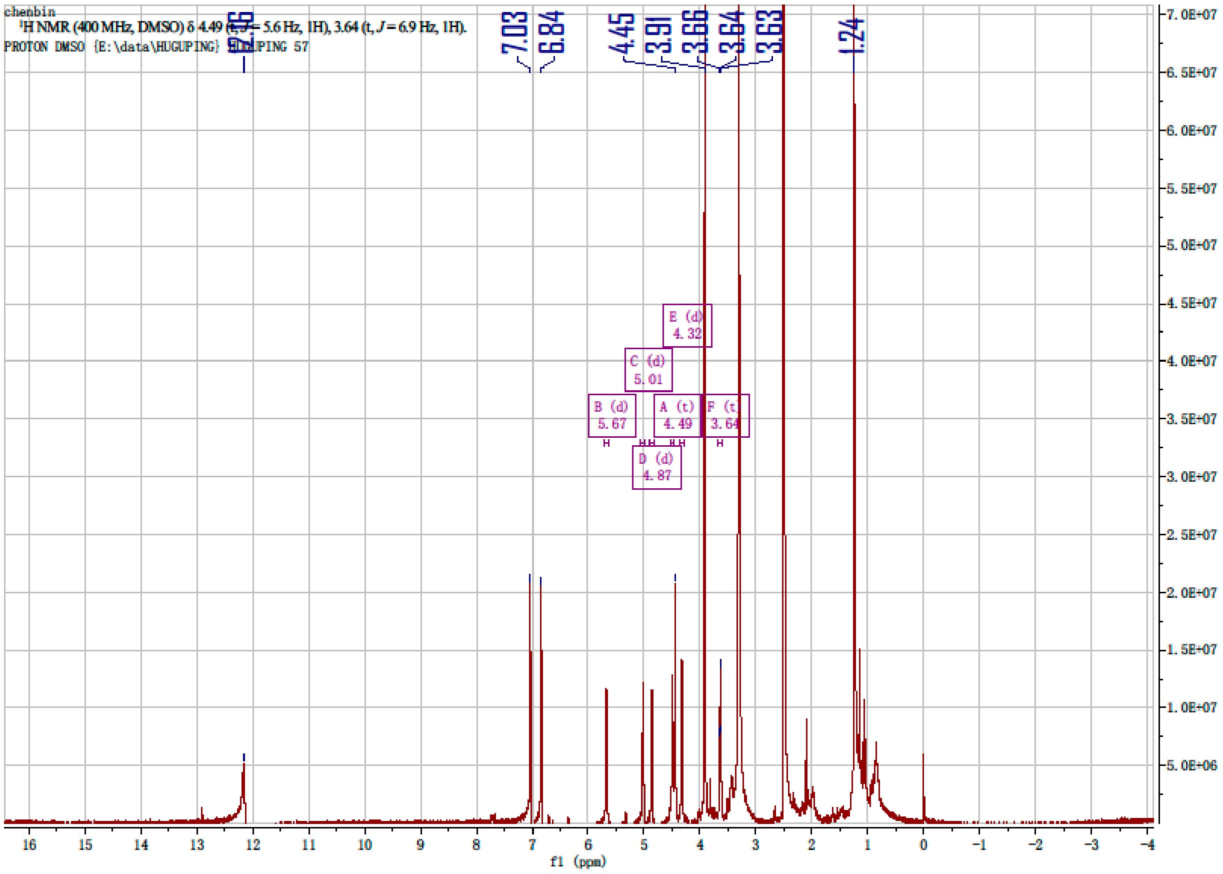

Figure S74. ^{13}C-NMR spectra of compound 10, measured at 100 MHz (DMSO- *d*6).

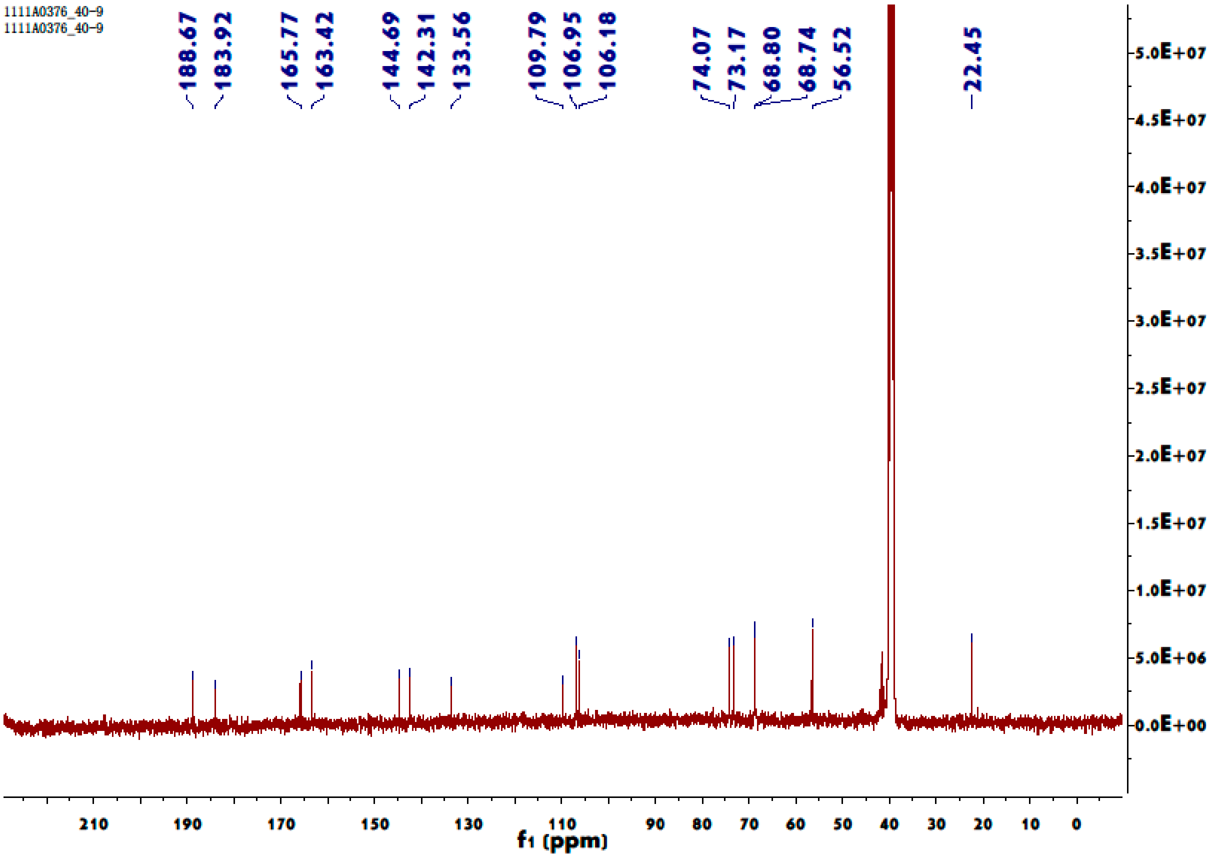

Figure S75. DEPT-135 ^{13}C-NMR data of compound 10, measured at 100 MHz (DMSO- *d*6).

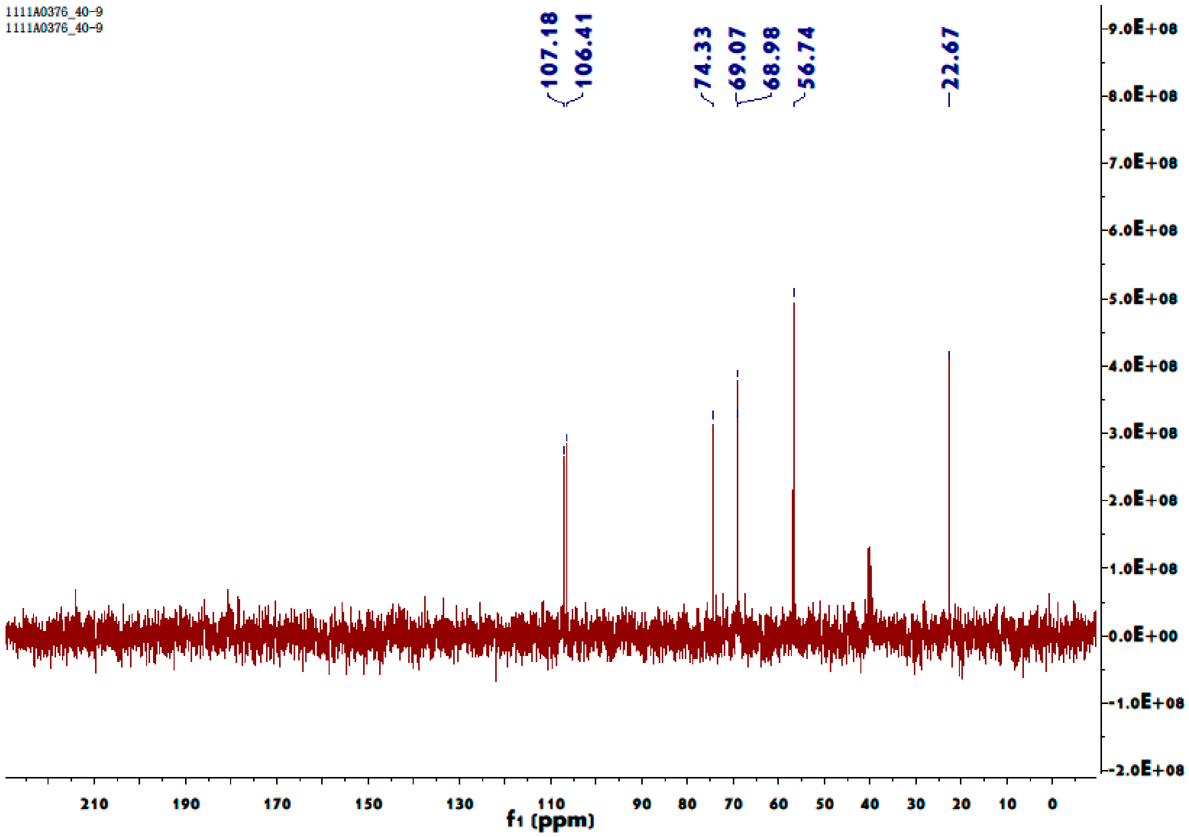

Figure S76. ^{1}H-^{1}H COSY of compound 10, measured at 400 MHz (DMSO- *d*6).

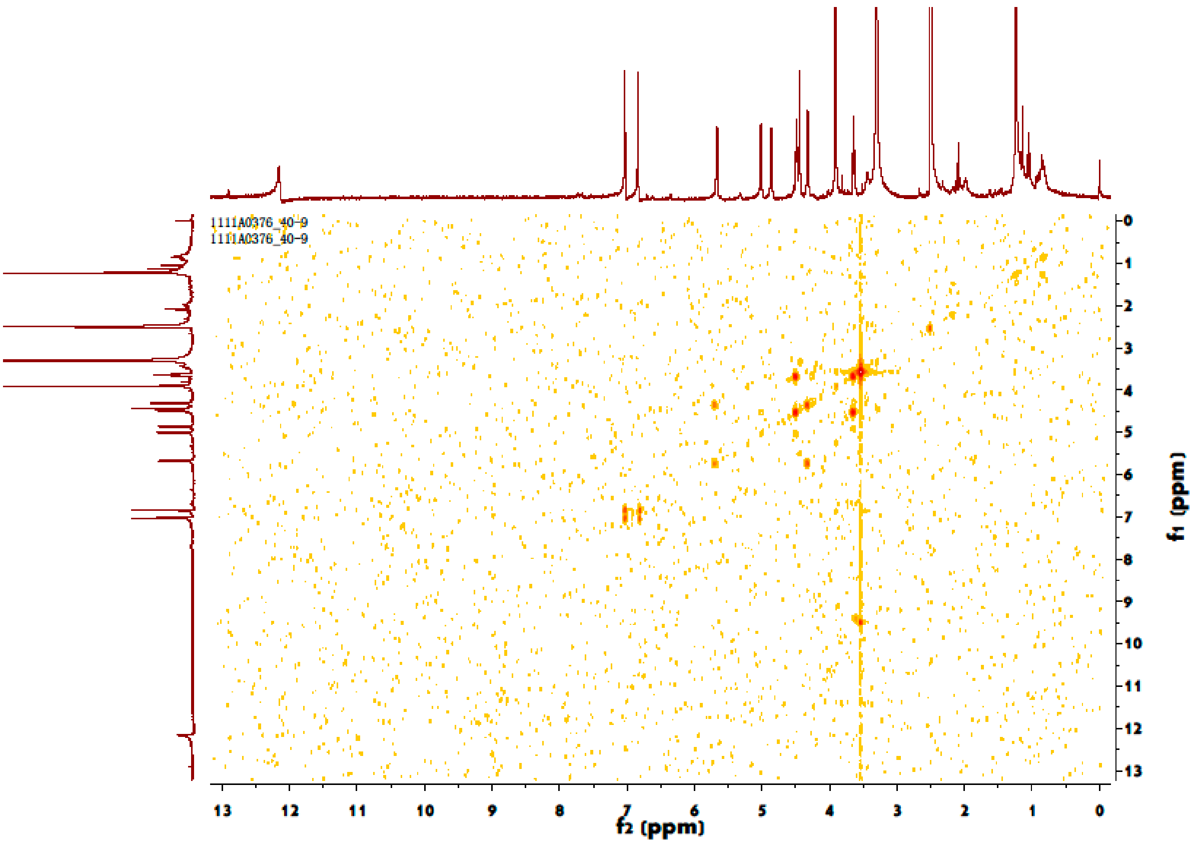

Figure S77. HSQC of compound 10, measured at 400 MHz (^{1}H) and 100 MHz (^{13}C) (DMSO- *d*6).

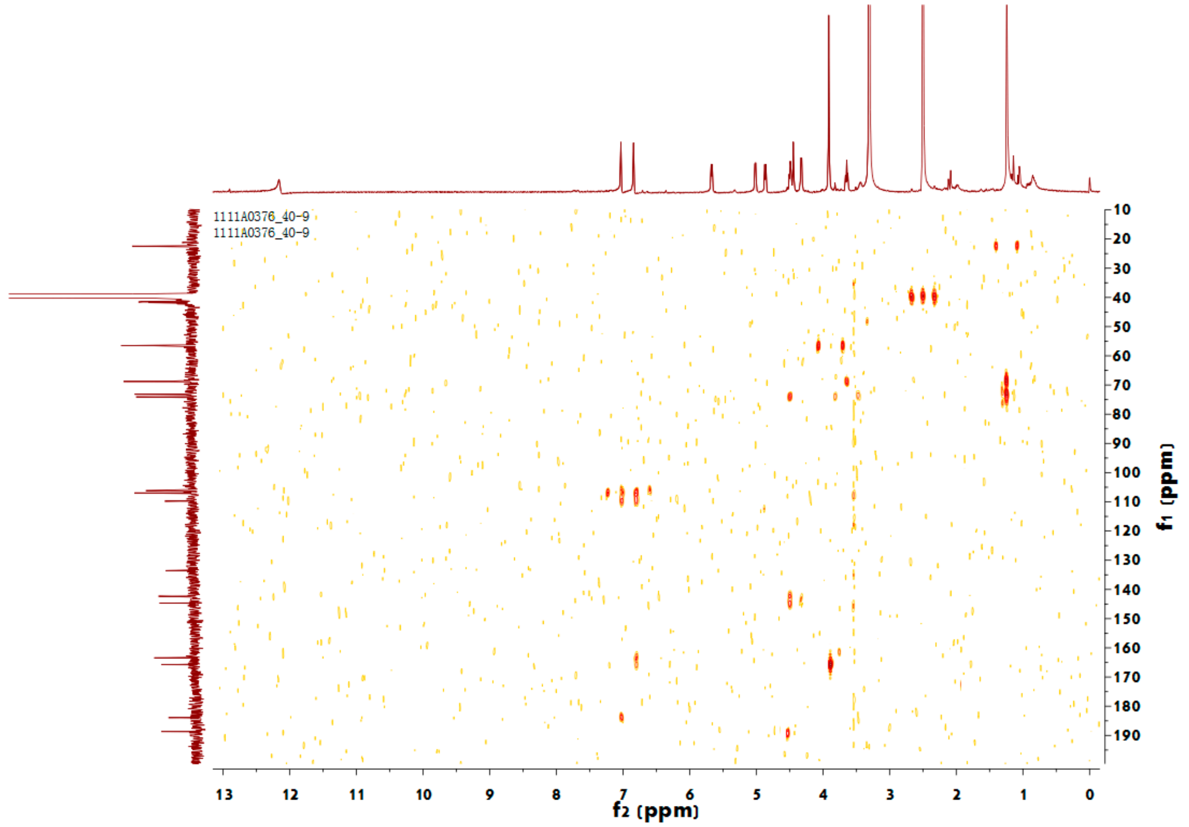

Figure S78. HMBC of compound 10, measured at 400 MHz (^{1}H) and 100 MHz (^{13}C) (DMSO- *d*6).

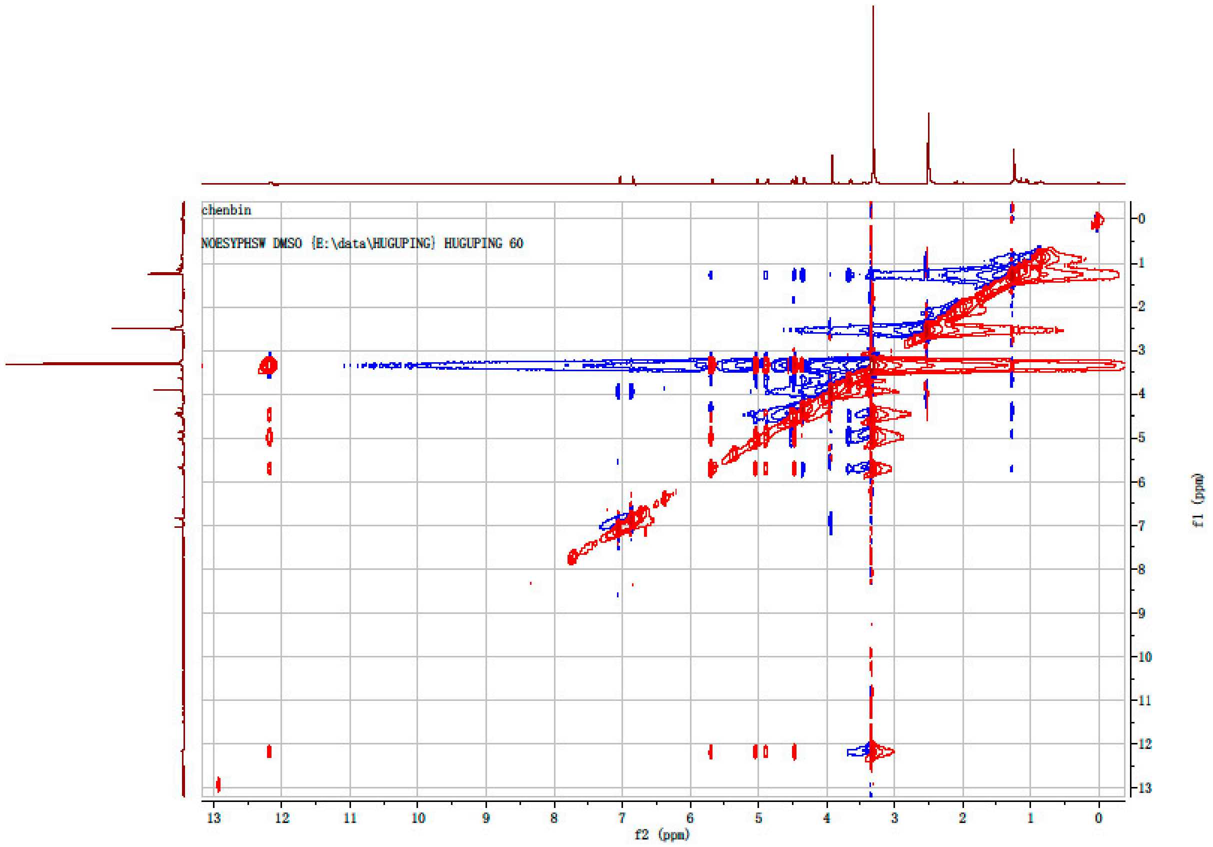

Figure S79. ESI -MS spectra of compound 10.

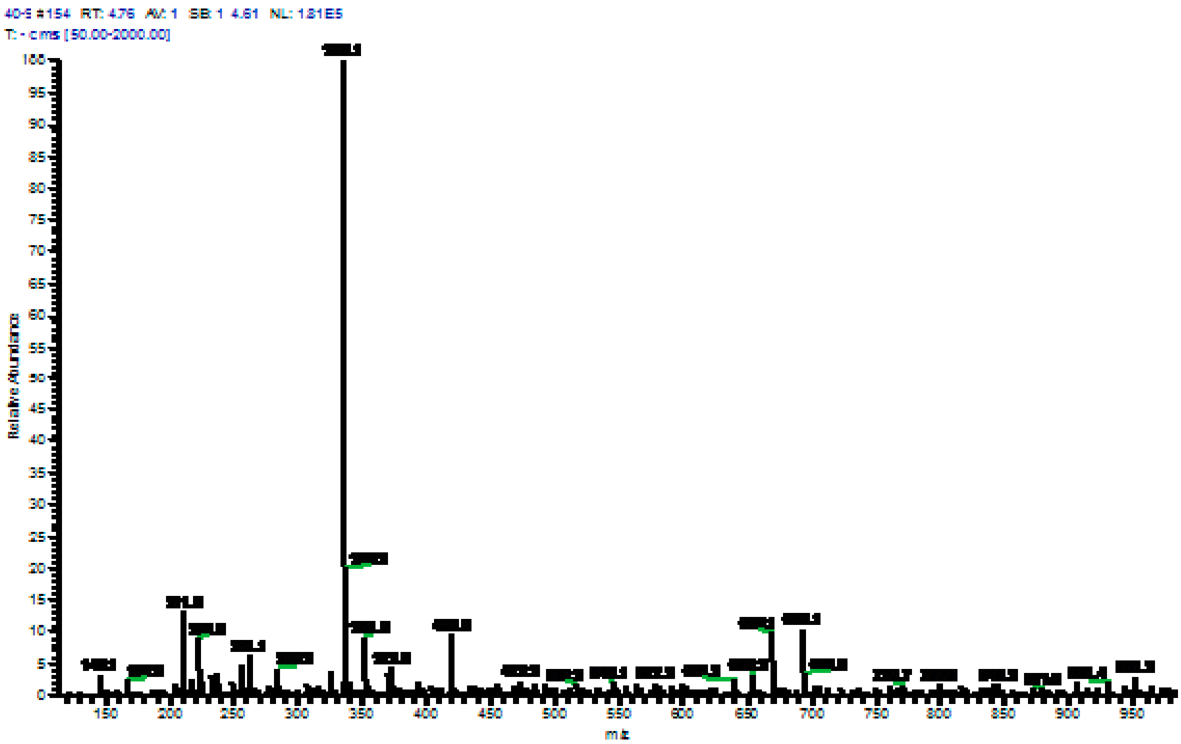

Figure S80. ^{1}H-^{1}H COSY of compound 11, measured at 400 MHz (DMSO- *d*6).

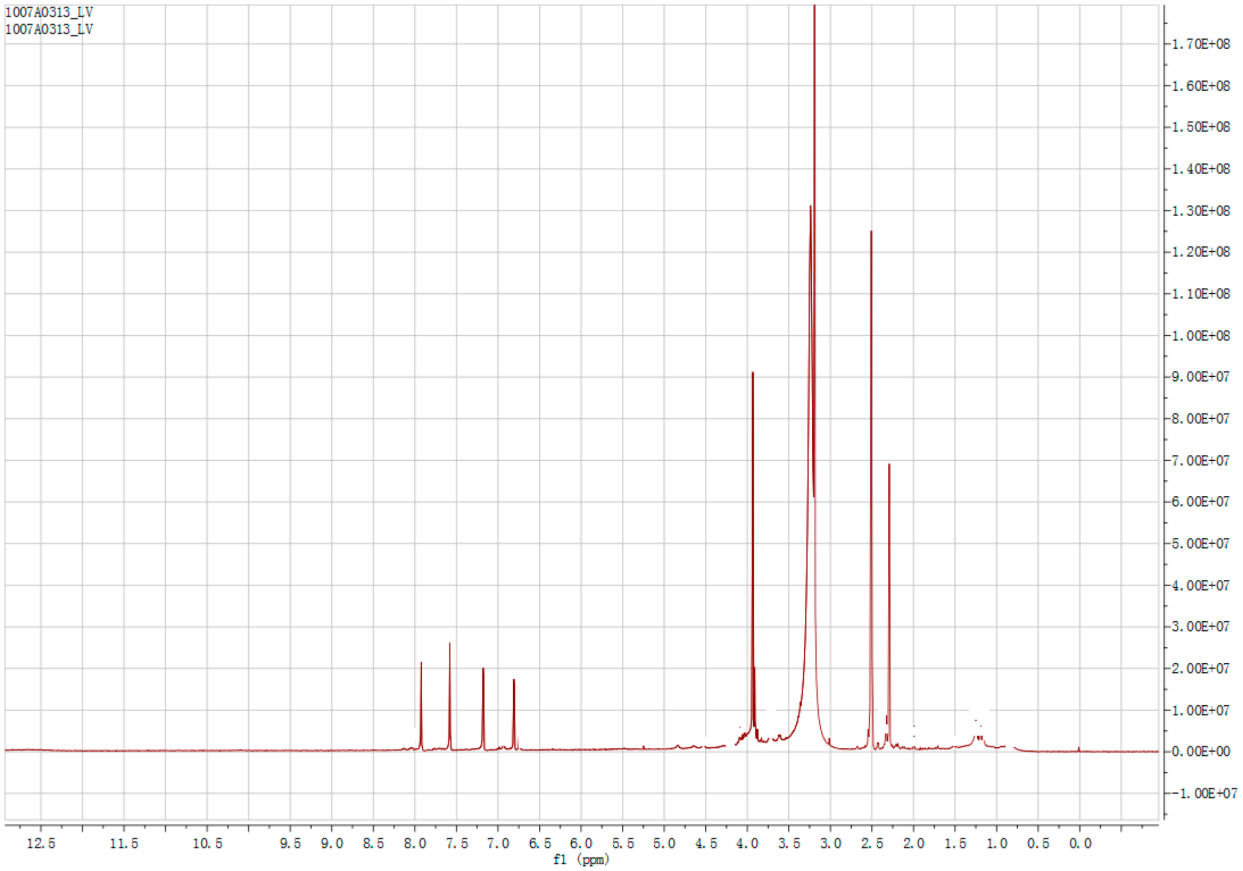

Figure S81. EI -MS spectra of compound 11.

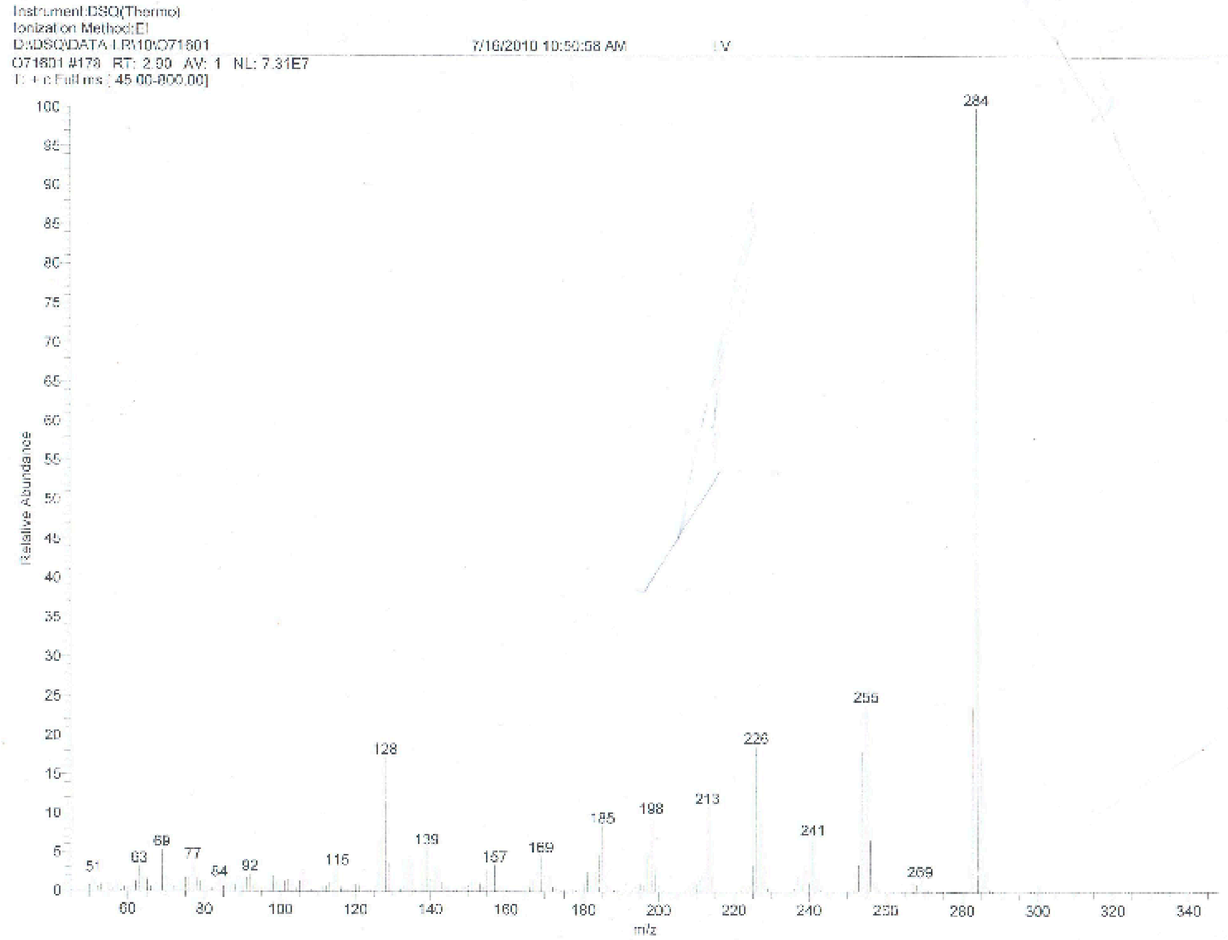

Supplement: Supplementary File 1 [file molecules-19-16529-s001.pdf]
